# From π–π Stacking to Chain Entanglements: Single Crystals of Oligoether-Substituted Thieno[3,2‑*b*]thiophenes

**DOI:** 10.1021/acs.macromol.6c00172

**Published:** 2026-03-17

**Authors:** Joost Kimpel, Iona Anderson, Di Zhu, Jyotsana Kala, Przemyslaw Sowinski, Alexander Giovannitti, Lars Öhrström, Jenny Nelson, Christian Müller

**Affiliations:** † Department of Chemistry and Chemical Engineering, 11248 Chalmers University of Technology, Göteborg 412 96, Sweden; ‡ Department of Physics, 4615 Imperial College London, London SW7 2AZ, UK; § Wallenberg Initiative Materials Science for Sustainability, Department of Chemistry and Chemical Engineering, Chalmers University of Technology, Göteborg 412 96, Sweden

## Abstract

Knowledge of the molecular arrangement in the solid state is essential for designing high-performance conjugated polymers. The crystal structures and thermal behavior of their monomers provide important insight into their chain conformation and solid-state structure. Here, single crystals of aromatic thieno­[3,2-*b*]­thiophene units bearing mono- to tetraethylene glycol chains, building blocks of some high-performance organic mixed ionic-electronic conductors, are isolated using a robust crystallization protocol and analyzed using single-crystal X-ray diffraction and thermal analysis. Increasing oligoethylene glycol chain length shifts packing from π–π stacking to chain entanglement, accompanied by a melting temperature decrease from 149 to 41 °C. Molecular dynamics simulations using force fields parametrized with density functional theory show greater crystal stability for shorter chains, consistent with stronger π–π interactions relative to chain entanglement. Single crystals of a more extended conjugated system, thiophene-flanked thieno­[3,2-*b*]­thiophene with triethylene glycol, show mixed packing motifs and significant disruption of expected S···O interactions, revealing the importance of both side chain and π–π interactions. This work can be anticipated to aid the workup of monomers for synthesis, clarify packing motifs that govern structure–property relationships in conjugated polymers, and enable force-field implementation to guide organic semiconductor design and deepen understanding of their microstructure.

## Graphical abstract

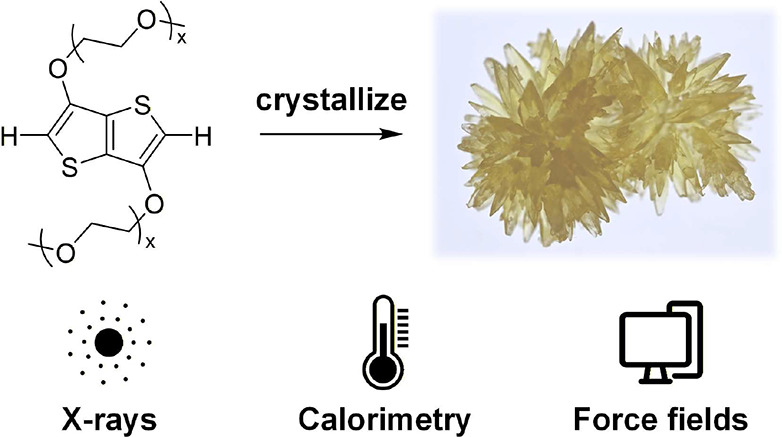

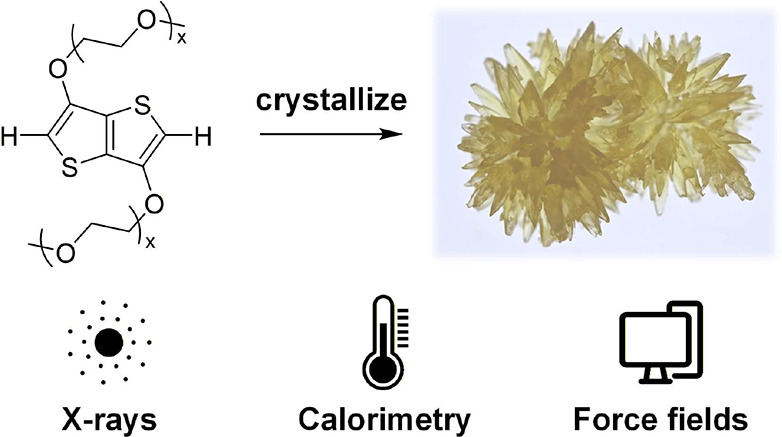

## Introduction

Conjugated polymers receive considerable interest as organic semiconductors for a wide range of applications, from organic photovoltaics to electronic circuitry for bioelectronics.^1,2^ These polymers are produced by connecting monomers, either by letting monomers of the same type to react with themselves, such as benzodifuranone in oxidative polymerization to form polybenzodifuranedione,^3^ or by combining multiple bifunctionalized monomers with orthogonal reactivity,^4^ such as organodistannanes and aryl dibromides, which cross-couple through, e.g., Stille coupling.^5^ It is essential that monomers are of high purity to rule out the occurrence of side reactions that would stop chain growth or create a stoichiometric imbalance that leads to low molecular weight polymers, as per Carothers.^6^ Moreover, many macromolecular properties ultimately originate from the behavior of the (pure) monomers. In particular, the preferred conformation and intermolecular interactions of a monomer in the solid state often foreshadow backbone conformation, packing motifs, crystallinity, and π–π stacking distances in the resulting polymer. This makes crystallization of monomers crucial as 1) it is a straightforward method for purification, 2) solids are easier to handle, 3) it can be used to obtain density functional theory (DFT) force fields for modeling of polymer chain behavior,^7,8^ and 4) monomer/oligomer crystals can provide information about interactions that cannot be gained from studying the final conjugated polymers since the latter tend to have a considerable degree of disorder.^9–11^

The most widely used route to make conjugated polymers processable from organic solvents is via flexible side chains, which disturb π–π stacking and increase the conformational entropy.^12–14^ This usually requires that at least one of the monomers carries flexible chains. Monomers with alkyl chains are most common since they offer relative ease of synthesis, and their hydrophobic nature imparts solubility in many industrial organic solvents.^13^ Monomers with alkoxy side chains feature similar behavior, i.e., they result in mostly hydrophobic materials.^9,15^ Still, considerations should be made to minimize the side-chain content as the aromatic core is the main electroactive part.^16,17^ Many monomers with alkyl side chains, especially linear alkyl chains, can be isolated as solids and thus allow recrystallization, leading to highly pure monomers.^18,19^

More recently, polymers with oligoethylene glycol side chains have attracted attention since those result in a higher dielectric constant,^20^ enable processing/swelling in more polar solvents such as acetonitrile and alcohol,^21–23^ and improve ion transport, which is of importance for, e.g., organic mixed ionic-electronic conductors (OMIECs).^24,25^ An unintended effect of attaching oligoethylene glycol chains is weakened molecular interactions and a decrease in solid state order, often indicated by a low melting temperature *T*_m_ and enthalpy of fusion. This causes many monomers with oligoethylene glycol chains to exist as viscous oils at room temperature, as indicated by many synthetic protocols,^21,25–28^ which makes handling and purification of compounds more cumbersome, an effect less pronounced when comparing to analogous alkylated systems in, e.g., oligothiophenes,^29,30^ phenyl-thiophenes,^31^ and perylene diimides.^32,33^ Attempts at melt crystallization often result in the formation of waxes that are not fully ordered and within which impurities are embedded. The higher conformational entropy also leads to more complex crystallization patterns in solution since the increased solubility in organic solvents hinders nucleation. Not only does this make oligoethylene glycol carrying monomers difficult to purify, which holds back clean synthesis and upscaling, but it is also related to the often-observed lower structural order of corresponding conjugated polymers.^15,34^

An alternative route to induce crystallization of monomers is by adding functional groups, e.g., alcohols or hydrogen bonding motifs, which can act as directing groups, or attaching heavy atoms, which give rise to additional interactions. By this method, it is possible to isolate single crystals of functionalized monomers at room temperature, e.g., stannylated compounds such as Me_3_Sn-bithiophene-SnMe_3_ with tri/tetraethylene glycol side chains.^9^ Synthesis of these heavy atom containing compounds is necessary to perform various polymerization reactions and, in addition, can also be beneficial from a crystal growth and engineering perspective.^35^ However, functionalization can considerably alter the stacking motif and is thus not representative for the polymer since the functional group is removed during the cross-coupling reaction in the case of, e.g., stannylated compounds. Among unfunctionalized aromatics, only monomers with short oligoether chains, such as 1,4-bis­[2-(3,4-ethylenedioxy)­thienyl]-benzene,^36^ or small molecules directly used as OMIECs, such as diphenylbithiophenes,^31^ have been reported. The lack of crystal structures of unsubstituted aromatics with long oligoether chains holds back a better understanding of conjugated polymer semiconductors.

Computational techniques, such as those using DFT and molecular dynamics (MD) simulations, can assist in understanding the intra- and intermolecular interactions of polymer chains and how these influence structure formation.^37^ These techniques should ideally be cross-referenced with experimental results to ascertain whether the model is valid. This is nontrivial for conjugated polymers since neither single crystals nor highly crystalline films are typically accessible, given the disorder due to backbone twist. Hence, techniques such as optical spectroscopy, grazing incidence wide-angle X-ray scattering (GIWAXS), and, in some specific cases, scanning tunneling microscopy (STM) can only provide average information for films that comprise a wide range of conformations.^38,39^ Given that polymer-structure calculations are computationally intensive and polymers have complex, variable conformations, force fields are typically developed and validated using small molecules representing a monomer or submonomer of the polymer, which may be prepared as crystals and are therefore more suited for parametrization.^40^ Accordingly, the crystal structures of monomers obtained by single-crystal X-ray diffraction (SC-XRD) can be used as a starting point to test models that describe the microstructure of polymer films.

One molecule that has recently emerged as a versatile building block for conjugated polymers is 3,6-bis­(*x*-ethylene glycol monomethyl ether)­thieno­[3,2-*b*]­thiophene (g_*x*_TT). This monomer features high reactivity in more benign direct arylation polymerization (DAP), not needing any functional groups to allow polymerization, and has led to a state-of-the-art OMIEC, p­(g_3_TT-T2) (Figure S1), with a figure-of-merit [μ*C* ^*^] > 1000 F cm^–1^ V^–1^ s^–1^ (electronic mobility μ; volumetric capacitance *C* ^*^).^41,42^ Understanding the solid-state organization of the monomer is thus directly relevant for rationalizing the microstructure of the derived polymers.

Here, we report the synthesis of a series of 3,6-bis­(*x*-ethylene glycol monomethyl ether)­thieno­[3,2-*b*]­thiophene monomers (g_*x*_TT) with *x* = 0–4, as well as the growth, isolation, and characterization of single crystal structures composed of these monomers. Force fields for this series are parametrized and used to support the variations in thermal stability within the series. Crystals of π-extended T-g_3_TT-T, the repeat unit of a state-of-the-art OMIEC,^27,41^ and its brominated form, BrT-g_3_TT-TBr, were also isolated to provide insights into intramolecular sulfur–oxygen interactions, often used as a handle for planarizing π-systems and thus for improving charge transport, and the influence of heavy bromine atoms on the crystal structure.

## Results and Discussion

We have recently observed that g_3_TT readily crystallizes at accessible temperatures,^27,41^ an unprecedented observation for monomers with oligoethylene glycol chains without heavy substituents (e.g., Br or Sn). Accordingly, a g_*x*_TT monomer series was synthesized by Ullmann-type coupling of *x*-ethylene glycol monomethyl ether to a 3,6-dibromothieno­[3,2-*b*]­thiophene core (Figure 1a,b). Purification routes of all compounds were comparable: extraction followed by column chromatography (see Supporting Information for details). The monomers with the shortest substituents, g_0_TT and g_1_TT, were isolated as crystalline solids, g_2_TT was isolated as a powder, and g_3_TT and g_4_TT were isolated as oils. Yields after workup for novel thieno­[3,2-*b*]­thiophenes g_0_TT, g_1_TT, g_2_TT, and g_4_TT were between 34 and 56%, while the previously reported g_3_TT had a yield of up to 81%.^41^ High purity of the compounds was confirmed by NMR techniques (Figures S2–S21), as indicated by a single aromatic proton signal at around 6.30 ppm and signals in the oligoethylene glycol region between 4.30 and 3.30 ppm.

**Figure 1:**
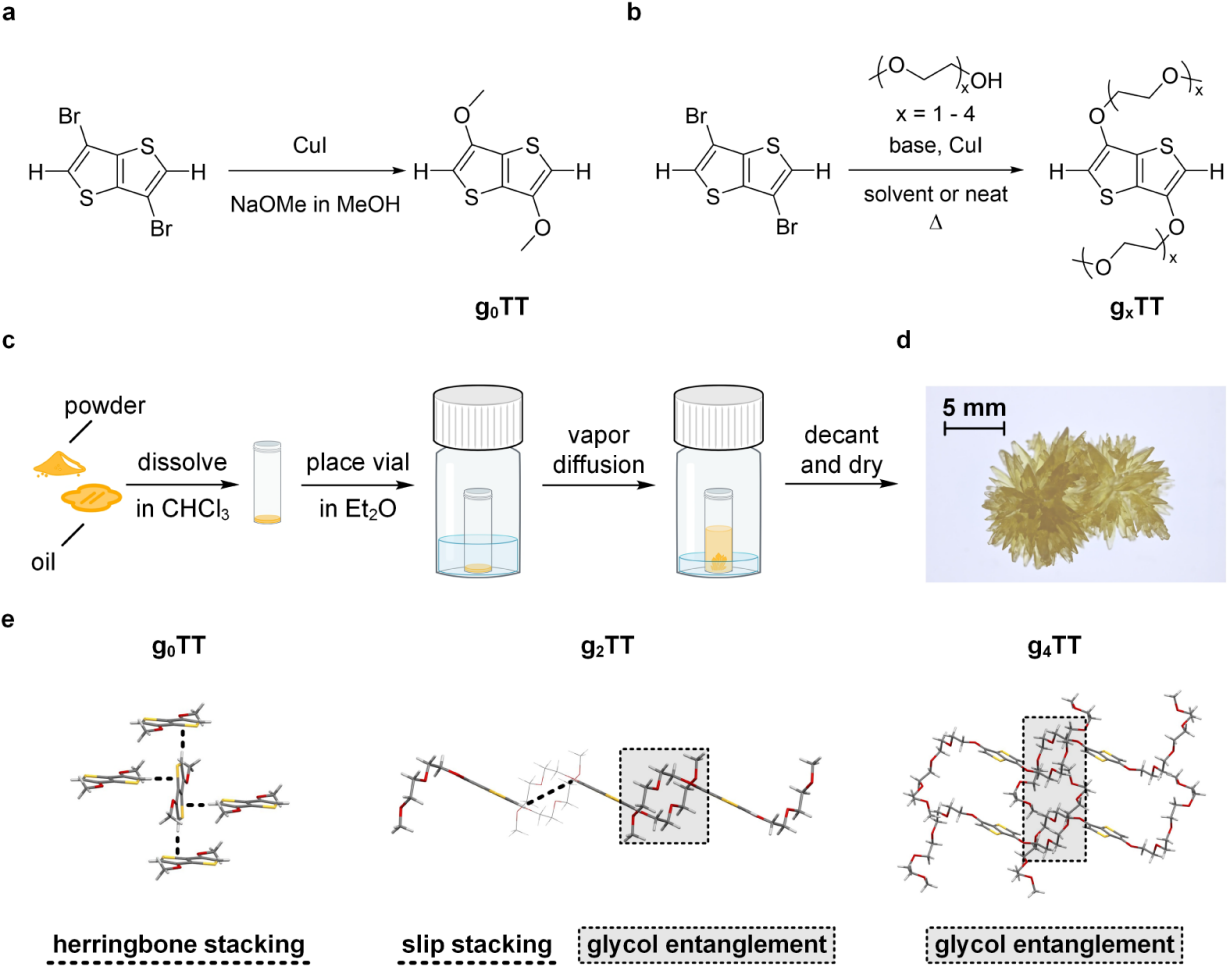
Isolation of 3,6-bis­(*x*-ethylene glycol monomethyl ether)­thieno­[3,2-*b*]­thiophene crystals. (a, b) Ullmann-type reaction used to attach *x*-ethylene glycol monomethyl ether groups to the thieno­[3,2-*b*]­thiophene core, (c) method of crystallization, (d) photograph of g_3_TT crystals, and (e) packing of g_0_TT, g_2_TT, and g_4_TT in single crystals grown from solution via vapor diffusion.

Single crystals of all compounds were grown by vapor diffusion crystallization at low temperatures (Figure 1c; see Supporting Information for details). The successful isolation of solution-grown single crystals was confirmed by single-crystal X-ray diffraction (SC-XRD) giving crystal structures of polymorphs of the g_*x*_TT series (Table 1, CCDC database 2503697–2503700 and 2504797, Figures S22–S31, and Tables S1–S5). The unit cell for polymorphs of g_0_TT to g_3_TT adopts the same *P*2_1_/*c* space group, possessing a 2_1_ screw axis, a glide plane, and an inversion center. The unit cell of isolated g_4_TT (Form II) belongs to the *P*-1 space group with lower symmetry and only possesses an inversion center. SC-XRD revealed a significant change in molecular packing from π–π herringbone stacking (g_0_TT) to π–π slip stacking (g_1_TT) to an oligoethylene glycol chain entangled mode in the case of g_3_TT and g_4_TT. The g_2_TT compound experienced intermediate π–π slip stacking with closely packed biethylene glycol chains (Figure 1e). The unit cell volume increases consistently with increasing oligoethylene size from g_0_TT to g_3_TT, namely, by ca. 250 Å^3^ per ethylene glycol repeat unit (Table 1). However, the unit cell volume only increases by ca. 200 Å^3^ from g_3_TT to g_4_TT, indicative of closer packing of molecules in the case of g_4_TT.

**Table 1:** Summarized Crystal Data of g*_x_* TT^a^

	g_0_TT	g_1_TT	g_2_TT	g_3_TT (I)	g_4_TT (I)	g_4_TT (II)
Asymmetric unit	C_4_H_4_OS (1/2 g_0_TT)	C_6_H_8_O_2_S (1/2 g_1_TT)	C_8_H_12_O_3_S (1/2 g_2_TT)	C_10_H_16_O_4_S (1/2 g_3_TT)	C_12_H_19_O_5_S (1/2 g_4_TT)	C_24_H_38_O_10_S_2_
Space group	*P*2_1_/*c*	*P*2_1_/*c*	*P*2_1_/*c*	*P*2_1_/*c*	*P*2_1_/*c*	*P*-1
*Z*	4	4	4	4	4	2
*a* (Å)	7.1506(4)	8.3423(3)	8.2828(2)	11.3374(13)	11.2187(2)	10.8044(3)
*b* (Å)	8.3788(4)	7.0346(17)	8.7695(3)	9.0953(7)	7.8259(2)	11.2170(3)
*c* (Å)	7.6478(4)	11.5647(3)	12.8067(4)	11.4388(7)	15.8787(3)	12.8614(5)
*V* (Å^3^)	425.08(4)	674.72(3)	922.50(5)	1179.28(16)	1376.23(5)	1375.88(9)
α (°)	90	90	90	90	90	65.538(3)
β (°)	111.946(6)	96.17(2)	97.393(3)	91.203(6)	99.183(2)	85.706(3)
γ (°)	90	90	90	90	90	75.968(3)

a Z = asymmetric molecule units per unit cell. Diffraction data were recorded by using CuKα radiation (λ = 1.54184 Å). Structures were solved with the ShelXT21 structure solution program using the intrinsic phasing solution method and using Olex2 and Mercury as the graphical interface. Roman numerals in brackets indicate which polymorph the peak corresponds to (cf Figure 2a and c): I = extended oligoether chain, II = hooked oligoether chain.

The edge-to-face distance in the g_0_TT π-π herringbone-stacked motif is 3.40 Å, which is a typical distance for aromatic compounds. Centroid–centroid distances between thieno­[3,2-*b*]­thiophene planes in π–π slip-stacked g_1_TT and g_2_TT are 7.07 and 8.77 Å, respectively. The distance between the closest atoms within these aromatic planes increases from 3.85 Å for g_1_TT (strong π–π interactions) to 5.46 Å for g_2_TT (weak π–π interactions). These distances indicate that the propensity for π–π stacking decreases with increasing oligoether chain length. In the case of g_3_TT and g_4_TT with even longer oligoether chains, π–π stacking was completely absent, as evidenced by the lack of a clear arrangement of aromatic cores in a π–π herringbone stacking or π–π slip stacking motif. Instead, we deduce an angle of 35° between the thieno­[3,2-*b*]­thiophene planes. Moreover, the shortest centroid–centroid distances were over 9.30 Å, and the closest atom distances between aromatic cores were over 6.75 Å.

The tendency of interactions between oligoethylene glycol chains and the aromatic cores is exemplified by the observed evolution of crystal packing. For longer chains, crystal structures showcase an intermixing of oligoethylene glycol chains and aromatic cores. In contrast, crystals of aromatic molecules with alkyl chains such as those of 3,6-dihexylthieno­[3,2-*b*]­thiophene tend to feature separated alkyl and aromatic domains.^18^ To confirm that the same trend persists for longer chains in thieno­[3,2-*b*]­thiophene-based compounds, we synthesized 3,6-bis­(decyloxy)­thieno­[3,2-*b*]­thiophene (aTT),^43^ the alkoxylated analogue of g_3_TT, and grew single crystals from solution (CCDC database 2503701, Figures S32–S33, Table S6). Indeed, aTT shows π–π herringbone stacking with separate alkyl and aromatic domains. The edge-to-face distance between aromatic cores was 3.30 Å, indicative of strong π–π interactions (similar to that in g_0_TT). Clearly, replacing relatively polar oligoether chains with apolar alkoxy chains significantly enhances the relative strength of π–π stacking in the crystals (Figure S34). This is further illustrated by Hirshfeld plots of crystal structures, where aTT shows strong π–π stacking, and g_0_TT to g_2_TT exhibit comparatively weaker π–π stacking, while g_3_TT and g_4_TT feature broad dispersion forces with close-proximity aromatic-oligoether chain spikes (Figure S35).

To gain insights into the interactions within the crystals and the presence of polymorphs, solution-grown single crystals were subjected to multiple heating and cooling cycles and studied with differential scanning calorimetry (DSC) (Figure 2a; Figure S36). For validation of MD simulations, understanding the stability of crystal structures, which can be compromised by competing interactions, is imperative. Moreover, in the case that a compound possesses multiple stable polymorphs, these must be taken into consideration. First DSC heating thermograms provided information about solution-grown polymorphs. A single melting endotherm was observed, with *T*_m_ decreasing from g_0_TT to g_3_TT followed by an increase in the case of g_4_TT. Evidently, compounds g_0_TT and g_1_TT whose crystals feature strong π–π stacking show a high *T*_m_ ≃ 140–149 °C compared to g_3_TT and g_4_TT with only glycol entanglements and hence a lower *T*_m_ ≃ 56–64 °C. An intermediate *T*_m_ = 97 °C was observed for g_2_TT since it experiences both interactions. Melting was also visually confirmed for g_3_TT on a Koffler bench, which revealed that the compound transitions from a solid to a liquid state, rather than a change in solid polymorph (Figure 2b). DSC second heating thermograms revealed single melting endotherms for g_0_TT to g_2_TT, while thermograms recorded for g_3_TT and g_4_TT featured two melting endotherms. This suggests the existence of one dominant polymorph for g_0_TT to g_2_TT and possibly two polymorphs for g_3_TT and g_4_TT.

**Figure 2:**
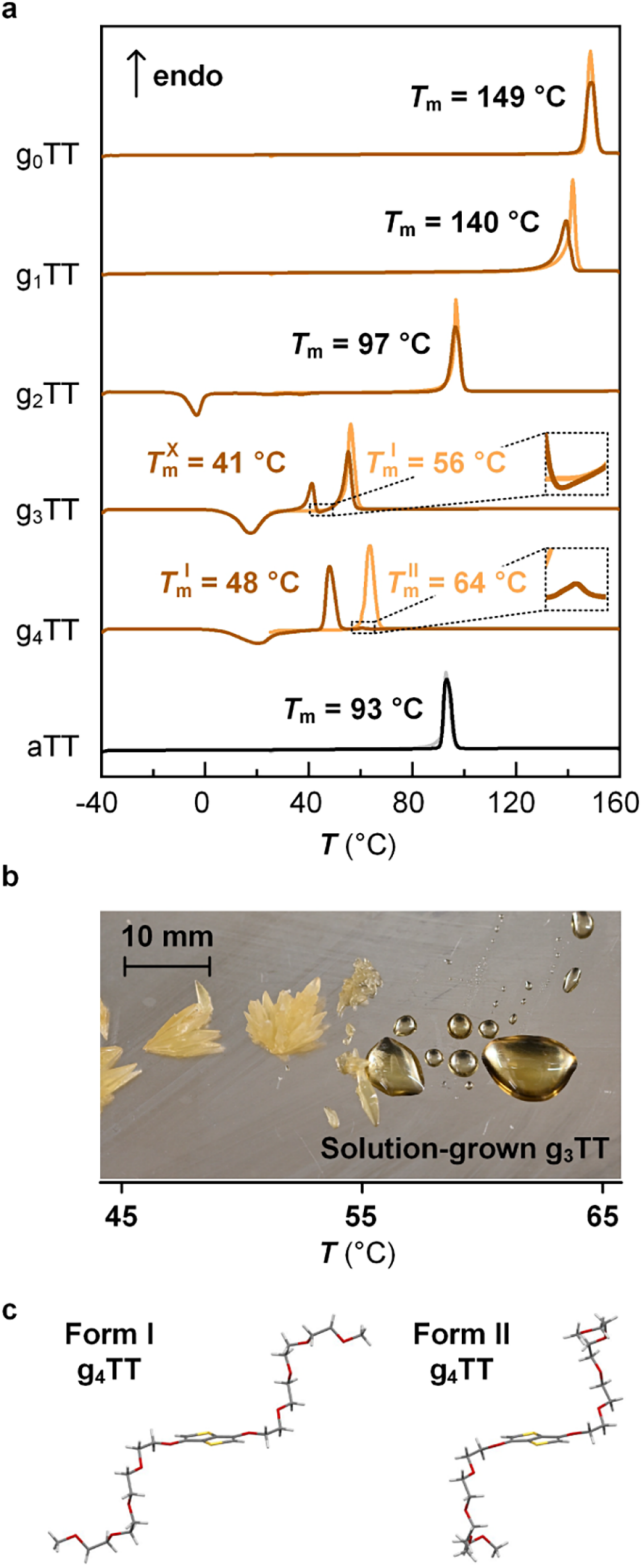
Melting of crystals. (a) Differential scanning calorimetry (DSC) heating thermograms of thieno­[3,2-*b*]­thiophene crystals (first heating in pale orange/gray; second heating in brown/black); insets for g_3_TT and g_4_TT thermograms show small exo/endotherms; (b) melting of Form I g_3_TT on a Koffler bench; and (c) Form I and II polymorphs of g_4_TT.

The first DSC cooling thermograms of g_0_TT and g_1_TT show sharp crystallization exotherms with *T*_c_ = 102 and 108 °C, respectively, whereas cooling thermograms recorded for g_2_TT to g_4_TT show no exotherm. Noteworthy is that crystal structures that possessed an oligoether chain entanglement mode, i.e., g_2_TT to g_4_TT, gave rise to clear exotherms in second heating thermograms (Figure 2a). The lack of any exotherm in the cooling thermograms, but the presence of exotherms in the second heating thermograms, suggests the formation of nuclei during cooling followed by crystal growth during heating, i.e., cold crystallization (cc).^44,45^ The peak cold crystallization temperatures increased from *T*_cc_ = −4 to 18 to 20 °C, accompanied by a broadening of the peak with every additional ethylene glycol unit.

DSC first and second heating thermograms of aTT featured an endotherm with *T*_m_ = 93 °C (Figure 2a), comparable to g_2_TT, and the DSC first cooling thermogram showed a sharp crystallization exotherm, like g_0_TT and g_1_TT, with *T*_c_ = 69 °C. The strong π–π interactions in aTT allow the alkyl chains to pack similarly to polyethylene (PE), as confirmed by a similar conformation and similar distances compared to the unit cell size of monoclinic PE (*a* × *b* × *c* = 5.14 Å × 4.87 Å × 2.55 Å with β ≃ 111° for alkyl chains in aTT and 8.09 Å × 4.79 Å × 2.53 Å with β ≃ 108° for the PE unit cell).^46^ Moreover, the alkyl chains do not seem to disturb the π–π interactions. Unlike g_2_TT to g_4_TT, aTT showed no exotherms in the second heating cycle. The main difference in structure is that aTT showcases close π–π herringbone stacking and separate PE-like domains. This is in contrast to long distance π–π slip stacking and biethylene glycol chain interactions in g_2_TT crystals and the oligoethylene glycol chain interactions in g_3_TT and g_4_TT crystals. Cold crystallization is only observed in the case of moieties with oligoethylene glycol chains.

Multiple melting peaks in the second heating thermograms of units with longer oligoethylene glycol chains, g_3_TT and g_4_TT, suggest multiple stable polymorphs. The small exotherm that follows the first endotherm in the second heating cycle in g_3_TT (Figure 2a inset of g_3_TT, Figure S37) indicates overlapping melting and secondary crystallization events. The disparity between cold crystallization and melting enthalpies, Δ*H*_cc_ < Δ*H*_m,2low_ + Δ*H*_m,2high_ < Δ*H*_m,1_ (Table 2, Figure S37), indicates that some crystallization occurs during cooling. For g_4_TT, the higher *T*_m_ endotherm at 64 °C is almost entirely suppressed and replaced with a lower *T*_m_ endotherm at 48 °C (Figure 2a, inset of g_4_TT). This is accompanied by a decrease in relative crystallinity *χ*_*c*_ to 74% in subsequent heating thermograms, contrary to g_0_TT to g_3_TT, which retain *χ*_*c*_ > 90% (Table 2).

**Table 2:** Melting and Crystallization of g_3_TT and g_4_TT^a^

	First heating		Second heating						
	*T*_*m*,1_(°C)	Δ*H*_*m*,1_ (J g^–1^)	*T*_cc_ (°C)	Δ*H*_cc_ (J g^–1^)	*T*_*m*,2low_ (°C)	Δ*H*_*m*,2low_ (J g^–1^)	*T*_*m*,2high_ (°C)	Δ*H*_*m*,2high_ (J g^–1^)	*χ_c_* (%)
g_3_TT	56 (I)	20.8 (I)	18	– 16.9	41 (I)	6.2 (I)	55 (I)	12.7 (I)	91
g_4_TT	64 (II)	26.1 (II)	20	– 16.9	48 (I)	19.1 (I)	61 (II)	0.1 (II)	74

a Obtained from first and second heating thermograms. Peak melting and cold crystallization temperatures given as *T*_m_ and *T*_cc_. Associated enthalpy changes (Δ*H*_m_ and Δ*H*_cc_) were determined by peak integration. Subscripts indicate the heating cycle (1 for first and 2 for second) and endotherm location (low for lower *T* and high for higher *T*). Roman numerals in brackets indicate which polymorph the peak corresponds to (cf Figure 2a and c): I = extended oligoether chain, II = hooked oligoether chain. Relative crystallinity (*χ_c_*) determined by comparing melting enthalpies of subsequent cycles: *χ*_*c*_ ≅ ((Δ*H*_*m*,2low_ + Δ*H*_*m*,2high_)/(Δ*H*_*m*,1_)) × 100%) assuming that both polymorphs have the same enthalpy of fusion.

Melt crystallization was attempted to isolate the crystals of g_3_TT and g_4_TT with different *T*_m_ values (Figure 2a; Figure S37). Molten material was cooled from 160 to −40 °C using a cooling rate of 10 °C min^–1^. This was then followed by an isotherm at 45 °C to isolate the higher *T*_m_ crystals for g_3_TT, an isotherm at 50 °C to isolate the higher *T*_m_ crystals for g_4_TT, or an isotherm at 20 °C to isolate the lower *T*_m_ crystals for both g_3_TT and g_4_TT. Each isotherm had a duration of 1 h, at which point no more heat flow was detected. Melt-grown crystals of all species could be isolated and were subsequently analyzed with SC-XRD. Crystal structures of higher *T*_m_ melt-grown crystals confirmed that they correspond to the same polymorph as solution-grown crystals with the same *T*_m_. In the case of g_3_TT, the lower *T*_m_ melt-grown crystals gave the same polymorph, meaning that this lower *T*_m_ may arise from smaller crystals or from a multimorphic mixture of different polymorphs. Conversely, the lower *T*_m_ melt-grown crystals of g_4_TT showcased a different molecular conformation and a completely different crystal packing, i.e., a different polymorph.

We compared the packing of oligoether chains in different polymorphs of g_*x*_TT to the chain conformation within poly­(ethylene oxide) (PEO) crystals (Figure S38). Single crystals of PEO comprise chains with a 7_2_-helix conformation identical to the oligoether chains in some members of the g_*x*_TT series and have a *T*_m_ ≃ 65 to 70 °C.^47^ The single crystal structures of g_*x*_TT reveal that the oligoether chains in solution-grown g_1_TT, g_2_TT, melt-grown/solution-grown g_3_TT crystals, and the melt-grown lower *T*_m_ polymorph of g_4_TT adopt a 7_2_-helix conformation like PEO. Previously, these conformations were inferred from scanning tunneling microscopy images of conjugated polymers with oligoether side chains, but they have not yet been confirmed.^34^ On the other hand, oligoether chains in the melt-grown/solution-grown higher *T*_m_ polymorph of g_4_TT show a 180° deviation from the 7_2_-helix. In the latter case, the oligoethylene glycol chain resembles a fishhook, whereas the former cases are characterized by an extended chain structure. Accordingly, we define two polymorphs: an extended oligoether chain (Form I) and a hooked oligoether chain (Form II) (Figure 2c).

The unit cell of the newly isolated Form I g_4_TT crystals belongs to the *P*2_1_/*c* space group, similar to all other extended chain species, which is of higher symmetry than the *P*-1 space group of higher *T*_m_ Form II g_4_TT (Table 1, CCDC database 2504797, Figures S28–S29, Table S4). While a higher symmetry is often associated with higher order, improved packing, stronger interactions, and ultimately a higher *T*_m_,^48–50^ this trend is not observed for g_4_TT. The volume of the unit cell remains constant, despite the change in symmetry. One striking point is that the shortest centroid–centroid distance and closest atom distance between aromatic cores in Form I g_4_TT crystals are 7.83 and 3.97 Å, respectively, which makes these π–π slip stacking distances even shorter than those observed for g_2_TT crystals. Despite this, Form I has a *T*_m_ ≃ 48 °C. This is 49 °C lower than that of g_2_TT and 16 °C lower than that of Form II g_4_TT which lacks any π–π stacking. Regardless of the degree of π–π stacking, the dominant interaction is between oligoethylene glycol chains. Hence, we assign the higher melting temperature of the less symmetric, π–π-stacking-devoid Form II g_4_TT crystals to the increased number of short-distance interactions, which involve the dipoles between carbon–oxygen bonds. The number of van der Waals (VdW) contacts with other oligoether chains, determined using a comparison with theoretical VdW radii embedded into the used crystallography software,^51^ increases from 6 to 14 per tetraethylene glycol chain (11 to 17 closest contacts per chain when also including aromatic-oligoether interactions) when moving from Form I to Form II. This argument is also consistent with *T*_m_ of Form I g_3_TT crystals, which have *T*_m_ between those of Form I and Form II g_4_TT and an intermediate number of 13 VdW contacts (13 closest contacts per chain when also including aromatic-oligoether interactions).

MD simulations provide information about the structure and dynamics of molecules, such as conformations sampled and molecular diffusion, and can be used for, e.g., understanding small molecule aggregation in solution or interactions of small molecules with biological material.^52,53^ For conjugated polymers, MD can be used for understanding essential processes such as phase transitions,^54^ packing behavior,^25,55^ and swelling behavior in the presence of water.^9^ Validated force fields are essential for producing reliable simulations, and in the case of conjugated polymers, standard force fields often need to be refined using quantum chemical methods.^56,57^ Here, force fields for the g_*x*_TT-series are defined to gain additional information about the stability and assembly of the crystals that they form.

Starting with Optimized Potentials for Liquid Simulations (OPLS), DFT was applied to find partial charges and torsional potentials for each g_*x*_TT variant (see Supporting Information for details, Figures S39–S40, and Tables S7–S15). Since the thieno­[3,2-*b*]­thiophene core is a rigid body, there are limited degrees of freedom that require parametrization. However, the oligoethylene glycol chains can adopt many conformations, and therefore, correct parametrization of the rotation of each chain about the oxygen bonded to the thieno­[3,2-*b*]­thiophene unit is critical for developing an accurate force field. We adjusted the strength of the torsional potential for the dihedral angle around the bond connecting the thieno­[3,2-*b*]­thiophene core to the oligoethylene glycol chain (Figure S39). Increasing the rigidity of this degree of freedom facilitates a more stable replication of the experimentally resolved crystal structure. However, this can result in the molecule being too highly tuned to the crystal, thus effectively trapping the molecule within the crystal structure. Hence, care was taken not to overfit this degree of freedom. To avoid a similar overfitting of electrostatic interactions, partial charges from the unit cell conformations were compared to the optimized geometry for a lone g_0_TT monomer and previously defined oligoethylene glycol partial charge parameters in PEO crystals (Tables S7–S12).^34^ To validate our force field parameters, we performed a series of simulations to test the stability of the molecular crystals when modeled using the developed force field at different temperatures. In all cases, our force field was able to preserve the solution-grown crystal structures to within an RMSD of 1.5 Å for up to 35 ns at −180 and −130 °C, i.e., temperatures close to the temperatures at which SC-XRD measurements were carried out (−157 to −143 °C; Figure 3). This was a promising sign of the validity of the developed force field.

**Figure 3:**
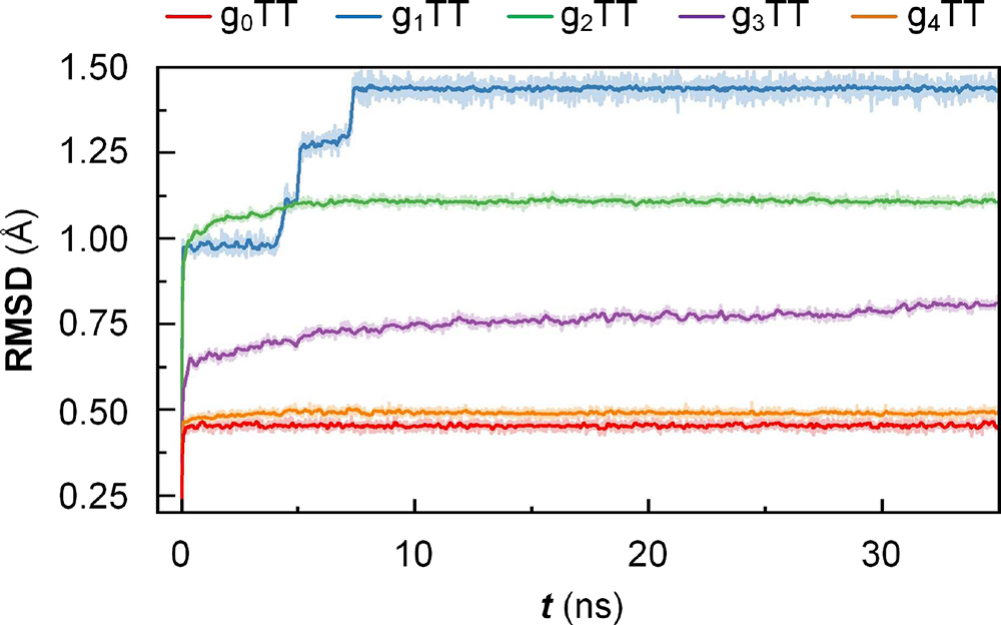
Force validity tests. Stability of simulated structures in force fields over time at −130 °C. Solid lines are averaged lines over 15 points. Root-mean-square deviation (RMSD) of simulated atomic positions compared to atomic positions from single crystal structures. RMSD is defined as the square root of the mean squared error between atomic positions $\sqrt{\overset{n}{\underset{i=1}{\sum }}{\left(r_{\exp,i}-r_{\mathrm{sim},i}\right)}^{2}}$ , where *i* represents specific atoms.

To further probe the capability of the obtained force field, higher temperatures were also investigated across the temperature range of recorded DSC thermograms to understand how well the force field can replicate the thermal stability of the crystal structures (Figure S41). The crystal structures of g_0_TT to g_2_TT remain intact up to the highest investigated temperatures of 70 °C, in line with the experimental results, i.e., the crystals did not melt within the temperature range explored in MD simulations. For g_3_TT and g_4_TT, the MD simulations predict stable crystals at −180 and −130 °C but not at higher temperatures. A plausible explanation for the difference in stability is the type of interactions for each species observed experimentally. Interactions for the shorter oligoether chain aromatics g_0_TT to g_2_TT are determined by π–π stacking. In contrast, for g_3_TT and g_4_TT, interactions between oligoether chains dominate, and those possess many conformations, and hence, crystals are less stable.

As the polymer chain grows, additional effects begin to influence the solid-state packing. Accordingly, we synthesized and analyzed the crystal structure of the repeat unit of p­(g_3_TT-T2)^41^ to evaluate the effect of backbone extension on the crystal structure. This system, which more closely approximates p­(g_3_TT-T2), still crystallizes and is hence suitable for SC-XRD. The repeat unit was synthesized by *in situ* stannylation of g_3_TT to form a highly reactive species, which could then be used for Stille coupling with 2-bromothiophene to yield T-g_3_TT-T (Figure 4a, Figures S42–S46; see Supporting Information for details). Single crystals were obtained by the same vapor diffusion method as for the g_*x*_TT series (CCDC database 2504799, Figure S47, Table S16). The resulting solution-grown crystals exhibited π–π herringbone stacking between two T-TT-T aromatic cores and entanglements between four oligoether units throughout the crystal (Figure S46). The observed packing is different from that of g_0_TT to g_2_TT crystals, which feature continuous π–π stacking. It also differs from g_3_TT to g_4_TT crystals, which comprise aromatic units embedded in a matrix of oligoethylene glycol chains. The crystal packing of T-TT-T also does not match that of aTT, which showed separate aromatic and alkyl-chain domains. Neither does it match previously reported single crystals of equivalents with alkyl chains on the thiophenes, i.e., 2,5-bis­(3-alkylthiophen-2-yl)­thieno­[3,2-*b*]­thiophenes (BTTT).^58^ The edge-to-face distance within the T-g_3_TT-T pair of aromatic cores is 4.18 Å, indicating a relatively strong π–π interaction. The closest distance between pairs of individual π–π stacks was found to be 9.60 Å. Conversely, ethylene glycol chains between different stacks are very close, with carbon–oxygen distances between chains being as low as 3.77 Å, which is even lower than the distances between the chains in PEO crystals. Interestingly, the mixed packing might be competitive (short-range π–π herringbone stacking versus oligoethylene glycol entanglement) given the lower melting endotherm of T-g_3_TT-T compared to g_3_TT, namely *T*_m_ ≃ 32 °C compared to *T*_m_ ≃ 56 °C (Figure S48). Neither this nor any other melting endotherm is recovered in further heating/cooling cycles. This demonstrates that extending the conjugated core and changing the side chain both perturb the packing; specifically, oligoethylene glycol chains hinder continuous π–π stacking and *vice versa*.

**Figure 4:**
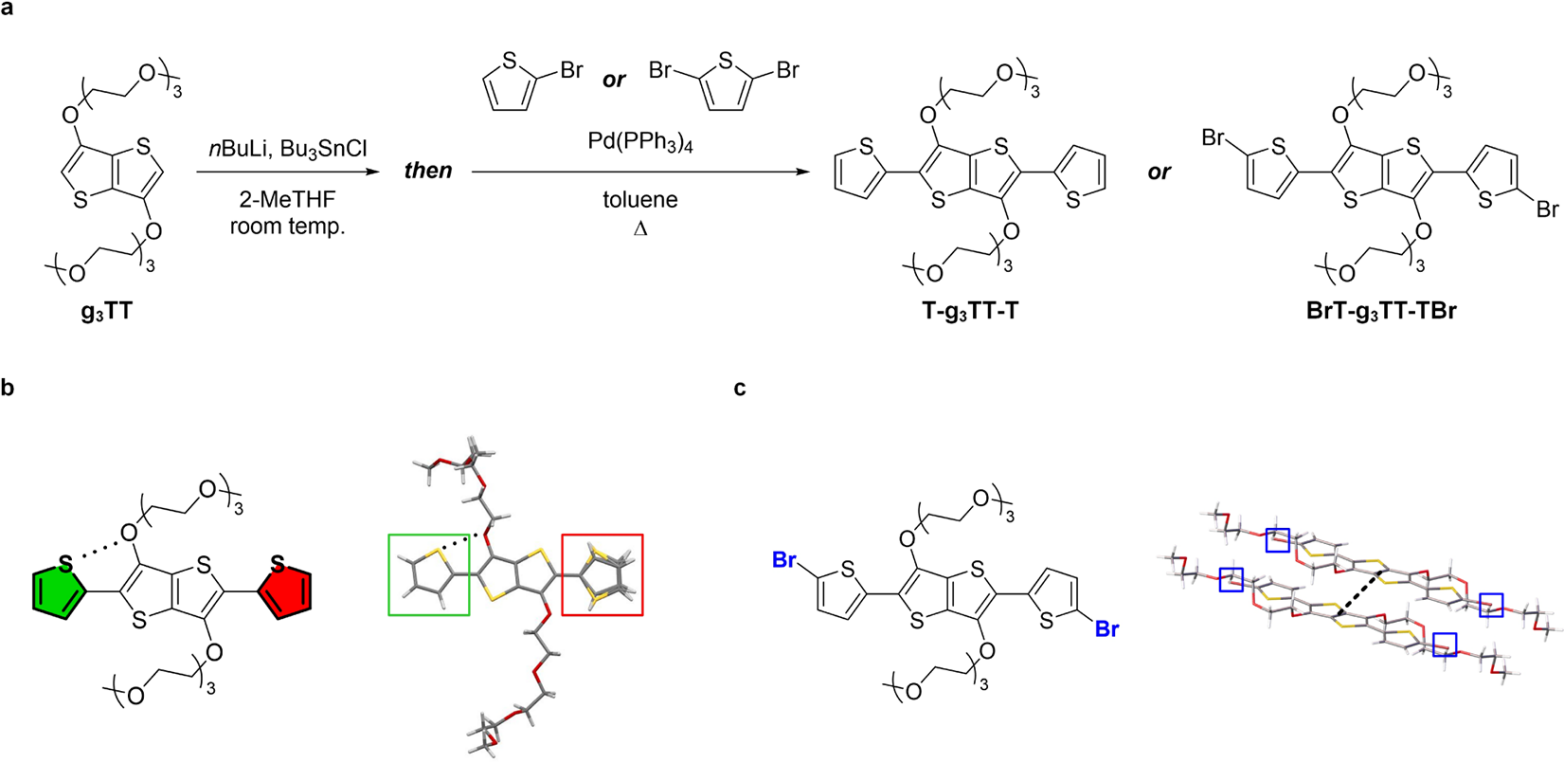
Extended structures of g_3_TT derivatives. (a) Synthesis of thiophene- and bromothiophene-flanked g_3_TT, (b) T-g_3_TT-T structure with S···O interactions (dotted) and its observed molecular conformation in a single crystal (thiophenes with/without S···O interactions are highlighted with green/red), and (c) structure of BrT-g_3_TT-TBr and packing of two molecules in a single crystal, where blue boxes indicate bromines and the dotted line represents slip stacking between aromatic systems.

To further probe the presence of conformational locking via sulfur–oxygen (S···O) interactions, a design strategy often invoked for the planarization of conjugated polymers with oligoether side chains,^34,59^ we analyzed the conformational distribution that T-g_3_TT-T can experience within the crystal structure (Figure 4a,b). Unexpectedly, the crystal structure of T-g_3_TT-T revealed that 12.5% of thiophene rings adopt a *syn* conformation relative to the thieno­[3,2-*b*]­thiophene core, which cannot sustain an S···O interaction. Thiophenes prone to adopting a *syn* conformation are surrounded by an oligoethylene glycol arc from an adjacent T-g_3_TT-T unit (Figure S49). These thiophene-oligoether dipoles hinder S···O contacts, similar to dipole-driven *syn*/*anti* mixing hindering S···F contacts in 4,7-dithieno-2,1,3-benzothiadiazole.^60^ A recent computational work by Thorley and Nielsen brought the strength of conformational locking from the S···O interaction into question, arguing that conjugation extension, steric repulsions, and the solubility of side chains can have a much greater effect on the degree of planarization of conjugated polymer backbones.^61^ Here, we provide direct experimental proof that the thermodynamically most stable form of a representative monomer comprises both an *anti*-conformation with S···O interactions and a *syn* conformation without S···O interactions. It is plausible that polymer structures, especially when solubilized at higher processing temperatures for, e.g., coating/casting, also show mixed conformations and thus are not necessarily “conformationally locked”.

Finally, to assess whether the packing motifs identified above are intrinsic to the oligoethylene glycol-substituted T-TT-T core or biased by the presence of heavy atoms commonly used in monomer synthesis (and subsequent modeling), we synthesized BrT-g_3_TT-TBr in a similar fashion as T-g_3_TT-T using excess 2,5-dibromothiophene instead of 2-bromothiophene (Figure 4a, Figures S50–S54; see Supporting Information for details). Crystals were again grown by vapor diffusion (CCDC database 2504798, Figure S55, Table S17). SC-XRD of single crystals revealed a striking change in molecular packing (Figure 4c): continuous π–π slip stacking was observed with a centroid–centroid distance of 4.39 Å and a plane–plane distance of only 2.59 Å. (The latter is more relevant here because the thiophene of one unit hovers over the thieno­[3,2-*b*]­thiophene of another unit.) While adopting the same 7_2_-helix conformation, the oligoethylene glycol chains are now in separate domains from the aromatic cores. In DSC, the first heating thermogram exhibits a melting endotherm at *T*_m_ ≃ 92 °C. The second heating thermogram shows cold crystallization between *T*_cc_ ≃ 5–30 °C, followed by a broad endotherm with a peak at *T*_m_ ≃ 52 °C (Figure S56). The higher initial *T*_m_ suggests more stable grown single crystals governed by continuous π–π stacking, whereas the lower *T*_m_ after recrystallization from the melt indicates the crystal structure to be dominated by the oligoethylene glycol chains, consistent with the similar *T*_m_ of g_3_TT (≃ 56 °C). Compared to T-g_3_TT-T, the brominated form only possesses *anti*-conformations of thiophenes, possibly owing to the inability of oligoethylene glycol chains to envelop thiophenes and disturb their conformation. While crystallization of heavy atom-bearing monomers is useful for yielding high-purity monomers, their crystal structures ought to be avoided as guidance for modeling, as interactions may be overrepresented and accordingly misrepresented.

## Conclusions

A series of 3,6-bis­(*x*-ethylene glycol monomethyl ether)­thieno­[3,2-*b*]­thiophene monomers for conjugated polymers, g_*x*_TT, was synthesized and analyzed by SC-XRD. Chain length extension shifted packing from π–π stacking with melting temperatures of 140–149 °C to chain-entangled motifs with lower melting temperatures of 41–64 °C. Such packing preferences at the monomer level are expected to influence backbone conformation and stacking and ultimately the solid-state order of the corresponding polymers. This behavior is vastly different from that of alkoxylated equivalents, in which case the alkoxy chains and the thieno­[3,2-*b*]­thiophene cores pack in separated domains. For the longest chains, i.e., tetraethylene glycol, two polymorphs were identified, characterized by extended and hooked chains, respectively, indicative of the strong interactions between ethylene glycol side chains. The presence of multiple solid-state arrangements highlights the conformational flexibility also accessible to repeat units of polymeric materials. Crystals of the p­(g_3_TT-T2) conjugated polymer repeat unit motif, T-g_3_TT-T, showed mixed π–π stacking and chain entangled packing where not all parts form S···O interactions, as often assumed in “conformationally locked” designs. Its brominated form, BrT-g_3_TT-TBr, showcased strong π–π stacking, highlighting the impact of heavy substituents on the crystal structure. Accordingly, we recommend that extrapolation of the polymeric system be done using the crystal structures of unfunctionalized systems. Force fields of the g_*x*_TT series were defined and indicated more stable systems for shorter side chains, in line with the strength of π–π stacking versus chain entangling. The acquired knowledge from this compiled library of oligoethylene glycol-substituted monomer single crystal data will aid purification efforts during the synthesis of monomers and, combined with validated force fields, inform the rational design and modeling of conjugated polymers derived from these building blocks.

## Supplementary Material



## Data Availability

All data needed to evaluate the conclusions in the paper are present in the paper and/or the Supporting Information. Additional data can be accessed via Zenodo: 10.5281/zenodo.17867529.

## Notes

Experimental sections; computational methods; synthetic procedures; Figures S1–S56; and Tables S1–S17 (PDF)
