# Supplementary material for: From π–π Stacking to Chain Entanglements: Single Crystals of Oligoether-Substituted Thieno[3,2‑b]thiophenes
Source: Macromolecules. 2026 Mar 17;59(7):4612–21. doi: 10.1021/acs.macromol.6c00172 (PMC13085854; doi:10.1021/acs.macromol.6c00172)
Supplement: Supplementary file 1 [file ma6c00172_si_001.pdf]

Supporting information

From π-π Stacking to Chain Entanglements: Single Crystals of Oligoether-Substituted Thieno[3,2-*b*]thiophenes

Joost Kimpel,[1]* Iona Anderson,[2] Di Zhu,[1] Jyotsana Kala,[2] Przemyslaw Sowinski,[1] Alexander Giovannitti,[1] Lars Öhrström,[1] Jenny Nelson,[2] Christian Müller[1,3]*

[1] Department of Chemistry and Chemical Engineering, Chalmers University of Technology, Göteborg, Sweden

[2]Department of Physics, Imperial College London, London, UK

[3] Wallenberg Initiative Materials Science for Sustainability, Department of Chemistry and Chemical Engineering, Chalmers University of Technology, Göteborg, Sweden

* kimpel@chalmers.se; christian.muller@chalmers.se

Experimental Section.

Chemicals. Components necessary for small molecule synthesis, i.e. 3,6-dibromothieno[3,2-*b*]thiophene (Angene, 98 %), copper(I) iodide (Sigma Aldrich, ≥ 99.5 %), sodium hydride (Sigma Aldrich, 60 %, dispersion in mineral oil), and potassium *tert*-butoxide (TCI, > 97 %), were used as received. Sodium methoxide in methanol (Sigma Aldrich, 25 wt %), anhydrous 2-methoxyethanol (Thermo Scientific, > 99.0 %), and anhydrous 2-methyltetrahydrofuran (Thermo Scientific, ≥ 99.0 %) were taken from the received bottle under a constant stream of nitrogen. Diethylene glycol monomethyl ether (with butylated hydroxytoluene (BHT) as stabilizer, TCI, > 99 %), triethylene glycol monomethyl ether (Sigma Aldrich, 95 %), and tetraethylene glycol monomethyl ether (Ambeed, > 99 %) were dried over molecular sieves before use. Chemicals for work-up and purification, i.e. *n*-hexanes (Fisher Scientific, ≥ 99.5 %), ethyl acetate (Fisher Scientific, ≥ 99.5 %), chloroform (Fisher Scientific, ≥ 99.8 %), diethyl ether (Fisher Scientific, ≥ 99.5 %), anhydrous magnesium sulphate (Sigma Aldrich, ≥ 99.5 %), and silica (VWR, NORMASIL 60®, 40 – 63 µm), were used as received.

Nuclear Magnetic Resonance. Room temperature spectra were recorded on a Bruker Avance NEO 600 spectrometer (^{1}H: 600.13 MHz, ^{13}C: 150.90 MHz). The ^{1}H and ^{13}C NMR spectra were referenced to the residual solvent peak ($CDCl_3$: $\delta(^{1}H)$ = 7.26 ppm, $\delta(^{13}C)$ = 77.16 ppm).

Crystallization. A generalized protocol for the growth of crystals was utilized. All 3,6-bis(oligoethylene glycol monomethyl ether)thieno[3,2-*b*]thiophenes were grown by vapor diffusion crystallization in a cold environment. Chloroform was used as the solvent and diethyl ether as the anti-solvent. Circa 20 mg of compound was put in a 4 mL screw cap vial and dissolved using 2-3 drops of chloroform. The 4 mL screw cap vial was left open and placed inside a broad 20 mL screw cap vial. Using a pipette, 10 mL of diethyl ether was added to the 20 mL screw cap vial, taking extra care not to drop any diethyl ether inside the 4

mL screw cap vial with the compound. After addition of the diethyl ether, the cap was tightly screwed onto the 20 mL screw cap vial and the vial was placed inside a fridge at 7 °C for 24 to 48 hours. In case no crystallization had occurred upon vapor diffusion, the system was placed in the freezer at -20 °C for an additional 24 hours. After crystallization had occurred, the supernatant was removed, and crystals were washed thrice using cold (-20 °C) diethyl ether. Final crystals were kept in the freezer at -20 °C.

Single crystal X-ray diffraction. Suitable crystals were mounted on a nylon loop inside an XtaLAB Rigaku Synergy R, HyPix diffractometer. Diffractograms were recorded using CuKα radiation (λ = 1.54184 Å). The crystals were kept at a steady temperature, which ranged from T = 116.0 to 156.0 K during data collection. Their structures were solved with the ShelXT21 structure solution program using the Intrinsic Phasing solution method and by using Olex2 and Mercury as the graphical interface.[1, 2]

Thermal analysis. Differential scanning calorimetry (DSC) thermograms were recorded with a Mettler Toledo DSC2 equipped with a gas controller GC 200 system. Samples obtained from crystallization were heated/cooled between -40 °C to 160 °C for three consecutive cycles under nitrogen atmosphere (flow rate = 60 mL min^{-1}) using a heating/cooling rate of 10 °C min^{-1}. Each measurement started/ended at 25 °C. Melt crystallization in a DSC aluminum pan was performed for 3,6-bis(triethylene glycol monomethyl ether)thieno[3,2-*b*]thiophene (g_3TT) and 3,6-bis(tetraethylene glycol monomethyl ether)thieno[3,2-*b*]thiophene (g_4TT). This was achieved by cold crystallization: samples obtained from vapor diffusion crystallization were heated to 160 °C and cooled to -40 °C at a rate of 10 °C min^{-1}. Then, the sample was heated to 20 °C (first T_m polymorph for both g_3TT and g_4TT), 45 °C (second T_m polymorph g_3TT) or 55 °C (second T_m polymorph g_4TT) and kept at this temperature for one hour. Completion of crystallization was confirmed by lack of exothermic heat flow.

Computational Methods.

Force field optimizations. Our force field is based on the OPLS-AA force field which has been reparametrized to reproduce stable crystal structures derived from experimental SC-XRD.[3] The focus of our force field development is the partial charges attributed to each atom type and the torsional potential associated with the rotation of the side chains. We parameterize the force field to fit values derived from density functional theory (DFT) at the B3LYP/6-311G** level of theory, the quantum calculation software we used was Gaussian 16.[4] This level of theory was chosen to maintain consistency across previous work that has been done on our force field by N. Siemons, however comparisons were also made to the ωB97X-D functional and MP2 where similar trends in partial charges were observed and there were only slight deviations in the torsional potential.[5] Glycol side chain parameters are primarily taken from work by D. Pearce and are based on OPLS-AA atom types, with other bonded parameters from work by M. Moreno *et. al.* and R. Bhatta *et. al.*[6, 7] We calculated the partial charges for each atom type through DFT on an experimentally resolved unit cell for each monomer variant using the Chelpg scheme for atomic charge fitting (Tables S7-S12).[8] Partial charges were then averaged across like atom types in the unit cell and scaled such that the total partial charge on the thieno[3,2-*b*]thiophene core and the total partial charge on the glycol side chains were mutually equal to zero. This approach facilitates increased generalisability of the force field as the same parameters for the thieno[3,2-*b*]thiophene core can be maintained between monomer variants. Additionally, the partial charges from the unit cell conformations were compared to the optimized geometry for a g_0TT monomer and previously defined ethylene glycol partial charge parameters to ensure generalizability and avoid overfitting to a specific crystal configuration.[9]

In order to parameterize torsional potentials, the system must be reduced to just the atoms most relevant to the dihedral that requires parameterisation. This is because further degrees of freedom can complicate energy landscapes through conformational effects, making it difficult to isolate the torsional potential. In this case, the g_0TT structure was selected to find generalized parameters for all monomer variants. Since long side chains have been removed the only degree of freedom to be considered is the torsional potential associated with the rotation of the methyl unit around the oxygen. The parameters associated with the dihedral torsion, modelled here through a Ryckyaert-Belleman function, are set to zero while all other force field parameters are maintained as normal. We then perform a molecular dynamics (MD) scan by fixing the dihedral angle at 5° intervals and performing an energy minimisation for each fixed conformer. The scan is carried out over 360° to give the potential energy surface associated with the system excluding the torsional degree of freedom, this can be considered a background potential. A similar scan is carried out on an equivalent system using DFT, both scans are carried out in vacuum (Figure S38). In the DFT scan, the torsional potential is implicitly included within the total potential energy surface, therefore the difference between the potential derived from DFT and the background potential should be equivalent to the torsional potential of the dihedral. To obtain the parameters required for the force field, we fit the difference between the two potentials to the Ryckyaert-Belleman function, which returns five coefficients (Table S13). Once parameters have been determined, supercells of these crystals are then simulated in MD to test for stability within our force field. If the new parameters result in unstable crystal configurations, the force field is adjusted until stable crystals can be successfully reproduced. Additionally, comparison between simulated and experimental XRD spectra is used to confirm force field validity.

Molecular dynamics procedure. To validate our force field, we performed molecular dynamics simulations using GROMACS 2022.4.[10, 11] Initial crystal configurations were prepared from the experimentally resolved crystal structures. The initial unit cell, as defined in .cif file format, was expanded to a supercell using the GROMACS command-line tool 'gmx genconf'. Each supercell consisted of 5x5x5 unit cells, or 250 monomers. Initial simulations were conducted at 298 K and consisted of an energy minimisation step followed by a 2 ns NVT equilibration and a 40 ns NPT production run. The energy minimisation step utilized steepest decent with the convergence criteria set to a maximal force of less than 10 kJ mol^{-1} nm^{-1}. In the NVT run, we applied a 2 fs timestep whilst the production run required a 0.5 fs timestep. The leap-frog algorithm was applied in both the NVT and NPT steps to integrate the equations of motion. The Verlet cut-off scheme is used for neighbor searching and the electrostatics and Van der Waals forces are calculated through particle mesh Ewald summation (PME) with a 1 nm cutoff distance.[12] The LINCS algorithm was used to constrain hydrogen bonds. Temperature coupling was carried out using a velocity rescale algorithm which includes a stochastic term to ensure a proper canonical ensemble is generated.[13] The NPT production run used Berendsen pressure coupling.[14]

We applied an identical method to establish the temperature dependence of crystal stability under our force field for six different temperatures. Starting at 93 K the temperature was increased in 50 K increments to span both the temperature range of the differential scanning calorimetry and the temperatures at which XRD measurements are taken. At each temperature the procedure outlined above was repeated. We used the Mercury software to calculate simulated XRD spectra for comparison to the spectra extracted from the single crystals through Mercury software.[2]

Synthetic Procedures.

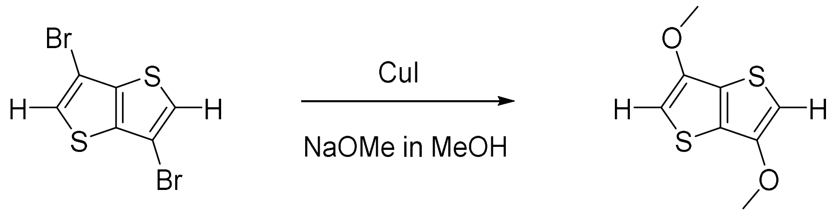

Synthesis of 3,6-bis(methoxy)thieno[3,2-b]thiophene. 3,6-Dibromothieno[3,2-*b*]thiophene (1.00 g, 3.36 mmol, 1.00 eq) and copper(I) iodide (0.640 g, 3.36 mmol, 1.00 eq) were charged in a 20 mL high-pressure reaction vial and brought under inert atmosphere. The vial was moved into an ice bath and, under a constant nitrogen flow, degassed 25 wt% sodium methoxide in methanol (6.00 mL, 26.9 mmol NaOMe, 8.00 eq) was added in two parts while gently stirring the solution. The high-pressure reaction vial was transferred to an aluminum heating block and heated to 90 °C under vigorous stirring. The reaction was monitored throughout by TLC using ethyl acetate/*n*-hexanes = 1/19 as the eluent (Retention factor $R_{f,prod}$ = 0.35). After 48 hours, the vial was removed from the heating block and cooled down to room temperature. The solution was poured into 100 mL 1M HCl solution and transferred to a separatory funnel. The aqueous phase was extracted three times with 100 mL chloroform. All organic layers were collected and subsequently washed with water three times. The organic phase was collected, dried using anhydrous magnesium sulphate and concentrated *in vacuo* to obtain a gray solid. The solid was redissolved in ethyl acetate and passed through an isocratic silica column with ethyl acetate/*n*-hexanes = 1/19 as the eluent. The product fraction was collected and concentrated to yield grey crystals (0.378 g, 1.89 mmol, 56 %). ^{1}H NMR ($CDCl_3$, 600 MHz, δ ppm): 6.26 (s, 2H), 3.92 (s, 6H); ^{13}C NMR ($CDCl_3$, 151 MHz, δ ppm): 151.0, 128.6, 97.5, 57.7; Isolation was also confirmed by single crystal X-ray structure (CCDC number: 2503697).

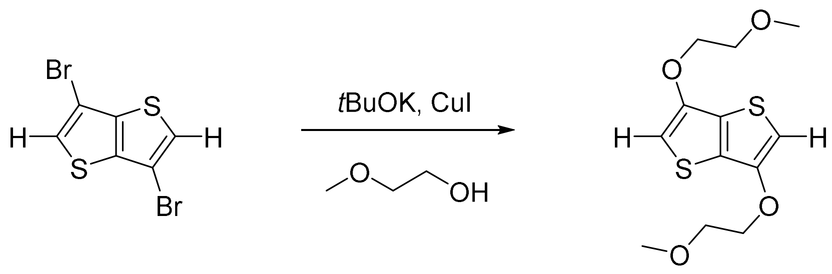

Synthesis of 3,6-bis(monoethylene glycol monomethyl ether)thieno[3,2-b]thiophene. Anhydrous and degassed 2-methoxyethanol (10.0 mL) was charged into a two-necked 50 mL round bottom flask equipped with a condenser under an inert atmosphere. To the round bottom flask, potassium *tert*-butoxide (1.51 g, 13.4 mmol, 4.00 eq) was added in three parts over 10 minutes followed by 1 hour of vigorous stirring at 100 °C. Then, 3,6-dibromothieno[3,2-*b*]thiophene (1.00 g, 3.36 mmol, 1.00 eq) and copper(I) iodide (0.640 g, 3.36 mmol, 1.00 eq) were added to the solution after which the reaction was left at 100 °C under vigorous stirring. The reaction was monitored throughout with thin layer chromatography using ethyl acetate/*n*-hexanes = 1/3 as the eluent (Retention factor $R_{f,prod}$ = 0.40). After 24 hours, the reaction mixture was cooled down to room temperature. The solution was poured into 100 mL 1M HCl solution and transferred to a separatory funnel. The aqueous phase was extracted three times with 100 mL chloroform. All organic layers were collected and subsequently washed with water three times. The organic phase was collected, dried using anhydrous magnesium sulphate and concentrated *in vacuo* to obtain a brown solid. The solid was redissolved in ethyl acetate and passed through a silica column with a gradient of ethyl acetate/*n*-hexanes = 1/5 to ethyl acetate/*n*-hexanes = 1/1 as the eluent. The product fraction was collected and concentrated to yield an off-white powder (0.352 g, 1.22 mmol, 36 %). ^{1}H NMR ($CDCl_3$, 600 MHz, δ ppm): 6.29 (s, 2H), 4.22 (t, J = 4.9 Hz, 4H), 3.77 (t, J = 4.7 Hz, 4H), 3.45 (s, 6H); ^{13}C NMR ($CDCl_3$, 151 MHz, δ ppm): 149.9, 128.8, 98.8, 71.0, 70.0, 59.4; Isolation was also confirmed by single crystal X-ray structure (CCDC number: 2503699).

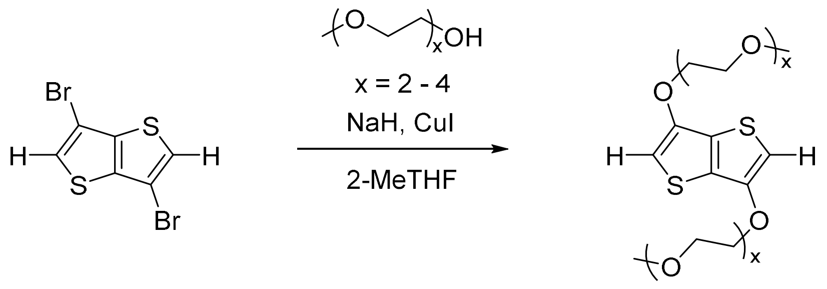

General synthesis of longer 3,6-bis(oligoethylene glycol monomethyl ether)thieno[3,2-b]thiophenes. The synthesis of thieno[3,2-*b*]thiophene with oligoethylene glycol monomethyl ether chains of length 2 to 4 were synthesized with a similar synthetic protocol.[15, 16] Specifics are annotated for each case. Anhydrous 2-methyltetrahydrofuran (50 mL) was charged into a pre-dried two-necked 250 mL round bottom flask under nitrogen atmosphere equipped with a condenser. The system was cooled down using an ice bath. Whilst stirring the solution, 60 % mineral suspension of sodium hydride (0.336 g, 8.40 mmol, 2.50 eq) was added, and the dispersion was brought back under an inert atmosphere. The oligoethylene glycol monomethyl ether (10.1 mmol, 3.00 eq) was added dropwise to the reaction using a syringe. The mixture was left to stir for at least 30 minutes at room temperature. Once effervescence had stopped, solids of 3,6-dibromothieno[3,2-*b*]thiophene (1.00 g, 3.36 mmol, 1.00 eq) and copper(I) iodide (0.320 mg, 1.68 mmol, 0.500 eq) were mixed and added to the solution simultaneously. The reaction mixture was brought back under an inert atmosphere and heated by 15 °C every 10 minutes until a final temperature of 100 °C was reached. The reaction mixture was left refluxing under vigorous stirring overnight. The reaction was checked for completion by TLC (eluent system specified for each compound). The reaction mixture was then cooled down to room temperature and diluted with 150 mL chloroform. The mixture was transferred to a separatory funnel and washed with 3 × 100 mL water. The combined aqueous layer was then extracted with 100 mL chloroform and the organic phases were combined and dried over anhydrous magnesium sulphate. The mixture was concentrated *in vacuo*.

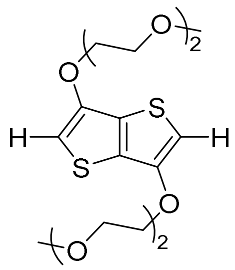

Synthesis of 3,6-bis(diethylene glycol monomethyl ether)thieno[3,2-b]thiophene. For monitoring the reaction, the eluent system used was ethyl acetate/*n*-hexanes = 1/1 (Retention factor $R_{f,prod}$ = 0.25). The obtained brown solid was redissolved in ethyl acetate and passed through an isocratic silica column with ethyl acetate/*n*-hexane = 1/1 as the eluent. The product fraction was collected and concentrated to yield a pale-yellow powder (0.657 g, 1.75 mmol, 52%). ^{1}H NMR ($CDCl_3$, 600 MHz, δ ppm): 6.29 (s, 2H), 4.22 (t, *J* = 4.9 Hz, 4H), 3.88 (t, *J* = 4.9 Hz, 4H), 3.73 (t, *J* = 4.7 Hz, 4H), 3.58 (t, *J* = 4.9 Hz, 4H), 3.39 (s, 6H); ^{13}C NMR ($CDCl_3$, 151 MHz, δ ppm): 149.9, 128.7, 98.7, 72.1, 71.0, 70.1, 69.7, 59.2; Isolation was also confirmed by single crystal X-ray structure (CCDC number: 2503698).

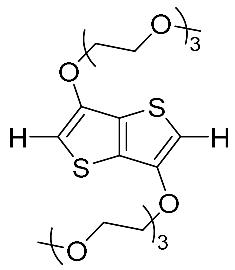

Synthesis of 3,6-bis(triethylene glycol monomethyl ether)thieno[3,2-b]thiophene. Synthesis and characterization by NMR and single crystal X-ray was described previously.[15, 16] Isolation was also confirmed by single crystal X-ray structure (CCDC number: 2336289).[15]

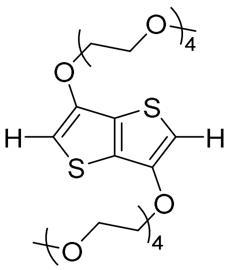

Synthesis of 3,6-bis(tetraethylene glycol monomethyl ether)thieno[3,2-b]thiophene. For monitoring the reaction, the eluent system used was ethyl acetate/acetone = 19/1 (Retention factor $R_{f,prod}$ = 0.20). The obtained brown oil was passed through a silica column using a gradient of pure ethyl acetate to ethyl acetate/acetone = 19/1 as the eluent. The obtained yellow oil was dispersed in diethyl ether and recrystallized by placing into the freezer at -20 °C to yield yellow crystals (0.634 g, 1.15 mmol, 34 %). ^{1}H NMR ($CDCl_3$, 600 MHz, δ ppm): 6.28 (s, 2H), 4.22 (t, J = 5.1 Hz, 4H), 3.87 (t, J = 5.1 Hz, 4H), 3.75-3.70 (m, 4H), 3.69-3.62 (m, 16H), 3.55-3.51 (m, 4H), 3.36 (s, 6H); ^{13}C NMR ($CDCl_3$, 151 MHz, δ ppm): 149.9, 128.7, 98.7, 72.1, 71.0, 70.8 (multiple), 70.6 (multiple), 70.1, 69.7, 59.2; Isolation was also confirmed by single crystal X-ray structure (CCDC number: 2503700 and 2504797).

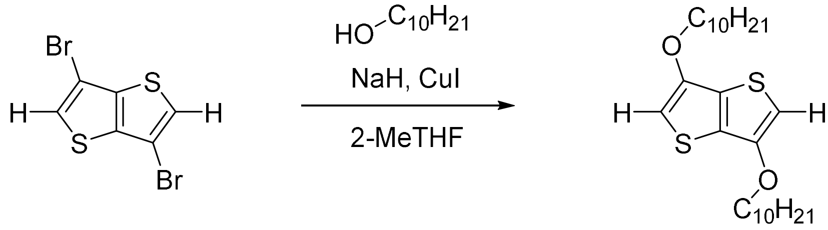

Synthesis of 3,6-bis(decyloxy)thieno[3,2-b]thiophene. Synthesis and characterization by NMR was reported previously.[17] Crystallization was done by dissolving aTT in a small amount of *n*-hexanes at room temperature followed by cooling to 7 °C for 24 h to provide single crystals analyzed by single crystal X-ray diffraction (CCDC number: 2503701).

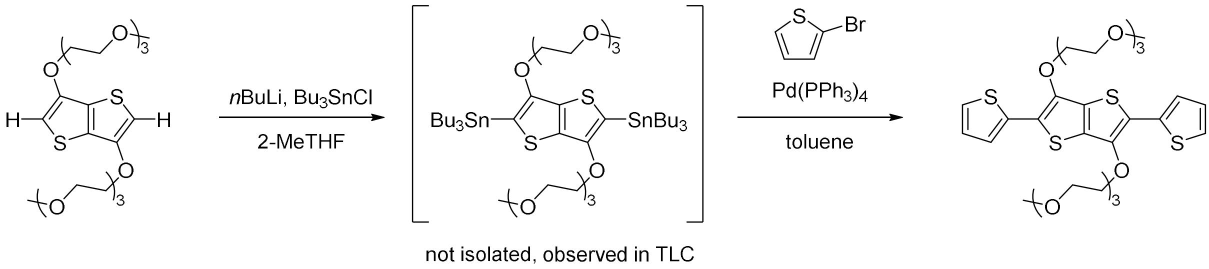

Synthesis of 3,6-bis(triethylene glycol monomethyl ether)-2,5-di(thiophen-2-yl)thieno[3,2-b]thiophene. 3,6-Bis(triethylene glycol monomethyl ether)thieno[3,2-*b*]thiophene (0.250 g, 0.522 mmol, 1.00 eq) was charged in a pre-dried two-necked 25 mL round bottom flask under nitrogen atmosphere. This was dissolved in anhydrous 2-methyltetrahydrofuran (5 mL), which was then cooled to -78 °C using an acetone/dry ice bath. To the solution, *n*-BuLi in hexanes (2.5 M, 0.627 mL, 1.57 mmol, 3.00 eq) was added dropwise. Once all *n*-BuLi was added, the mixture was stirred at -78 °C for 1 h. Tributyltin choride (0.425 mL, 1.57 mol, 3.00 eq) was added to the reaction mixture dropwise, accompanied with an instantaneous color change from yellow to orange. The resulting mixture was stirred at -78 °C for 30 min, warmed to room temperature, and stirred further for 1 h. After formation of two distinct new spots on TLC, using ethyl acetate/hexane = 1/1 as the eluent, the solvent was removed from the reaction mixture *in vacuo but without heating*. Anhydrous toluene (5 mL) was then added to the mixture, followed by agitation and sonication. The resulting solution, containing the stannylated intermediate, was filtered into a new pre-dried two-necked 25 mL round bottom flask through qualitative filter paper. Tetrakis(triphenylphosphine)palladium (0.030 g, 0.026 mmol, 0.05 eq) and 2-bromothiophene (0.152 mL, 1.57 mol, 3.00 eq) were added to the round bottom flask, after which it was equipped with a condenser and brought under nitrogen atmosphere. Under stirring, the reaction mixture was heated to 105 °C for 12 hours. The mixture was poured into saturated aqueous Na_2CO_3 solution and extracted with dichloromethane thrice. All organic layers were collected and subsequently washed with water thrice. The organic phase was collected, dried using anhydrous magnesium sulphate

and concentrated *in vacuo* to obtain a dark orange oil. The obtained oil was passed through a silica column using a gradient of ethyl acetate/*n*-hexanes = 1/1 to pure ethyl acetate as the eluent (Retention factor in ethyl acetate/*n*-hexanes = 1/1 $R_{f,prod}$ = 0.20). The material was obtained as an orange oil (0.087 g, 0.136 mmol, 26 %). ^{1}H NMR ($CDCl_3$, 600 MHz, δ ppm): 7.30-7.25 (m, 4H), 7.03 (dd, *J* = 5.3, 3.7 Hz, 2H), 4.46 (t, *J* = 4.9 Hz, 4H), 3.90 (t, *J* = 4.8 Hz, 4H), 3.77-3.71 (m, 4H), 3.71-3.67 (m, 12H), 3.56-3.52 (m, 4H), 3.37 (s, 6H); ^{13}C NMR ($CDCl_3$, 151 MHz, δ ppm): 144.7, 134.8, 127.1, 126.1, 124.9, 123.7, 120.6, 72.1, 71.6, 71.1, 70.9, 70.8, 70.2, 59.2; Isolation was also confirmed by single crystal X-ray structure (CCDC number: 2504799).

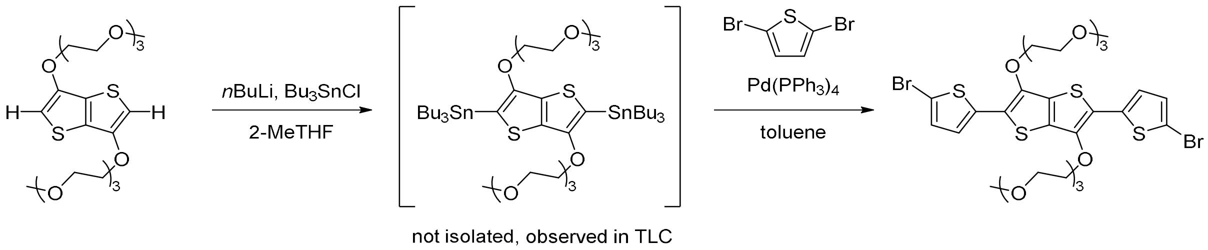

Synthesis of 2,5-bis(5-bromothiophen-2-yl)-3,6-bis(triethylene glycol monomethyl ether)thieno[3,2-b]thiophene. All steps and amounts before addition of the palladium catalyst and 2-bromothiophene from the synthesis of 3,6-bis(triethylene glycol monomethyl ether)-2,5-di(thiophen-2-yl)thieno[3,2-*b*]thiophene (i.e. formation of stannylated intermediate) were repeated. Then, tetrakis(triphenylphosphine)palladium (0.030 g, 0.026 mmol, 0.05 eq) and 2,5-dibromothiophene (0.177 mL, 1.57 mol, 3.00 eq) were added to the round bottom flask containing the filtered toluene solution with the stannylated intermediate, after which it was equipped with a condenser and brought under nitrogen atmosphere. Under stirring, the reaction mixture was heated to 105 °C for 12 hours. The mixture was poured into saturated aqueous Na_2CO_3 solution and extracted with dichloromethane thrice. All the organic layers

were collected and subsequently washed with water thrice. The organic phase was collected, dried using anhydrous magnesium sulphate and concentrated *in vacuo* to obtain a dark orange oil. The obtained oil was passed through a silica column using a gradient of ethyl acetate/*n*-hexane = 1/1 to pure ethyl acetate as the eluent (Retention factor in ethyl acetate/*n*-hexanes = 1/1 $R_{f,prod}$ = 0.35, only visible with long wavelength). The material was obtained as an orange oil (0.096 g, 0.120 mmol, 23 %, 68 % pure by mass determined by NMR given presence of 3,6-bis(triethylene glycol monomethyl ether)thieno[3,2-*b*]thiophene). ^{1}H NMR ($CDCl_3$, 600 MHz, δ ppm): 6.96 (dd, J = 8.4, 3.9 Hz, 4H), 4.45 (t, J = 4.9 Hz, 4H), 3.90-3.86 (m, 4H), 3.76-3.50 (m, 16H), 3.37 (s, 6H); ^{13}C NMR ($CDCl_3$, 151 MHz, δ ppm): 144.9, 136.3, 129.7, 125.8, 123.2, 120.1, 112.1, 72.1, 71.7, 71.1, 70.8, 70.1, 69.7, 59.2; The pure compound was isolated by crystallization which was confirmed by single crystal X-ray structure (CCDC number: 2504798).

Supplementary Figures and Tables.

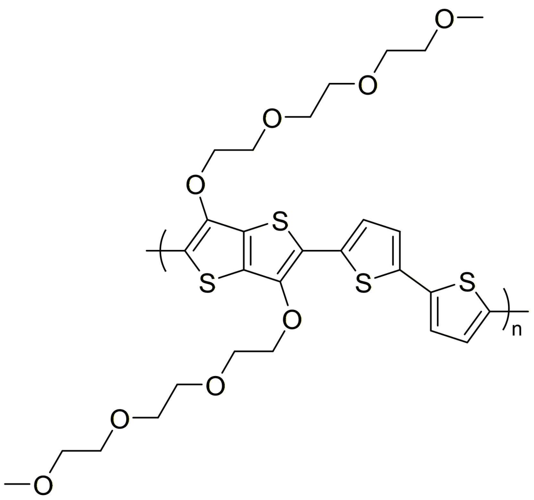

Figure S1. Chemical structure of poly(2-([2,2'-bithiophen]-5-yl)-3,6-bis(2-(2-(2-methoxyethoxy)ethoxy)ethoxy)thieno[3,2-b]thiophene) (p(g_3TT-T2)).

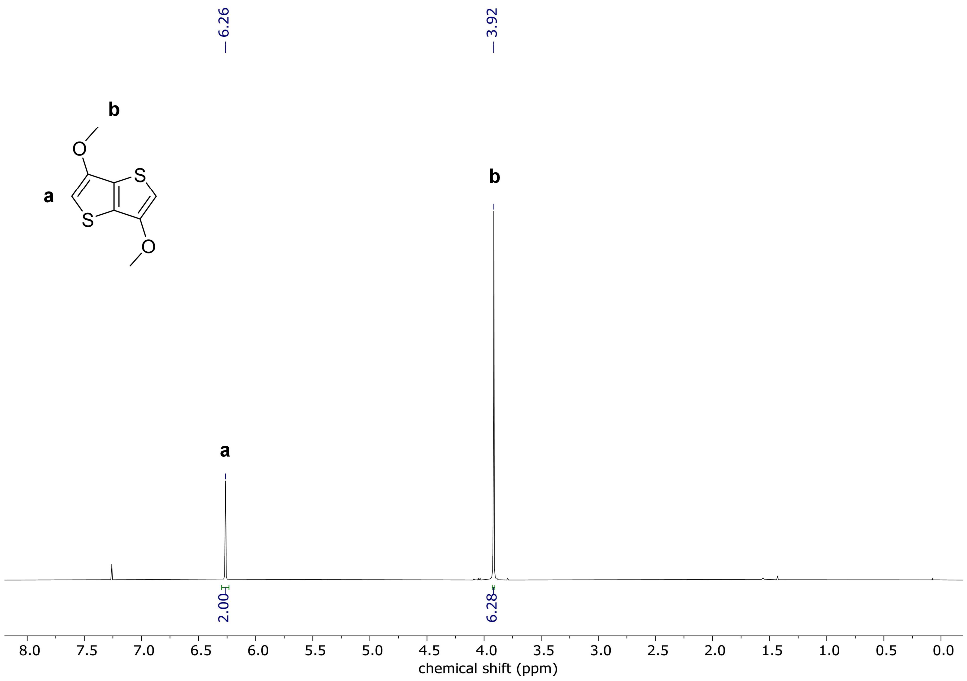

Figure S2. ^{1}H NMR spectrum ($CDCl_3$, 600 MHz, 298 K) of 3,6-dimethoxythieno[3,2-*b*]thiophene (g_0TT).

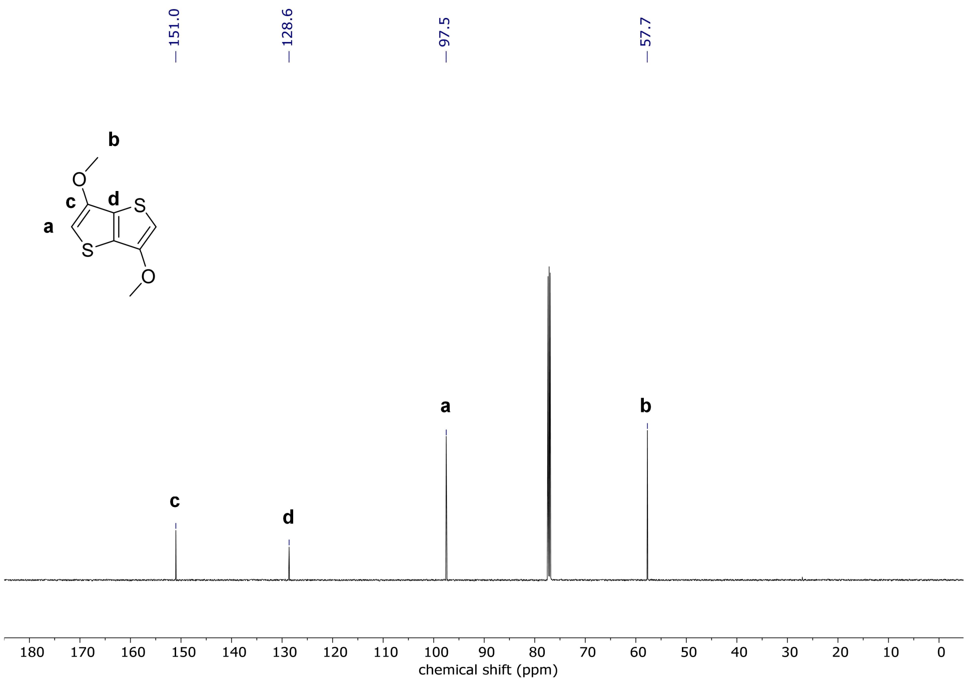

Figure S3. ^{13}C NMR spectrum ($CDCl_3$, 151 MHz, 298 K) of 3,6-dimethoxythieno[3,2-*b*]thiophene (g_0TT).

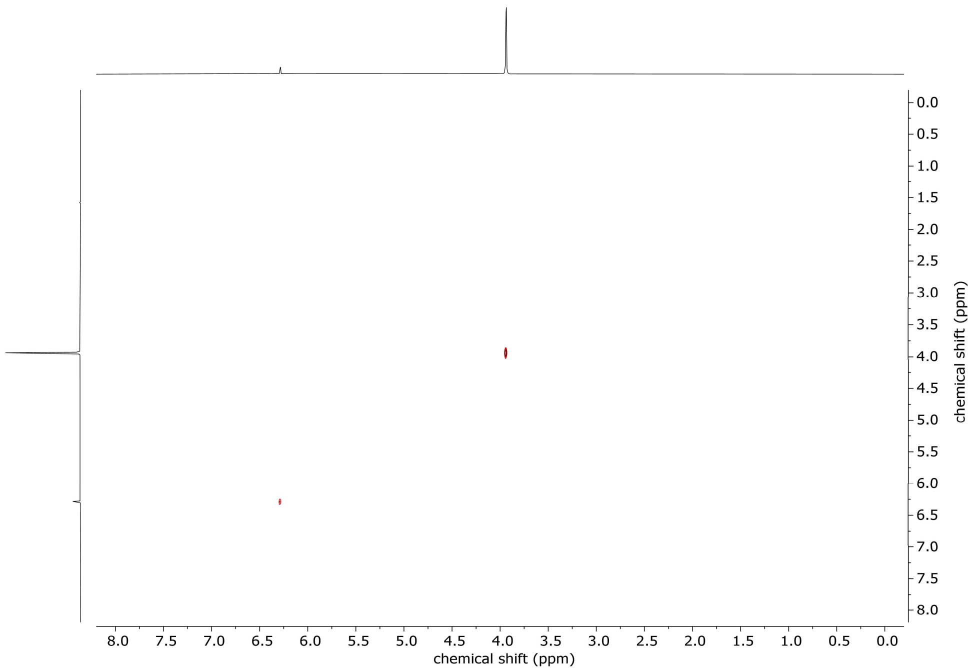

Figure S4. ^{1}H COSY NMR spectrum ($CDCl_3$, 600 MHz, 298 K) of 3,6-dimethoxythieno[3,2-*b*]thiophene (g_0TT).

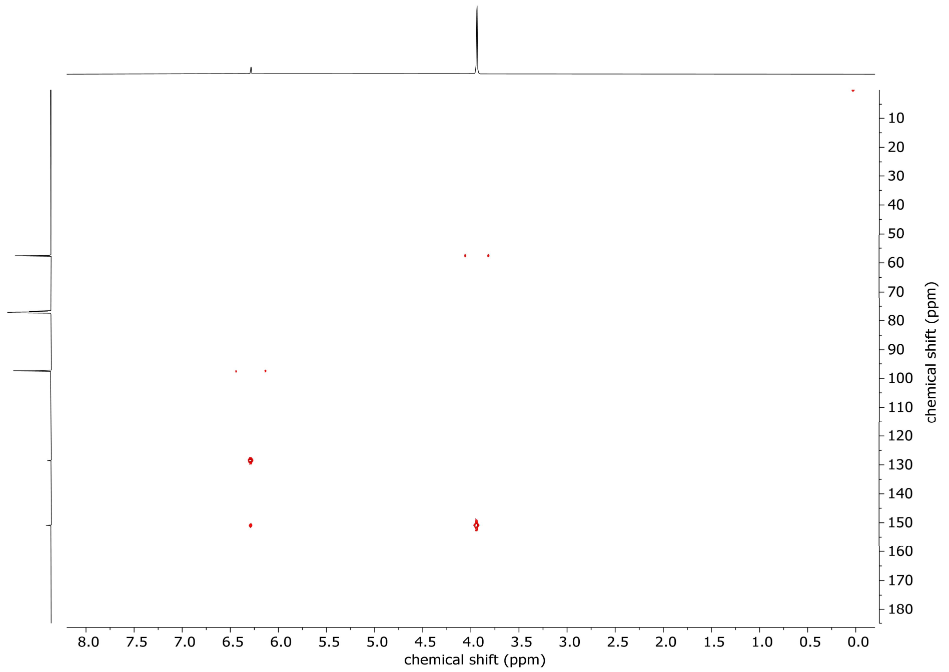

Figure S5. HMBC NMR spectrum ($CDCl_3$, 600/151 MHz, 298 K) of 3,6-dimethoxythieno[3,2-*b*]thiophene (g_0TT).

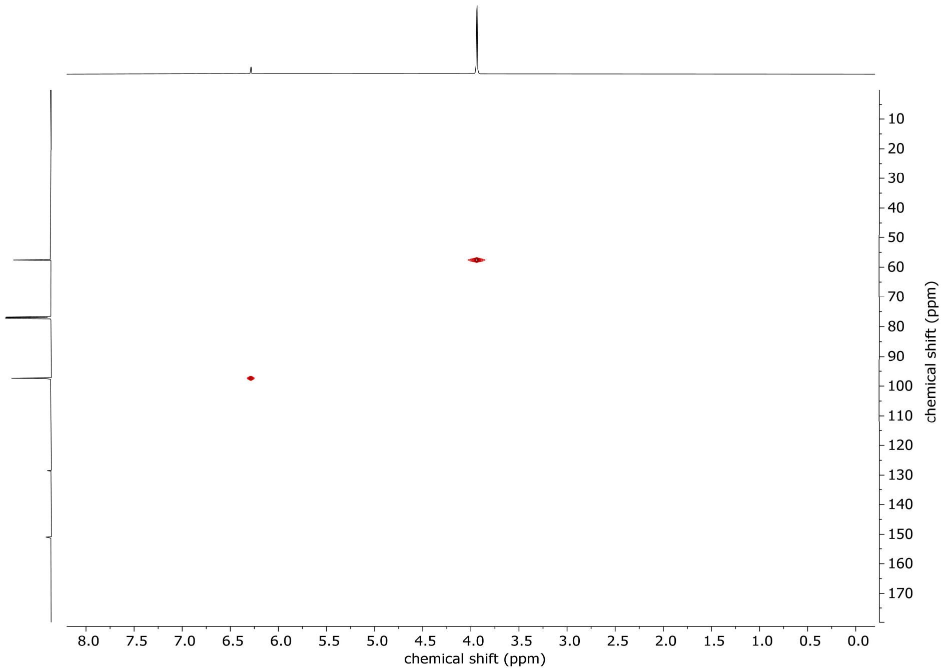

Figure S6. HSQC NMR spectrum ($CDCl_3$, 600/151 MHz, 298 K) of 3,6-dimethoxythieno[3,2-*b*]thiophene (g_0TT).

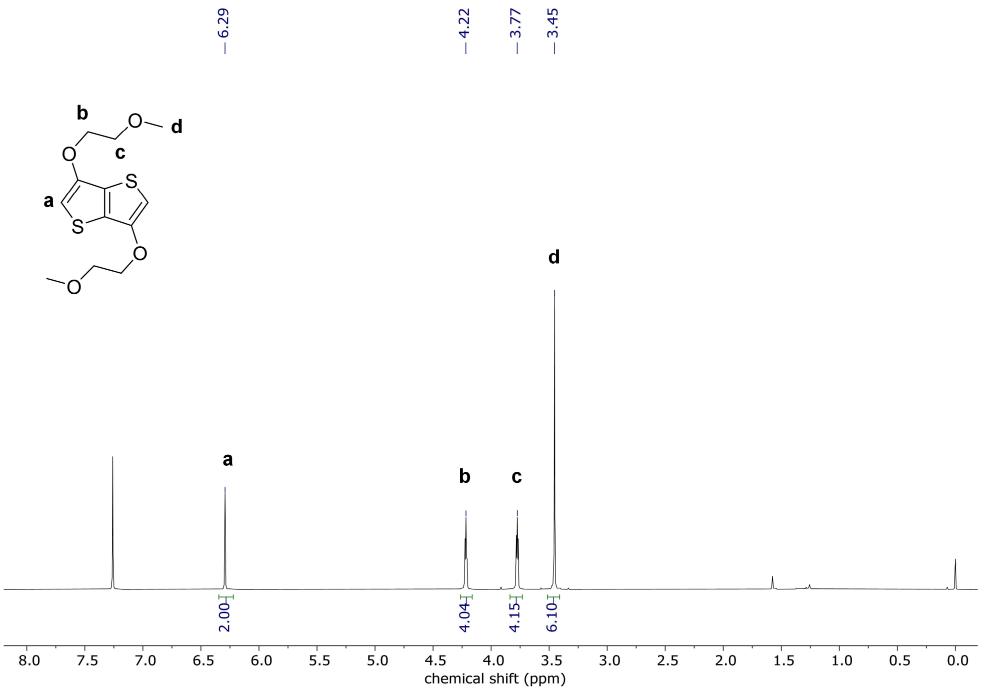

Figure S7. ^{1}H NMR spectrum ($CDCl_3$, 600 MHz, 298 K) of 3,6-bis(monoethylene glycol monomethyl ether)thieno[3,2-*b*]thiophene (g_1TT).

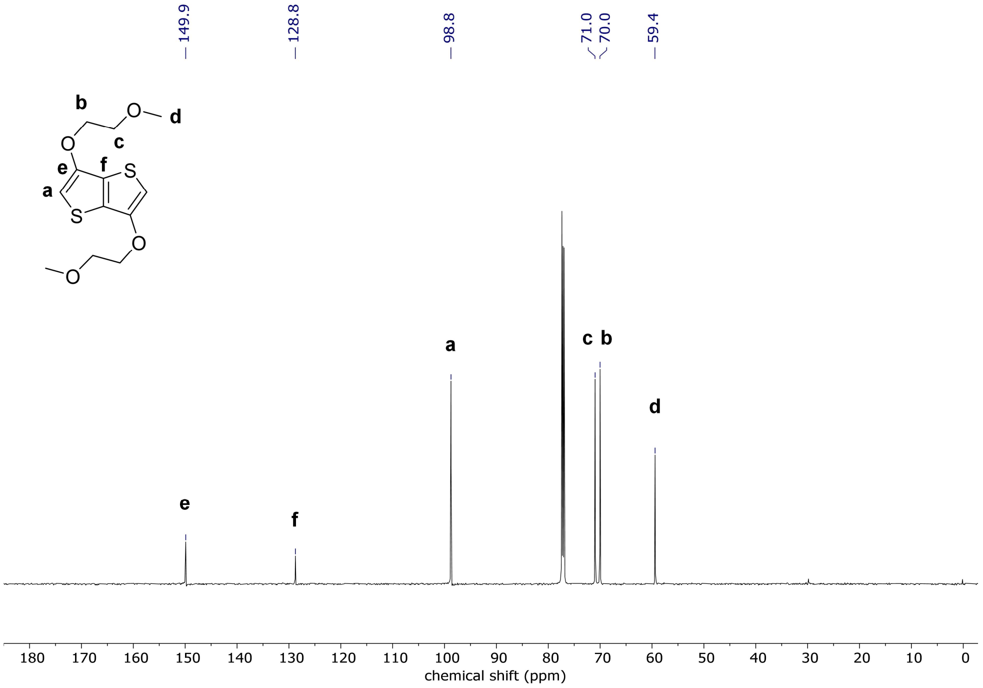

Figure S8. ^{13}C NMR spectrum ($CDCl_3$, 151 MHz, 298 K) of 3,6-bis(monoethylene glycol monomethyl ether)thieno[3,2-*b*]thiophene (g_1TT).

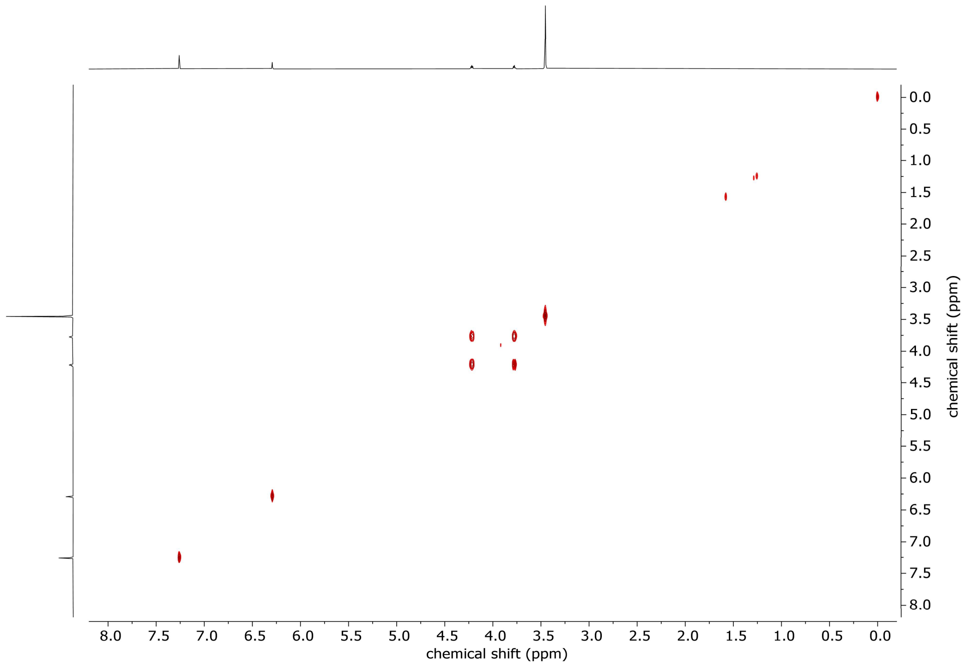

Figure S9. ^{1}H COSY NMR spectrum ($CDCl_3$, 600 MHz, 298 K) of 3,6-bis(monoethylene glycol monomethyl ether)thieno[3,2-*b*]thiophene (g_1TT).

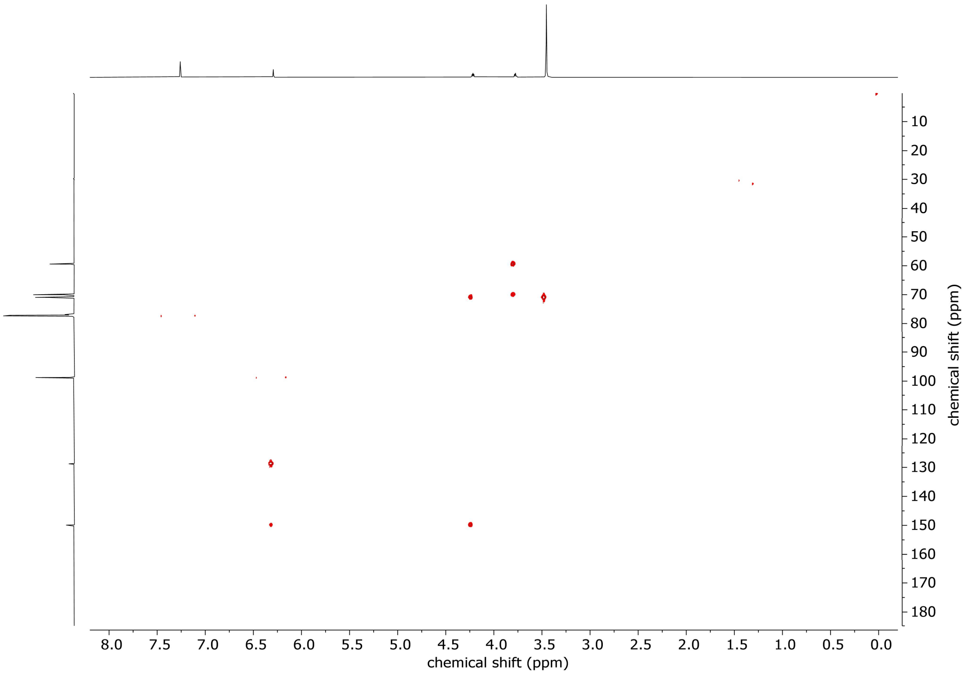

Figure S10. HMBC NMR spectrum ($CDCl_3$, 600/151 MHz, 298 K) of 3,6-bis(monoethylene glycol monomethyl ether)thieno[3,2-*b*]thiophene (g_1TT).

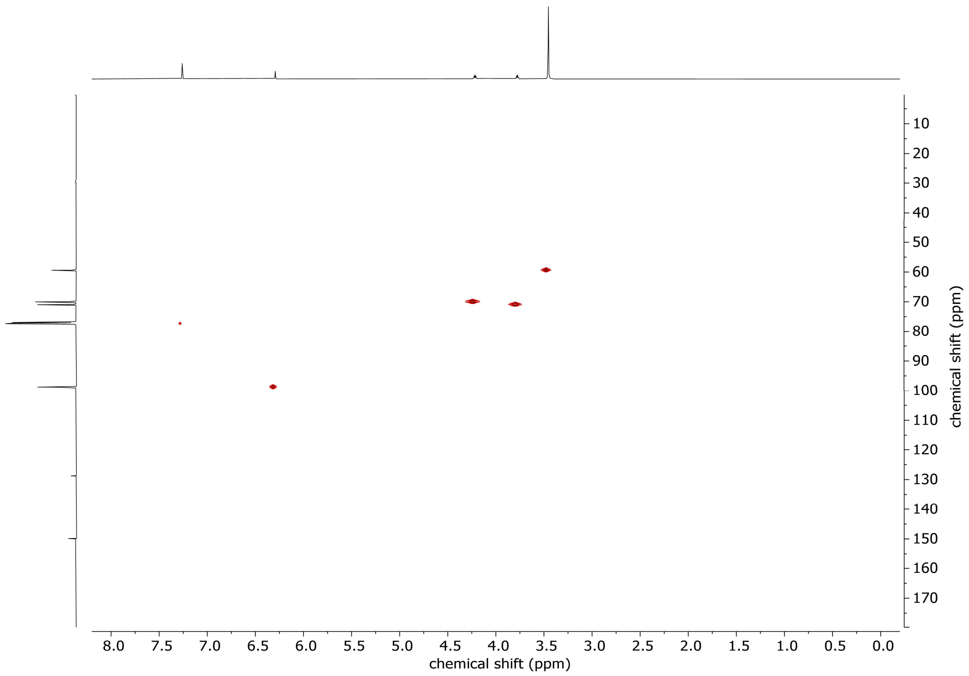

Figure S11. HSQC NMR spectrum ($CDCl_3$, 600/151 MHz, 298 K) of 3,6-bis(monoethylene glycol monomethyl ether)thieno[3,2-*b*]thiophene (g_1TT).

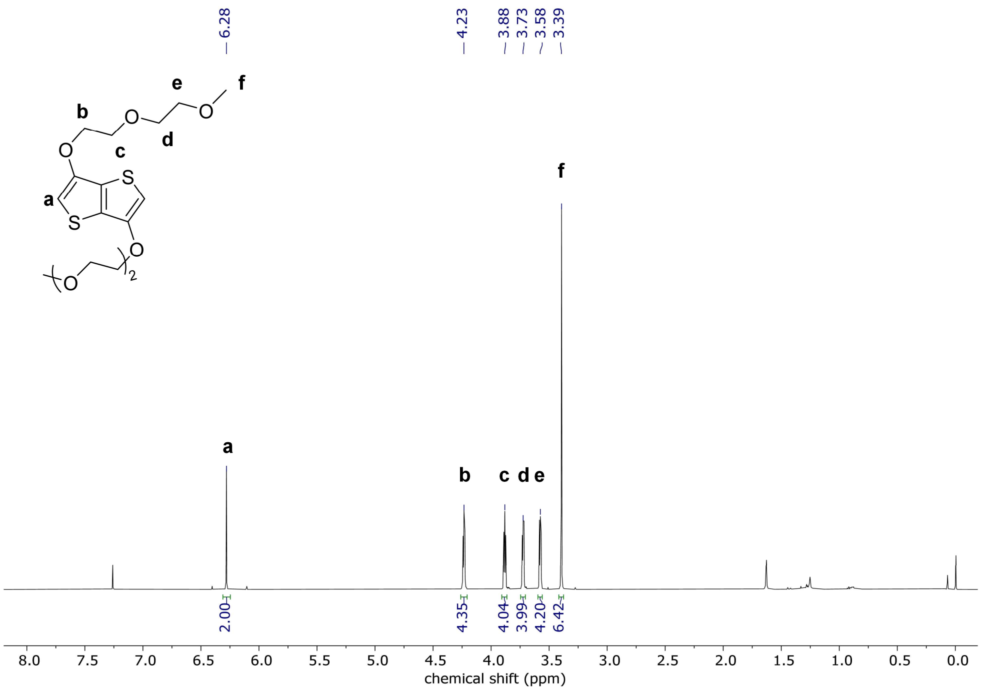

Figure S12. ^{1}H NMR spectrum ($CDCl_3$, 600 MHz, 298 K) of 3,6-bis(diethylene glycol monomethyl ether)thieno[3,2-*b*]thiophene (g_2TT).

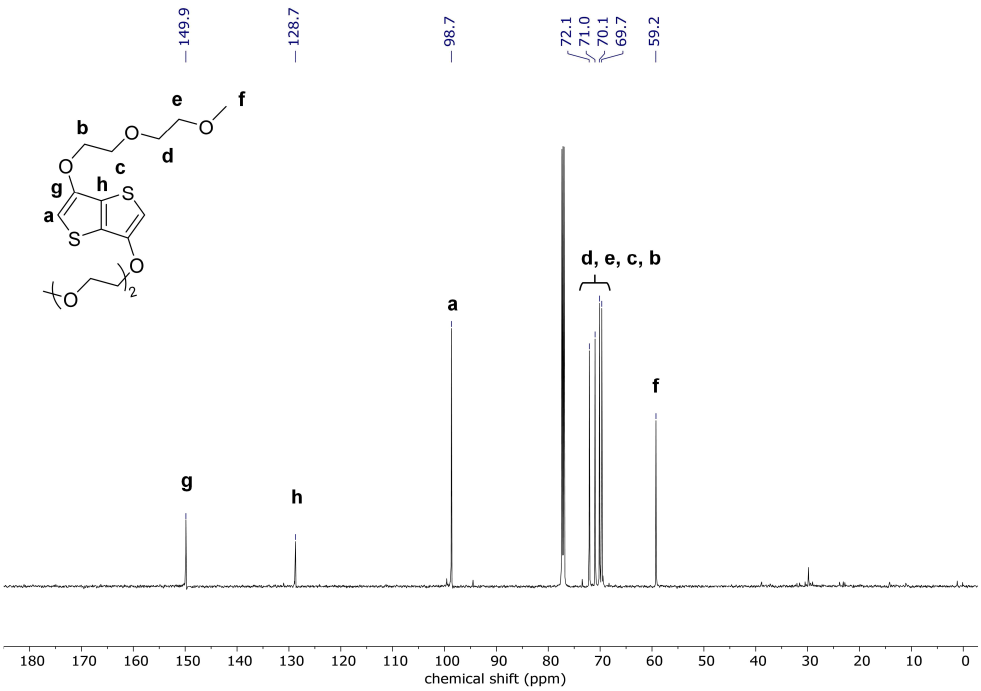

Figure S13. ^{13}C NMR spectrum ($CDCl_3$, 151 MHz, 298 K) of 3,6-bis(diethylene glycol monomethyl ether)thieno[3,2-*b*]thiophene (g_2TT).

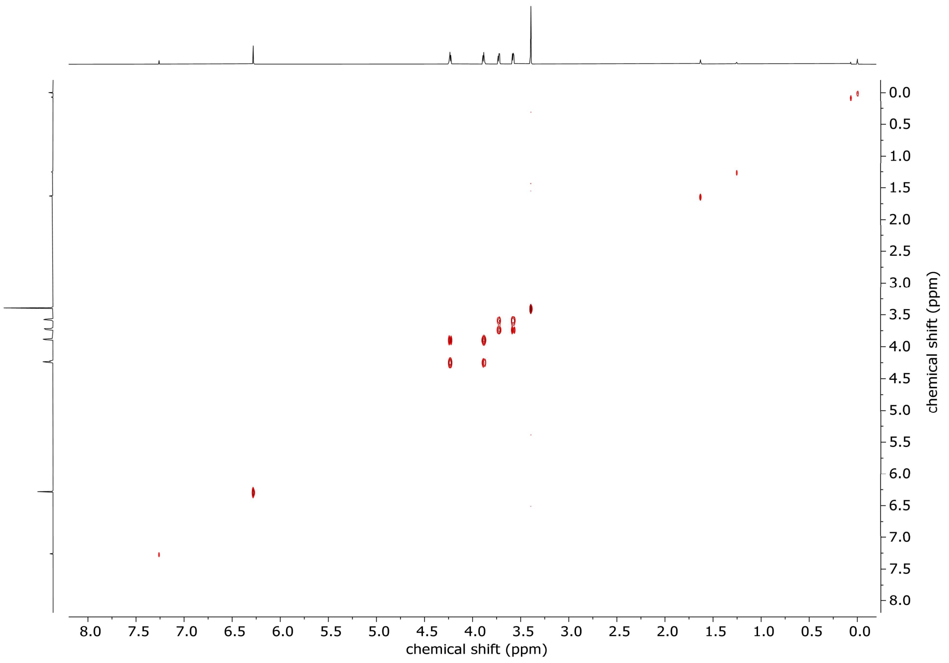

Figure S14. ^{1}H COSY NMR spectrum ($CDCl_3$, 600 MHz, 298 K) of 3,6-bis(diethylene glycol monomethyl ether)thieno[3,2-*b*]thiophene (g_2TT).

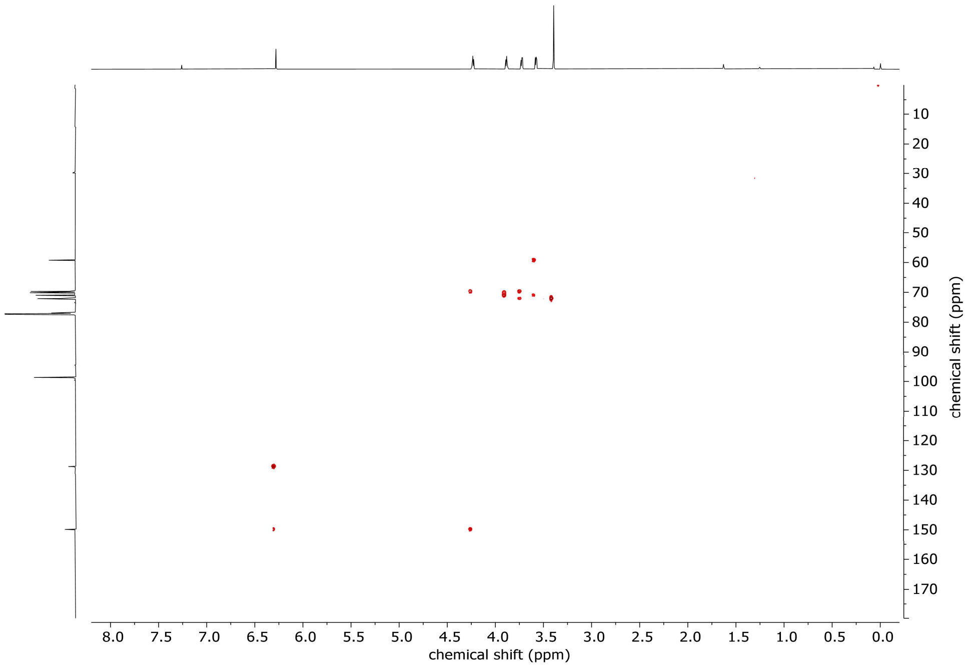

Figure S15. HMBC NMR spectrum ($CDCl_3$, 600/151 MHz, 298 K) of 3,6-bis(diethylene glycol monomethyl ether)thieno[3,2-*b*]thiophene (g_2TT).

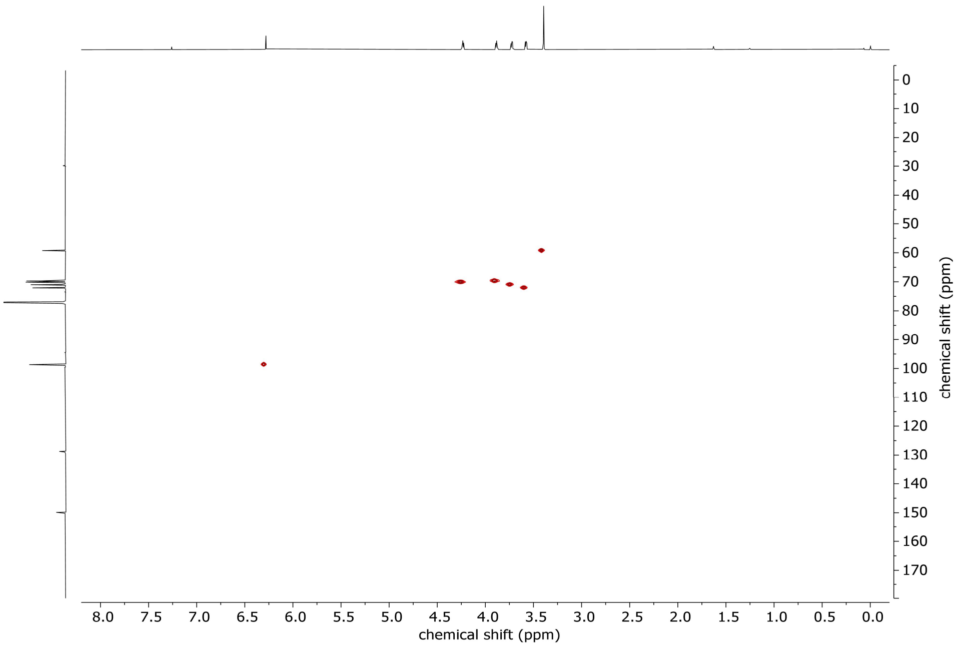

Figure S16. HSQC NMR spectrum ($CDCl_3$, 600/151 MHz, 298 K) of 3,6-bis(diethylene glycol monomethyl ether)thieno[3,2-*b*]thiophene (g_2TT).

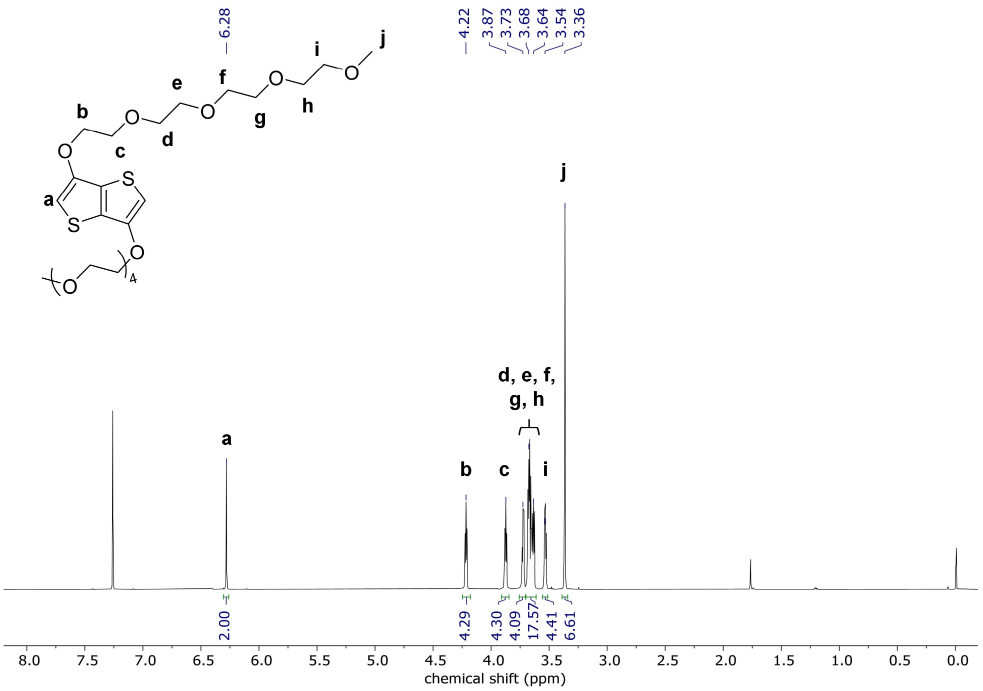

Figure S17. ^{1}H NMR spectrum ($CDCl_3$, 600 MHz, 298 K) of 3,6-bis(tetraethylene glycol monomethyl ether)thieno[3,2-*b*]thiophene (g_4TT).

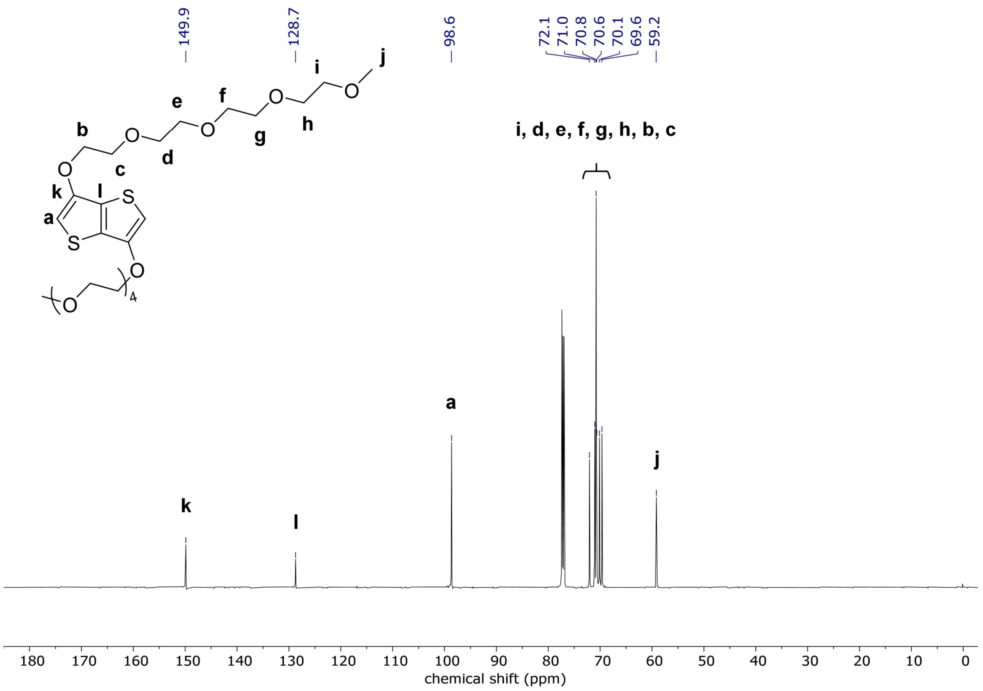

Figure S18. ^{13}C NMR spectrum ($CDCl_3$, 151 MHz, 298 K) of 3,6-bis(tetraethylene glycol monomethyl ether)thieno[3,2-*b*]thiophene (g_4TT).

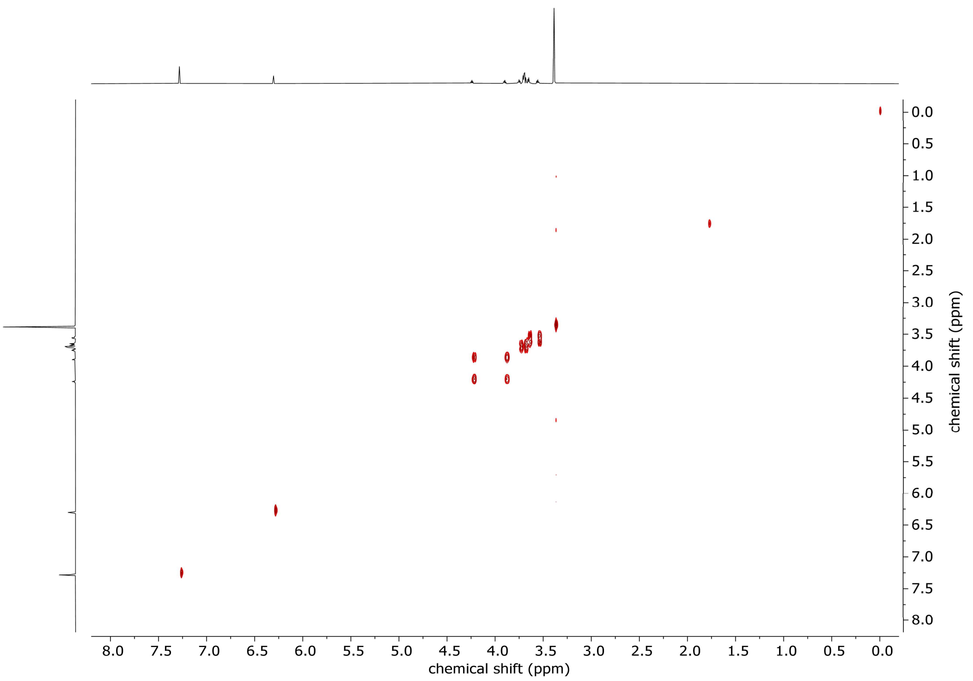

Figure S19. ^{1}H COSY NMR spectrum ($CDCl_3$, 600 MHz, 298 K) of 3,6-bis(tetraethylene glycol monomethyl ether)thieno[3,2-*b*]thiophene (g_4TT).

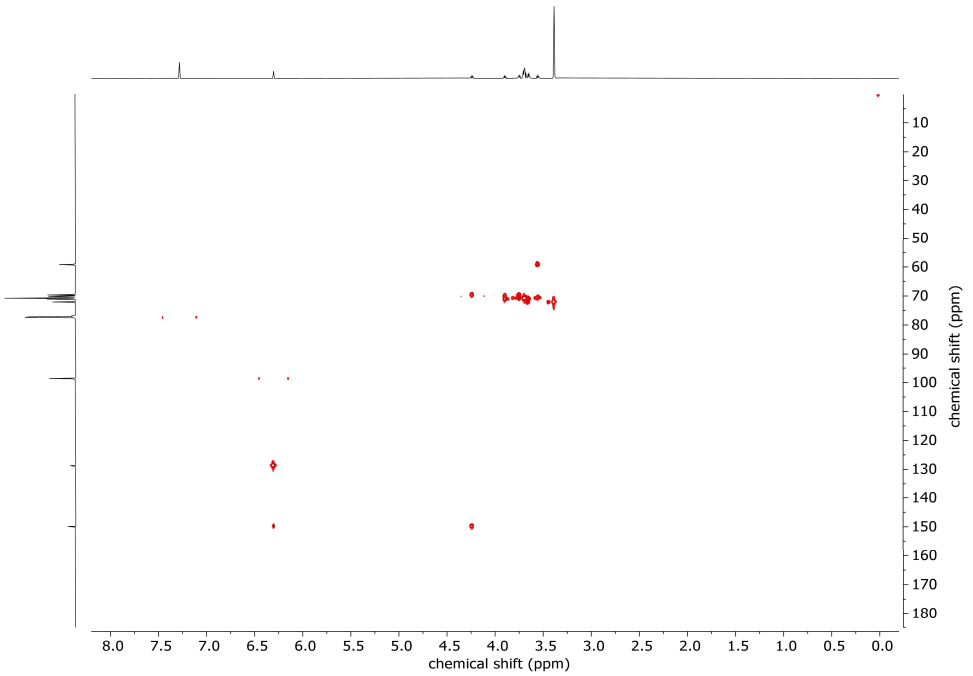

Figure S20. HMBC NMR spectrum ($CDCl_3$, 600/151 MHz, 298 K) of 3,6-bis(tetraethylene glycol monomethyl ether)thieno[3,2-*b*]thiophene (g_4TT).

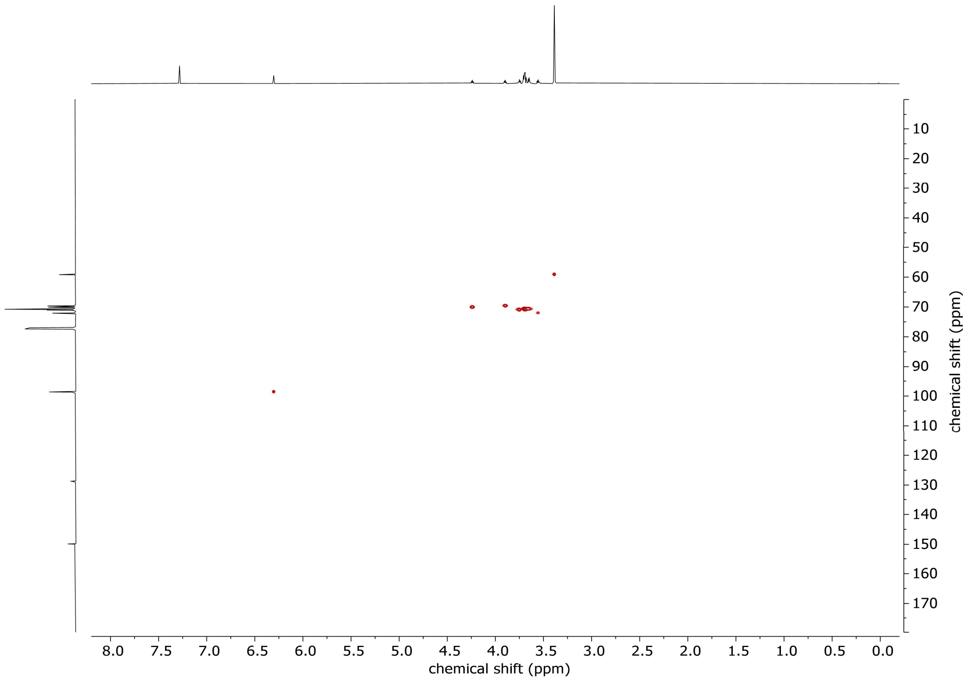

Figure S21. HSQC NMR spectrum ($CDCl_3$, 600/151 MHz, 298 K) of 3,6-bis(tetraethylene glycol monomethyl ether)thieno[3,2-*b*]thiophene (g_4TT).

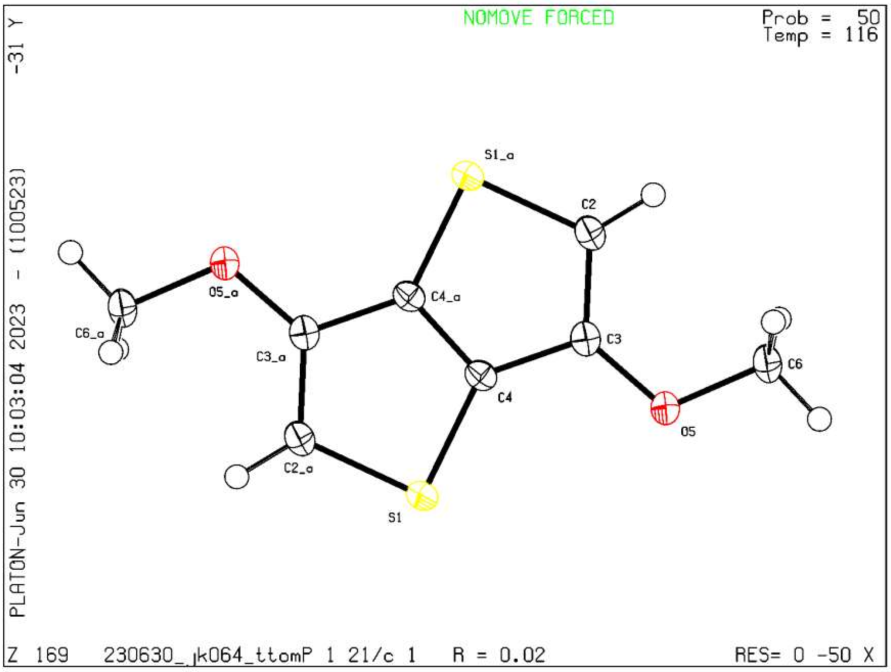

Figure S22. Single crystal X-ray ellipsoid plot (50 % probability) of 3,6-dimethoxythieno[3,2-*b*]thiophene (g_0TT). CCDC deposition number 2503697.

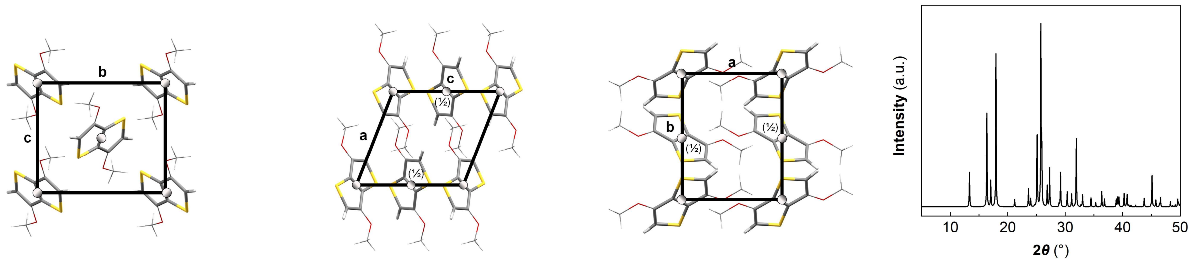

Figure S23. Unit cell of 3,6-dimethoxythieno[3,2-*b*]thiophene (g_0TT) crystals and associated powder X-ray diffraction pattern. CCDC deposition number 2503697.

Table S1. Crystal data and structure refinement for 3,6-dimethoxythieno[3,2-*b*]thiophene (g_0TT).

Identification code	230630_JK064_TTOMe2_auto
Empirical formula	C_4H_4OS
Formula weight	100.13
Temperature/K	116.05(11)
Crystal system	monoclinic
Space group	$P2_1/c$
a/Å	7.1506(3)
b/Å	8.3788(3)
c/Å	7.6478(3)
α/°	90
β/°	111.919(5)
γ/°	90
Volume/Å^3	425.08(3)
Z	4
ρ_{calc}g/cm^3	1.565
μ/mm^{-1}	5.304
F(000)	208
Radiation	Cu Kα (λ = 1.54184)
2Θ range for data collection/°	13.348 to 149.598
Index ranges	$-8 \leq h \leq 6$, $-10 \leq k \leq 10$, $-9 \leq l \leq 9$
Reflections collected	4098
Independent reflections	846 [R_{int} = 0.0325, R_{sigma} = 0.0227]
Data/restraints/parameters	846/0/57
Goodness-of-fit on F^2	1.091
Final R indexes [I>=2σ (I)]	R_1 = 0.0243, wR_2 = 0.0621
Final R indexes [all data]	R_1 = 0.0266, wR_2 = 0.0636
Largest diff. peak/hole / e Å^{-3}	0.33/-0.18

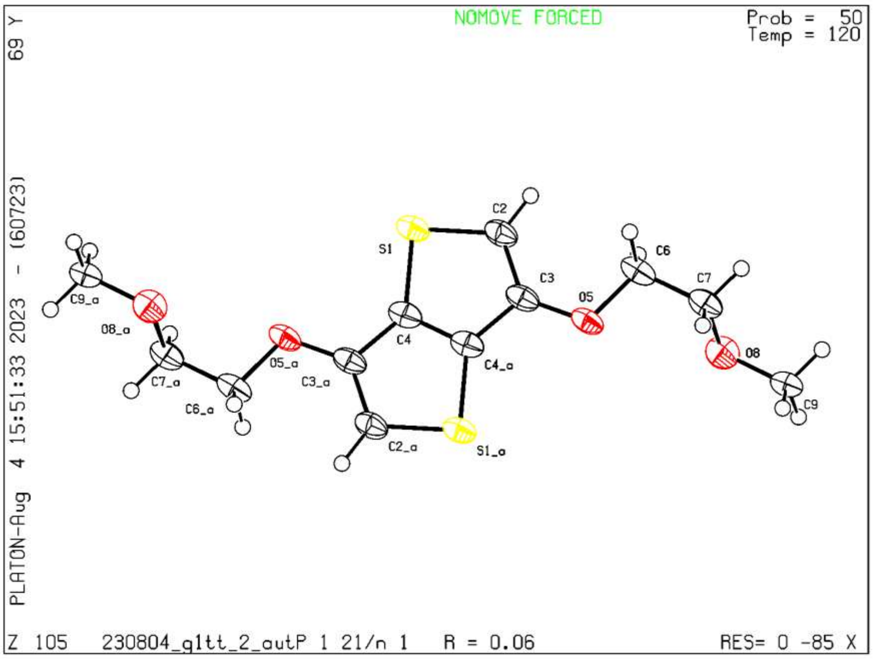

Figure S24. Single crystal X-ray ellipsoid plot (50 % probability) of 3,6-bis(monoethylene glycol monomethyl ether)thieno[3,2-*b*]thiophene (g_1TT). CCDC deposition number 2503699.

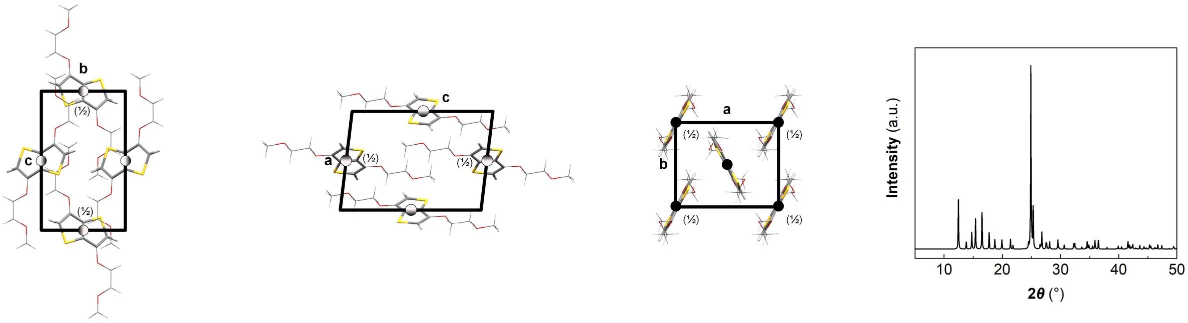

Figure S25. Unit cell of 3,6-bis(monoethylene glycol monomethyl ether)thieno[3,2-*b*]thiophene (g_1TT) crystals and associated powder X-ray diffraction pattern. CCDC deposition number 2503699.

Table S2. Crystal data and structure refinement for 3,6-bis(monoethylene glycol monomethyl ether)thieno[3,2-*b*]thiophene (g_1TT).

Identification code	230804_g1TT_2_auto
Empirical formula	$C_6H_8O_2S$
Formula weight	144.18
Temperature/K	120.02(13)
Crystal system	monoclinic
Space group	$P2_1/n$
a/Å	8.3423(5)
b/Å	7.0346(5)
c/Å	11.5647(8)
α/°	90
β/°	96.184(6)
γ/°	90
Volume/Å^3	674.73(8)
Z	4
ρ_{calc}g/cm^3	1.419
μ/mm^{-1}	3.63
F(000)	304
Radiation	Cu Kα (λ = 1.54184)
2Θ range for data collection/°	12.468 to 149.148
Index ranges	$-10 \leq h \leq 10$, $-5 \leq k \leq 8$, $-14 \leq l \leq 14$
Reflections collected	6371
Independent reflections	1339 [R_{int} = 0.0345, R_{sigma} = 0.0249]
Data/restraints/parameters	1339/0/83
Goodness-of-fit on F^2	1.087
Final R indexes [I>=2σ (I)]	R_1 = 0.0595, wR_2 = 0.1658
Final R indexes [all data]	R_1 = 0.0667, wR_2 = 0.1714
Largest diff. peak/hole / e Å^{-3}	0.85/-0.42

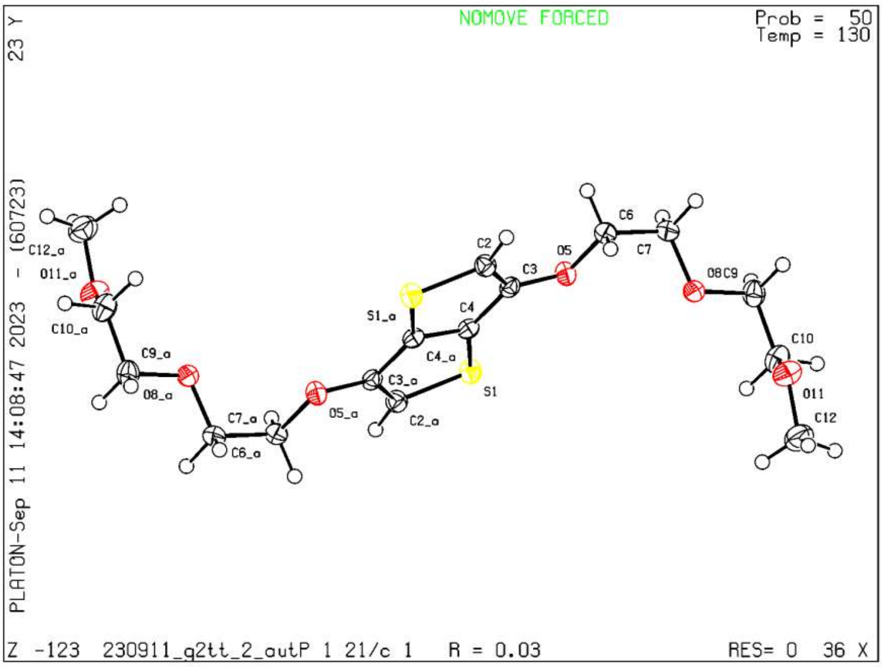

Figure S26. Single crystal X-ray ellipsoid plot (50 % probability) of 3,6-bis(diethylene glycol monomethyl ether)thieno[3,2-*b*]thiophene (g_2TT). CCDC deposition number 2503698.

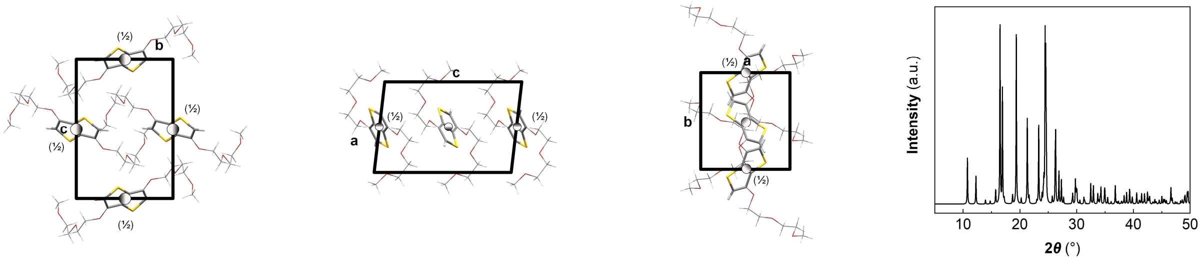

Figure S27. Unit cell of 3,6-bis(diethylene glycol monomethyl ether)thieno[3,2-*b*]thiophene (g_2TT) crystals and associated powder X-ray diffraction pattern. CCDC deposition number 2503698.

Table S3. Crystal data and structure refinement for 3,6-bis(diethylene glycol monomethyl ether)thieno[3,2-*b*]thiophene (g_2TT).

Identification code	230911_g2TT_2_auto
Empirical formula	$C_8H_{12}O_3S$
Formula weight	188.24
Temperature/K	130(1)
Crystal system	monoclinic
Space group	$P2_1/c$
a/Å	8.2828(2)
b/Å	8.7695(3)
c/Å	12.8067(4)
α/°	90
β/°	97.393(3)
γ/°	90
Volume/Å^3	922.49(5)
Z	4
ρ_{calc}g/cm^3	1.355
μ/mm^{-1}	2.866
F(000)	400
Radiation	Cu Kα (λ = 1.54184)
2Θ range for data collection/°	10.77 to 150.062
Index ranges	$-10 \leq h \leq 10$, $-10 \leq k \leq 10$, $-15 \leq l \leq 15$
Reflections collected	8859
Independent reflections	1841 [R_{int} = 0.0260, R_{sigma} = 0.0202]
Data/restraints/parameters	1841/0/110
Goodness-of-fit on F^2	1.044
Final R indexes [I>=2σ (I)]	R_1 = 0.0267, wR_2 = 0.0660
Final R indexes [all data]	R_1 = 0.0309, wR_2 = 0.0679
Largest diff. peak/hole / e Å^{-3}	0.26/-0.18

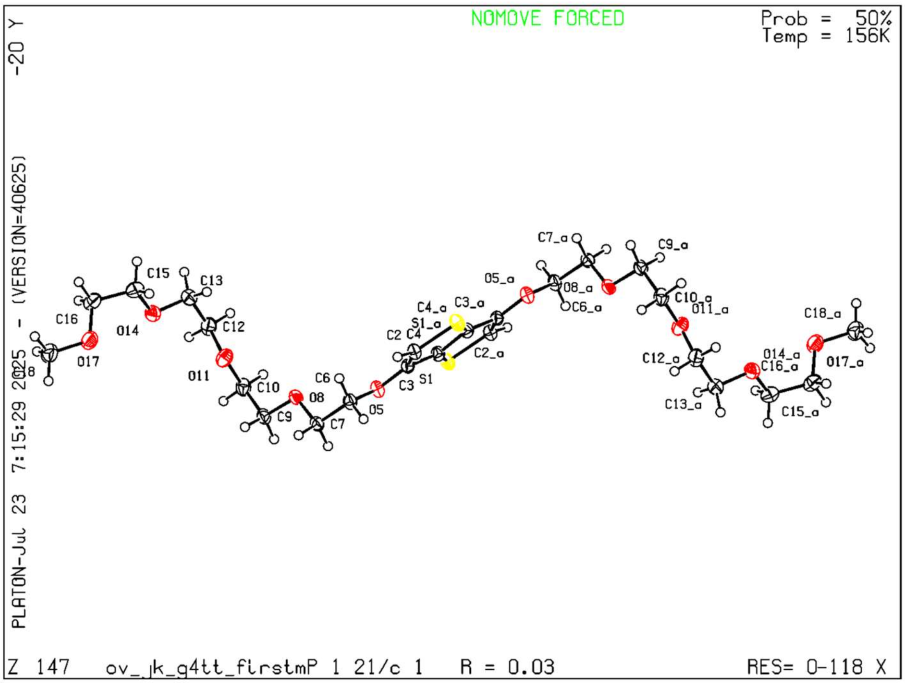

Figure S28. Single crystal X-ray ellipsoid plot (50 % probability) of Form I 3,6-bis(tetraethylene glycol monomethyl ether)thieno[3,2-*b*]thiophene (g_4TT). CCDC deposition number 2504797.

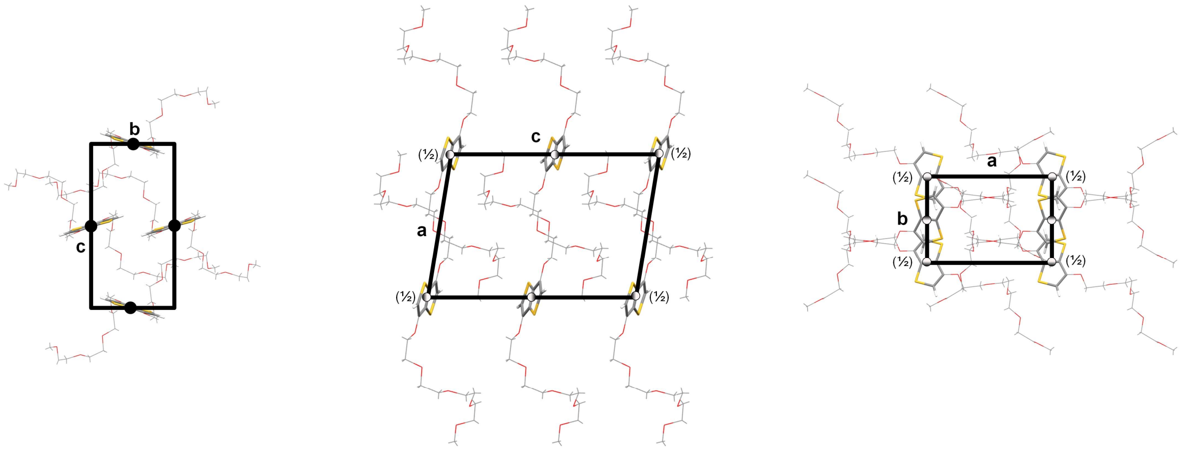

Figure S29. Unit cell of Form I 3,6-bis(tetraethylene glycol monomethyl ether)thieno[3,2-*b*]thiophene (g_4TT) and associated powder X-ray diffraction pattern. CCDC deposition number 2504797.

Table S4. Crystal data and structure refinement for Form I 3,6-bis(tetraethylene glycol monomethyl ether)thieno[3,2-*b*]thiophene (g_4TT).

Identification code	JK_g4TT_firstmelt2_auto
Empirical formula	$C_{12}H_{19}O_5S$
Formula weight	275.33
Temperature/K	155.7(2)
Crystal system	monoclinic
Space group	$P2_1/c$
a/Å	11.22133(18)
b/Å	7.82649(14)
c/Å	15.8821(3)
α/°	90
β/°	99.1774(15)
γ/°	90
Volume/Å^3	1376.97(4)
Z	4
ρ_{calc}g/cm^3	1.328
μ/mm^{-1}	2.202
F(000)	588
Radiation	Cu Kα (λ = 1.54184)
2Θ range for data collection/°	7.982 to 150.138
Index ranges	-9 ≤ h ≤ 13, -9 ≤ k ≤ 9, -19 ≤ l ≤ 19
Reflections collected	13195
Independent reflections	2748 [R_{int} = 0.0328, R_{sigma} = 0.0254]
Data/restraints/parameters	2748/0/172
Goodness-of-fit on F^2	1.051
Final R indexes [I>=2σ (I)]	R_1 = 0.0306, wR_2 = 0.0779
Final R indexes [all data]	R_1 = 0.0373, wR_2 = 0.0815
Largest diff. peak/hole / e Å^{-3}	0.36/-0.20

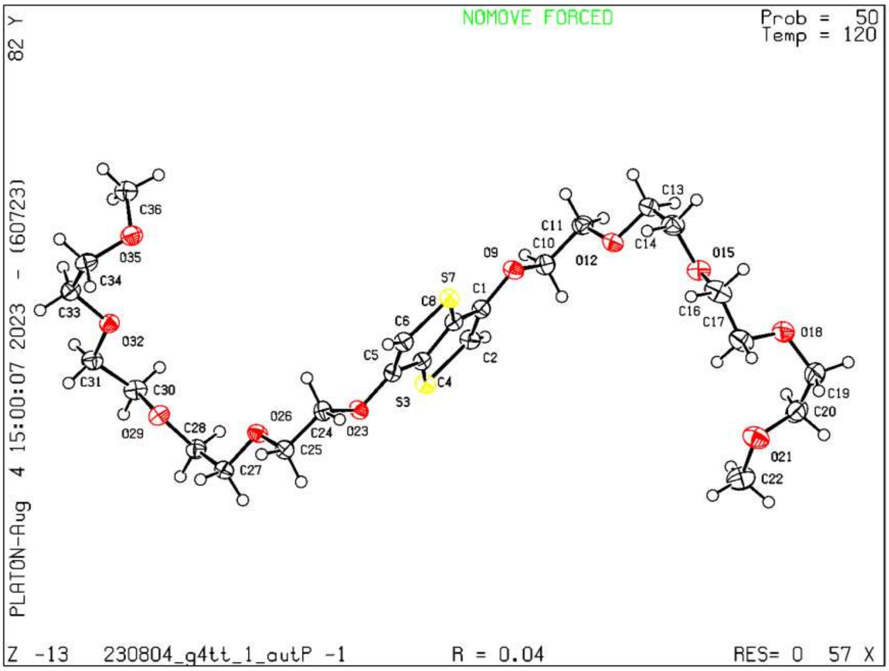

Figure S30. Single crystal X-ray ellipsoid plot (50 % probability) of Form II 3,6-bis(tetraethylene glycol monomethyl ether)thieno[3,2-*b*]thiophene (g₄TT). CCDC deposition number 2503700.

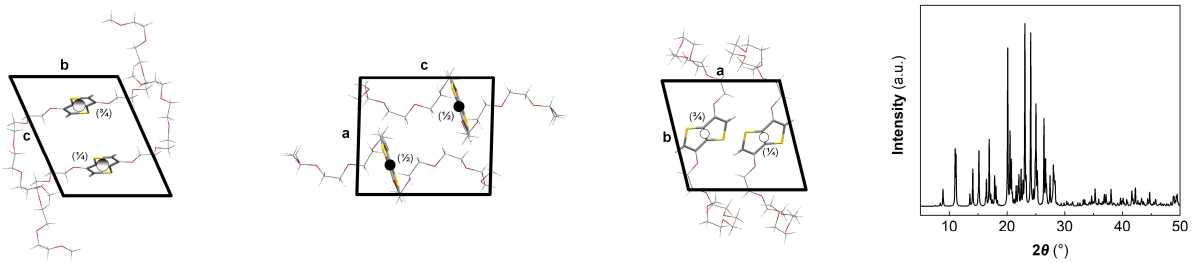

Figure S31. Unit cell of Form II 3,6-bis(tetraethylene glycol monomethyl ether)thieno[3,2-*b*]thiophene (g₄TT) crystals. CCDC deposition number 2503700.

Table S5. Crystal data and structure refinement for Form II 3,6-bis(tetraethylene glycol monomethyl ether)thieno[3,2-*b*]thiophene (g_4TT).

Identification code	230804_g4TT_1_auto
Empirical formula	$C_{24}H_{40}O_{10}S_2$
Formula weight	552.68
Temperature/K	119.98(10)
Crystal system	triclinic
Space group	P-1
a/Å	10.8044(3)
b/Å	11.2170(3)
c/Å	12.8614(5)
α/°	65.538(3)
β/°	85.706(3)
γ/°	75.968(3)
Volume/Å^3	1375.88(8)
Z	2
ρ_{calc}g/cm^3	1.334
μ/mm^{-1}	2.204
F(000)	592
Radiation	Cu Kα (λ = 1.54184)
2Θ range for data collection/°	7.554 to 150.73
Index ranges	$-13 \le h \le 12$, $-14 \le k \le 13$, $-16 \le l \le 15$
Reflections collected	25084
Independent reflections	5470 [R_{int} = 0.0474, R_{sigma} = 0.0322]
Data/restraints/parameters	5470/0/327
Goodness-of-fit on F^2	1.043
Final R indexes [I>=2σ (I)]	R_1 = 0.0399, wR_2 = 0.1070
Final R indexes [all data]	R_1 = 0.0472, wR_2 = 0.1130
Largest diff. peak/hole / e Å^{-3}	0.41/-0.44

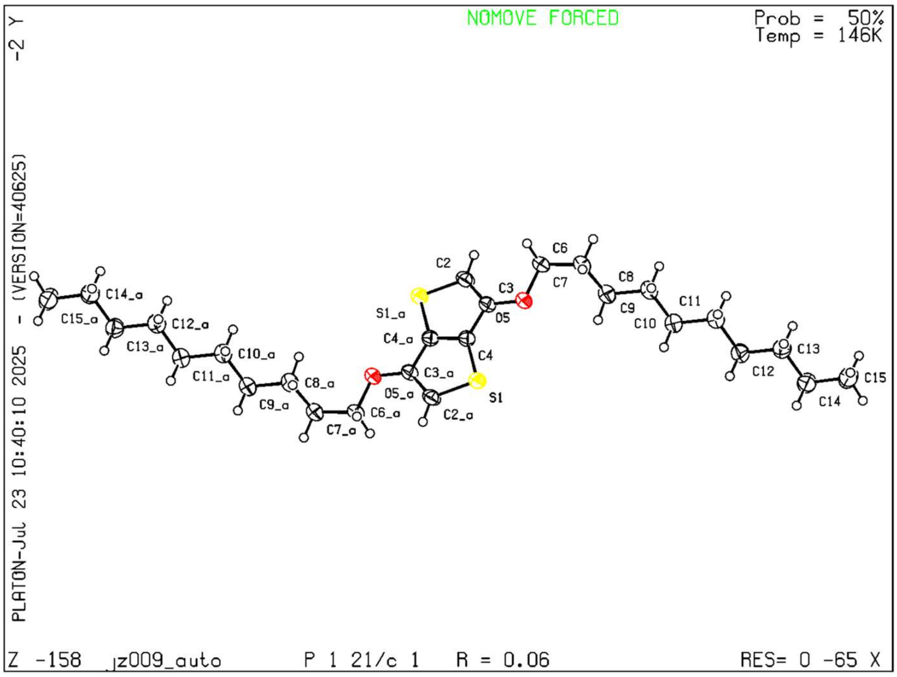

Figure S32. Single crystal X-ray ellipsoid plot (50 % probability) of 3,6-bis(decyloxy)thieno[3,2-*b*]thiophene (aTT). CCDC deposition number 2503701.

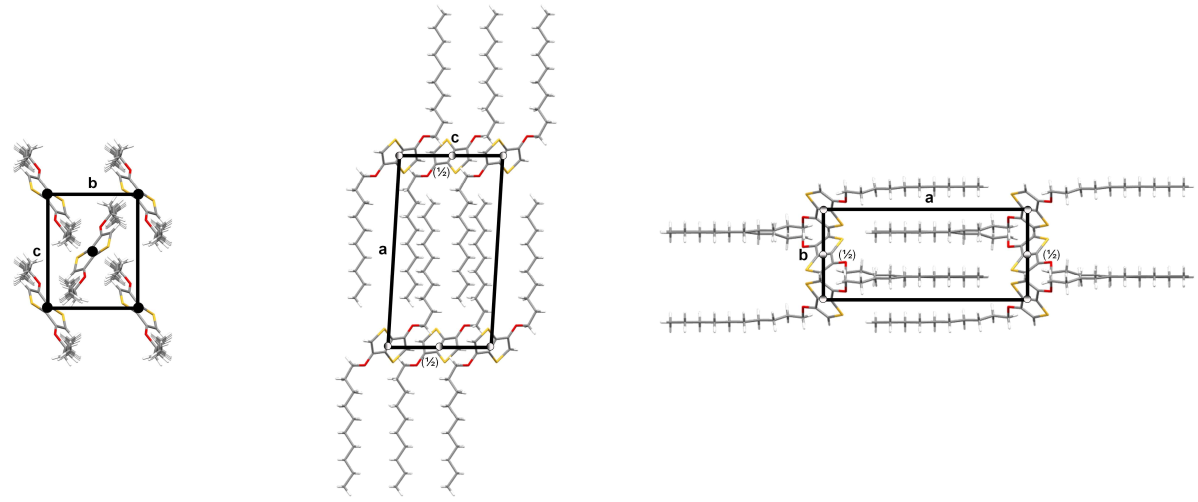

Figure S33. Unit cell of 3,6-bis(decyloxy)thieno[3,2-*b*]thiophene (aTT) crystals. CCDC deposition number 2503701.

Table S6. Crystal data and structure refinement for 3,6-bis(decyloxy)thieno[3,2-*b*]thiophene (aTT).

Identification code	JZ009_auto
Empirical formula	$C_{13}H_{22}OS$
Formula weight	226.36
Temperature/K	146.37(18)
Crystal system	monoclinic
Space group	$P2_1/c$
a/Å	17.7643(9)
b/Å	7.7416(5)
c/Å	9.7298(5)
α/°	90
β/°	93.281(5)
γ/°	90
Volume/Å^3	1335.89(13)
Z	4
ρ_{calc}g/cm^3	1.126
μ/mm^{-1}	1.933
F(000)	496
Radiation	Cu Kα (λ = 1.54184)
2Θ range for data collection/°	4.982 to 150.43
Index ranges	$-22 \leq h \leq 22$, $-9 \leq k \leq 9$, $-8 \leq l \leq 11$
Reflections collected	12253
Independent reflections	2657 [R_{int} = 0.1074, R_{sigma} = 0.0777]
Data/restraints/parameters	2657/0/137
Goodness-of-fit on F^2	1.011
Final R indexes [I>=2σ (I)]	R_1 = 0.0592, wR_2 = 0.1522
Final R indexes [all data]	R_1 = 0.0959, wR_2 = 0.1781
Largest diff. peak/hole / e Å^{-3}	0.50/-0.51

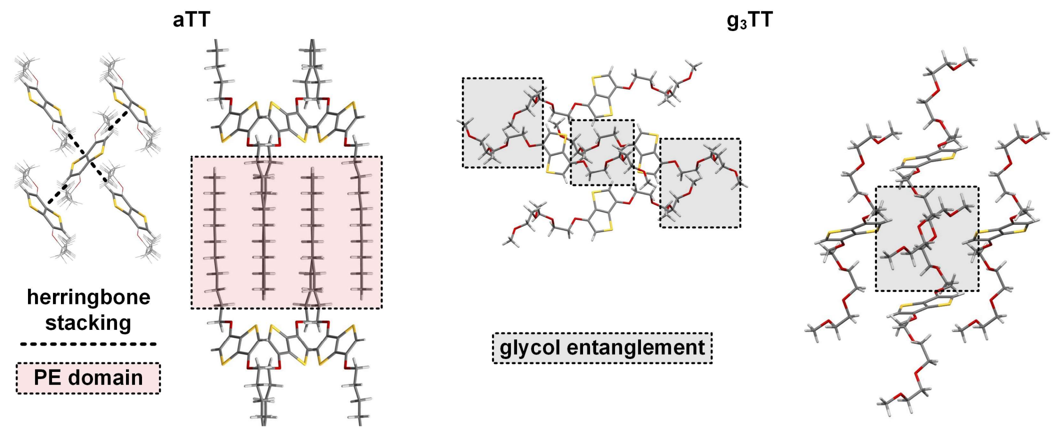

Figure S34. Structure comparison between alkoxy chain and ethylene glycol chain with the same atom number in thieno[3,2-*b*]thiophene crystals.

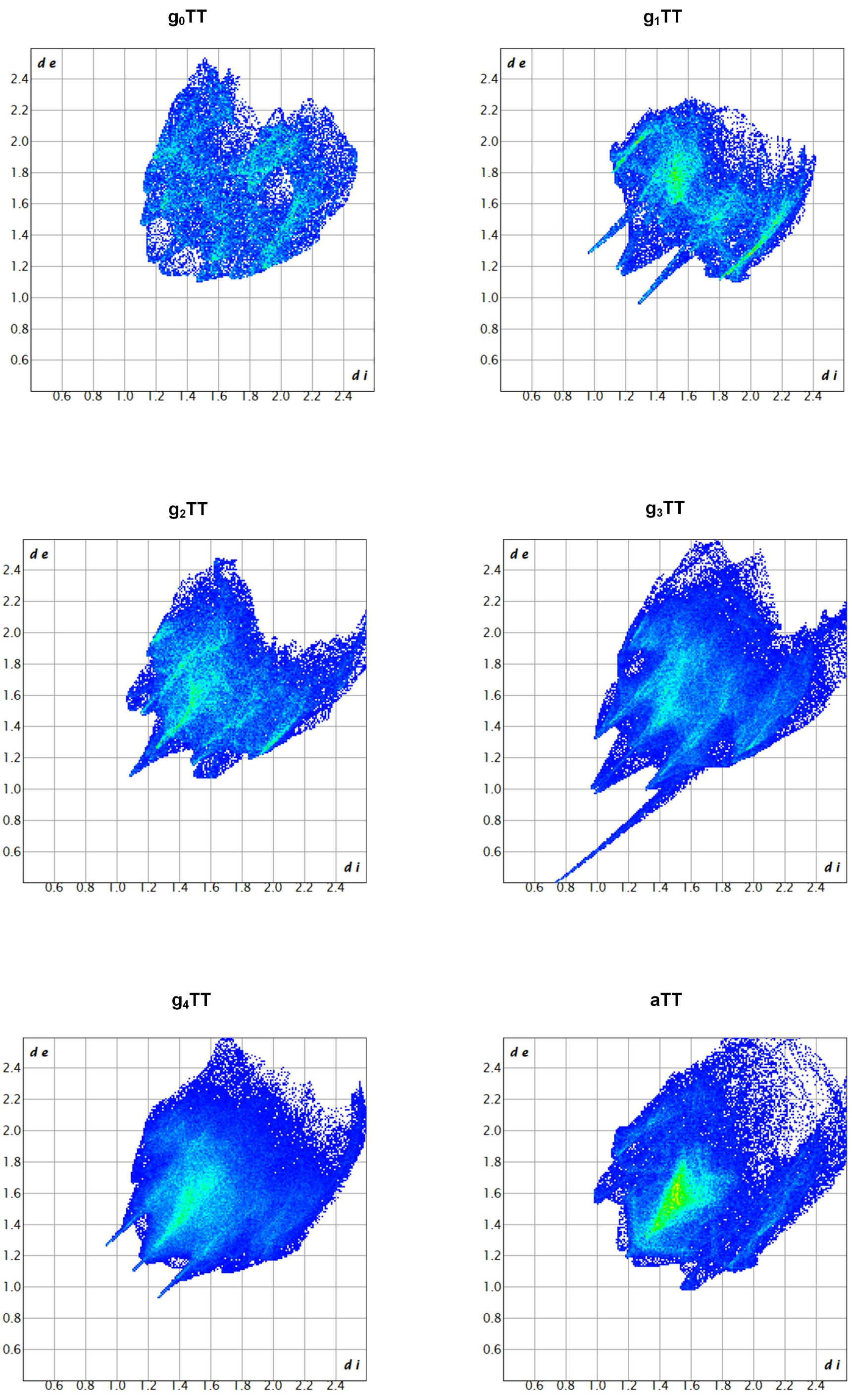

Figure S35. Hirshfeld surface fingerprints for g_0TT to g_4TT and aTT, plotted as d_i (distance from the surface to the nearest interior atom) versus d_e (distance to the nearest exterior atom). The color scale highlights the density of surface contacts, with greener regions indicating more frequent interactions. Broad, diffuse wings correspond to a combination of C–H···π and van der Waals interactions. Spikes at low d_i/d_e values reflect short H···H contacts.

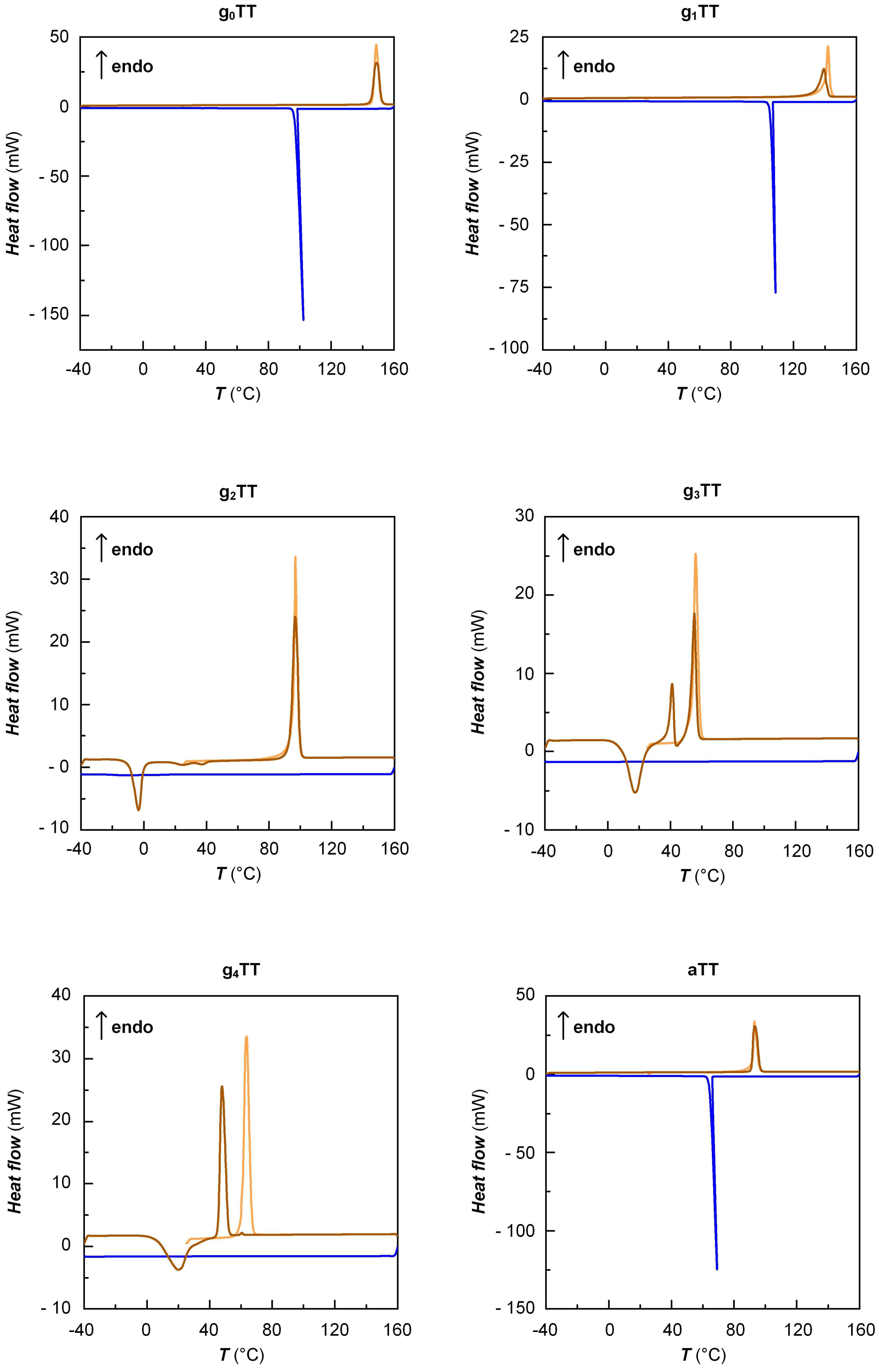

Figure S36. First and second differential scanning calorimetry heating thermograms given in pale orange and brown (-40 °C to 160 °C under nitrogen atmosphere using a heating rate of 10 °C min^{-1}) and first differential scanning calorimetry cooling thermograms given in blue (160 °C to -40 °C under nitrogen atmosphere using a cooling rate of 10 °C min^{-1}).

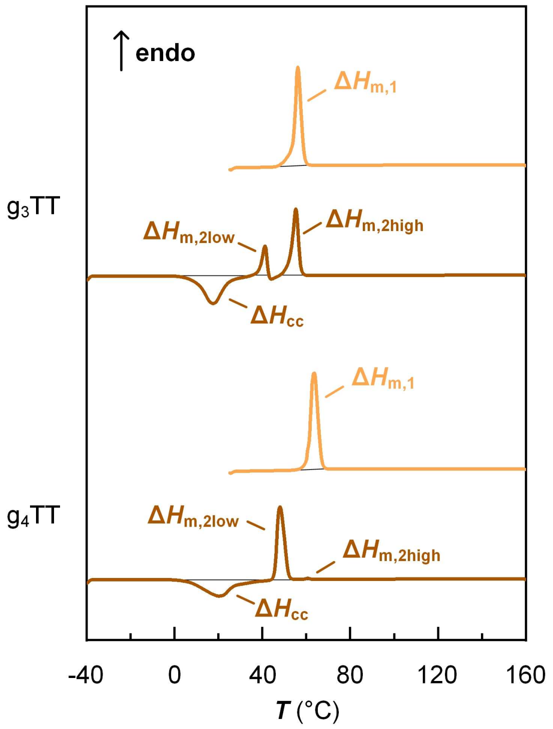

Figure S37. First and second differential scanning calorimetry heating thermograms (25 °C to 160 °C and -40 °C to 160 °C under nitrogen atmosphere using a heating rate of 10 °C min^{-1}) of g_3TT (top) and g_4TT (bottom) with associated enthalpy changes determined by peak integration. First heating in pale orange; second heating in brown.

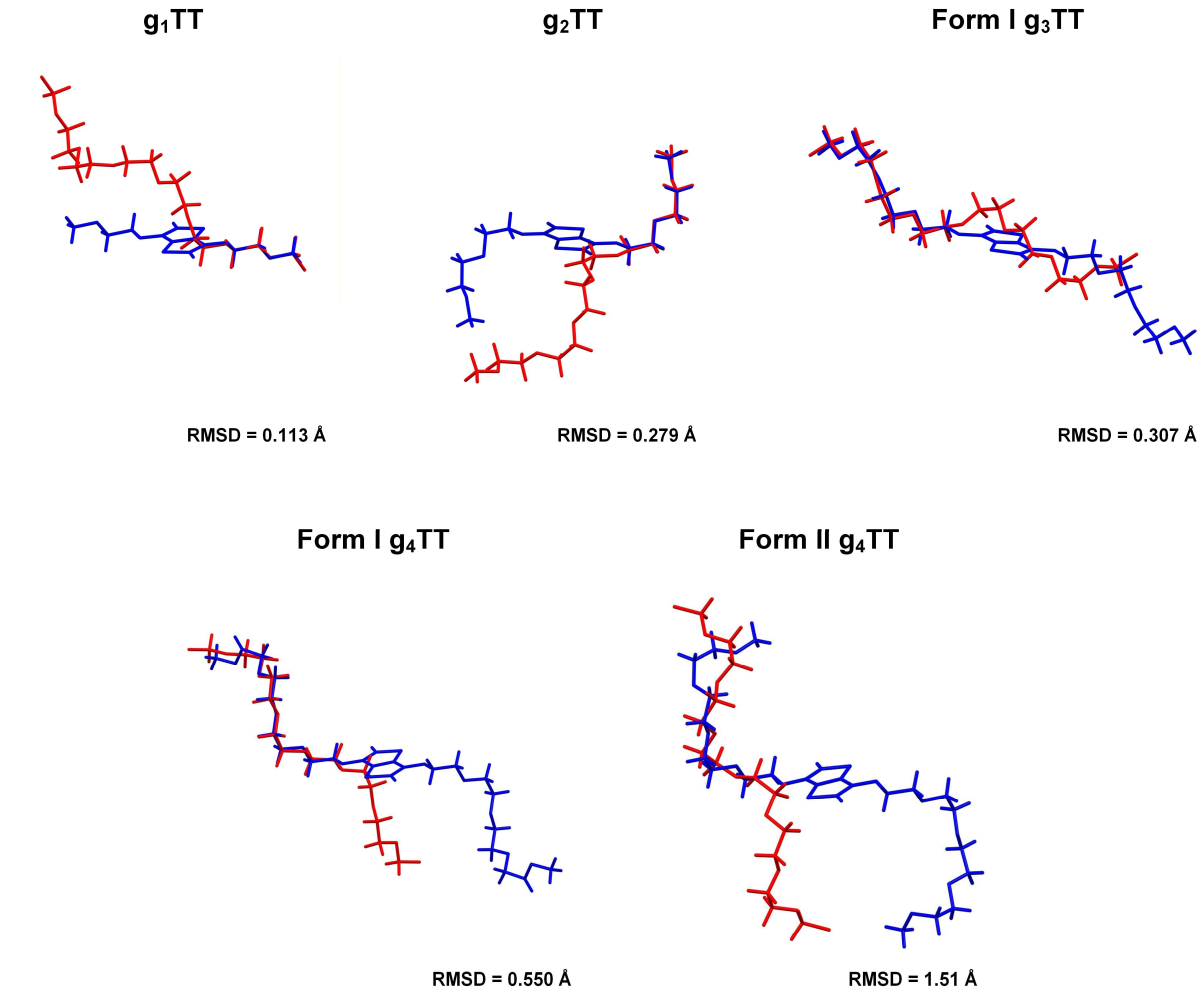

Figure S38. Conformation of oligoethylene glycol pendent groups in thieno[3,2-*b*]thiophene crystals. Root-mean-square deviation (RMSD) is defined as the square root of the mean squared error between atoms of oligoethylene glycol pendent groups and atoms of polyethylene oxide chain with the conformation in a polyethylene oxide crystal with a 7_2 helical conformation ($\sqrt{\sum_{i=1}^{n}(TT_i - PEO_i)^2}$, where i represents atoms).

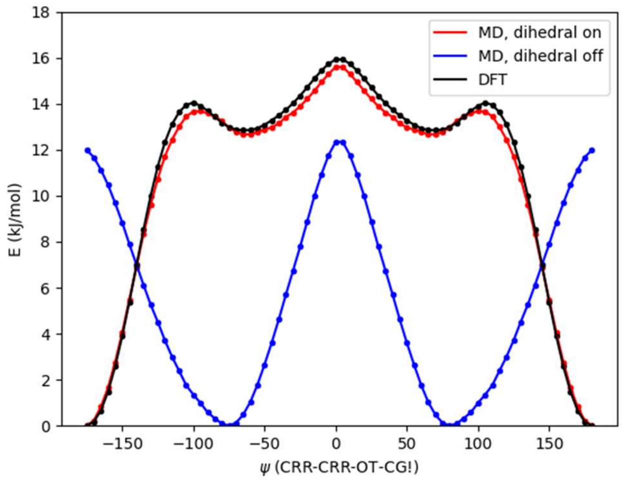

Figure S39. Torsional scan for the rotation of the methyl unit around the oxygen bonded to the thienothiophene unit. The blue line represents the 'background potential' where the parameters for the dihedral being parametrized are turned off. The black line is the potential energy surface derived from DFT, and the red line is the reparametrized potential calculated through MD.

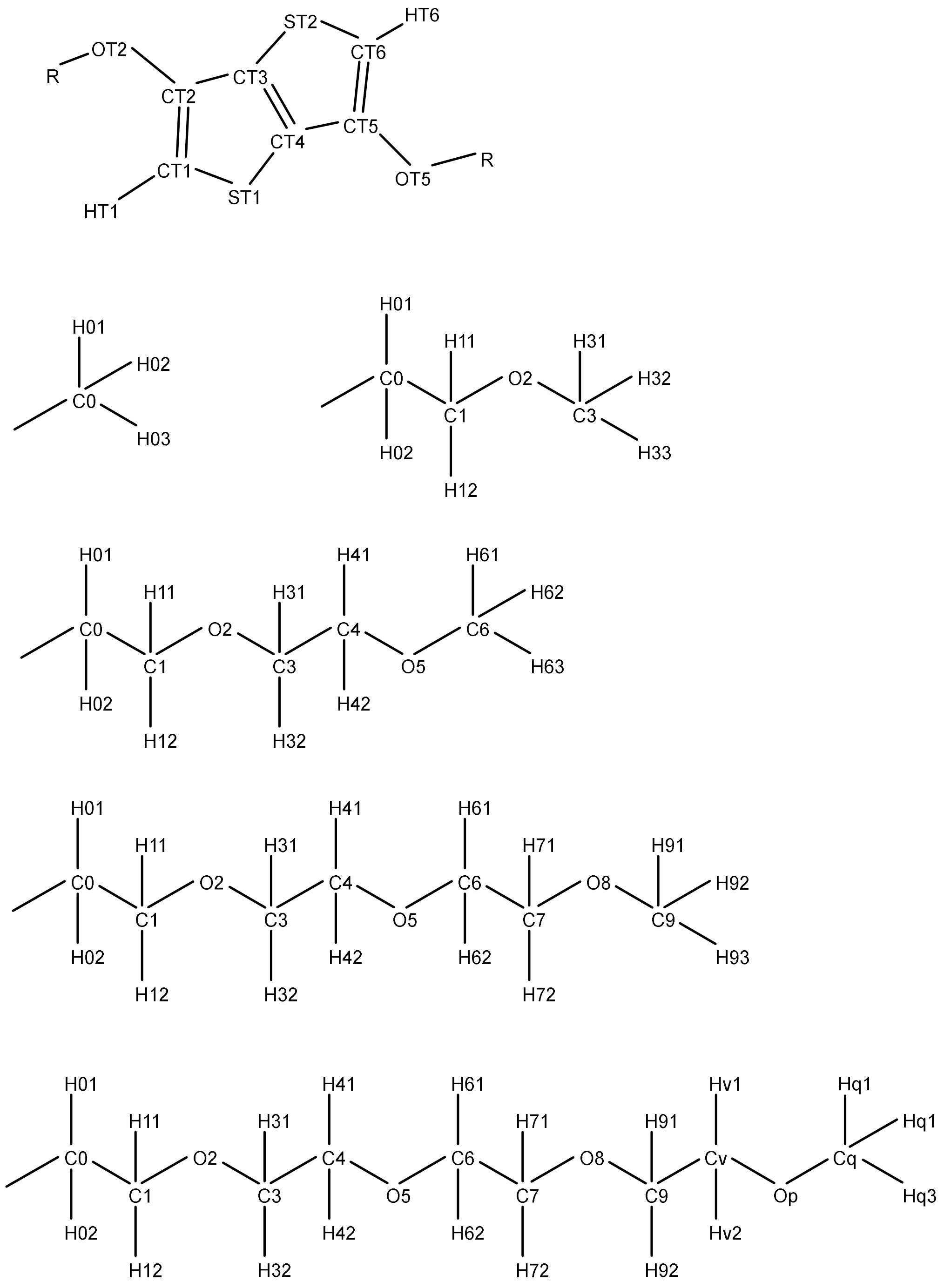

Figure S40. Atom names for studied structures relevant for supplementary tables.

Table S7. Partial charges for the thieno[3,2-*b*]thiophene core.

**Thieno[3,2-*b*]thiophene core**		
**Atom Name**	**Atom type**	**Partial Charge**
CT1	CRR	-0.379
CT2	CRR	0.413
CT3	CRR	-0.028
CT4	CRR	-0.028
CT5	CRR	0.413
CT6	CRR	-0.379
ST1	SRR	0.017
ST2	SRR	0.017
HT1	HRR	0.272
HT6	HRR	0.272
POT2	OT	-0.295
OT5	OT	-0.295

Table S8. Partial charges for the g_0 chain.

**g_0**		
**Atom Name**	**Atom type**	**Partial Charge**
C0	CG!	-0.249
H01	HG	0.083
H02	HG	0.083
H03	HG	0.083
Caa	CG!	-0.249
Ha1	HG	0.083
Ha2	HG	0.083
Ha3	HG	0.083
C0	CG!	-0.249
H01	HG	0.083
H02	HG	0.083
H03	HG	0.083

Table S9. Partial charges for the g_1 chain.

	g_1	
**Atom Name**	**Atom type**	**Partial Charge**
C0	CG!	-0.264
H01	HG	0.067
H02	HG	0.067
C1	CG	0.219
H11	HG	0.068
H12	HG	0.068
O2	OG	-0.412
C3	CG	-0.014
H31	HG	0.067
H32	HG	0.067
H33	HG	0.067
Caa	CG!	-0.264
Ha1	HG	0.067
Ha2	HG	0.067
Cb	CG	0.219
Hb1	HG	0.068
Hb2	HG	0.068
Oc	OG	-0.412
Cd	CG	-0.014
Hd1	HG	0.067
Hd2	HG	0.067
Hd3	HG	0.067

Table S10. Partial charges for the g_2 chain.

	g_2	
**Atom Name**	**Atom type**	**Partial Charge**
C0	CG!	-0.242
H01	HG	0.089
H02	HG	0.089
C1	CG	0.012
H11	HG	0.089
H12	HG	0.089
O2	OG	-0.384
C3	CG	0.004
H31	HG	0.089

H32	HG	0.089
C4	CG	0.007
H41	HG	0.089
H42	HG	0.089
O5	OG	-0.384
C6	CG	0.007
H61	HG	0.089
H62	HG	0.089
H63	HG	0.089
Caa	CG!	-0.242
Ha1	HG	0.089
Ha2	HG	0.089
Cb	CG	0.012
Hb1	HG	0.089
Hb2	HG	0.089
Oc	OG	-0.384
Cd	CG	0.004
Hd1	HG	0.089
Hd2	HG	0.089
Ce	CG	0.007
He1	HG	0.089
He2	HG	0.089
Of	OG	-0.384
Cg	CG	0.007
Hg1	HG	0.089
Hg2	HG	0.089
Hg3	HG	0.089

Table S11. Partial charges for the g_3 chain.

g_3		
**Atom Name**	**Atom type**	**Partial Charge**
C0	CG!	0.051
H01	HG	0.014
H02	HG	0.014
C1	CG	0.448
H11	HG	-0.048
H12	HG	-0.048
O2	OG	-0.539
C3	CG	0.209
H31	HG	-0.001
H32	HG	-0.001

C4	CG	0.218
H41	HG	-0.002
H42	HG	-0.002
O5	OG	-0.437
C6	CG	0.153
H61	HG	0.012
H62	HG	0.012
C7	CG	0.231
H71	HG	0.001
H72	HG	0.001
O8	OG	-0.484
C9	CG	0.231
H91	HG	-0.011
H92	HG	-0.011
H93	HG	-0.011
Caa	CG!	0.051
Ha1	HG	0.014
Ha2	HG	0.014
Cb	CG	0.448
Hb1	HG	-0.048
Hb2	HG	-0.048
Oc	OG	-0.539
Cd	CG	0.209
Hd1	HG	-0.001
Hd2	HG	-0.001
Ce	CG	0.218
He1	HG	-0.002
He2	HG	-0.002
Of	OG	-0.437
Cg	CG	0.153
Hg1	HG	0.012
Hg2	HG	0.012
Ch	CG	0.231
Hh1	HG	0.001
Hh2	HG	0.001
Oi	OG	-0.484
Cj	CG	0.231
Hj1	HG	-0.011
Hj2	HG	-0.011
Hj3	HG	-0.011

Table S12. Partial charges for the g_4 chain.

	g_4	
**Atom Name**	**Atom type**	**Partial Charge**
C0	CG!	-0.24
H01	HG	0.09
H02	HG	0.09
C1	CG	0.016
H11	HG	0.09
H12	HG	0.09
O2	OG	-0.389
C3	CG	0.009
H31	HG	0.09
H32	HG	0.09
C4	CG	0.012
H41	HG	0.09
H42	HG	0.09
O5	OG	-0.389
C6	CG	0.012
H61	HG	0.09
H62	HG	0.09
C7	CG	0.006
H71	HG	0.09
H72	HG	0.09
O8	OG	-0.389
C9	CG	0.012
H91	HG	0.09
H92	HG	0.09
Cv	CG	0.006
Hv1	HG	0.09
Hv2	HG	0.09
Op	OG	-0.389
Cq	CG	0.012
Hq1	HG	0.09
Hq2	HG	0.09
Hq3	HG	0.09
Caa	CG!	-0.24
Ha1	HG	0.09
Ha2	HG	0.09
Cb	CG	0.016
Hb1	HG	0.09
Hb2	HG	0.09
Oc	OG	-0.389
Cd	CG	0.009
Hd1	HG	0.09

Hd2	HG	0.09
Ce	CG	0.012
He1	HG	0.09
He2	HG	0.09
Of	OG	-0.389
Cg	CG	0.012
Hg1	HG	0.09
Hg2	HG	0.09
Ch	CG	0.006
Hh1	HG	0.09
Hh2	HG	0.09
Oi	OG	-0.389
Cj	CG	0.012
Hj1	HG	0.09
Hj2	HG	0.09
Ck	CG	0.006
Hk1	HG	0.09
Hk2	HG	0.09
Ol	OG	-0.389
Cm	CG	0.012
Hm1	HG	0.09
Hm2	HG	0.09
Hm3	HG	0.09

Table S13. Ryckaert-Bellemans dihedral parameters.

Dihedral				C_0	C_1	C_2	C_3	C_4	C_5
CRR	CRR	OT	CG!	5.1629	-9.5281	-17.9603	1.1334	1.5667	0.8426
CRR	OT	CG!	CG	1.715	2.845	1.046	-5.607	0	0
CG	CG	OG	CG	1.715	2.845	1.046	-5.607	0	0
CG!	CG	OG	CG	1.715	2.845	1.046	-5.607	0	0
HG	CG	OG	CG	1.59	4.77	0	-6.36	0	0
OG	CG	CG	OG	-1.151	1.151	0	0	0	0

Table S14. Harmonic bond potential parameters, taken from OPLS.[3]

Bond		b_0	k_b	OPLS Equivalent	
CRR	CRR	0.14	392459.2	CA	CA
SRR	CRR	0.176	209200	S	CA
CRR	HRR	0.108	307105.6	CA	HA
CG!	CG	0.1529	224262.4	CT	CT
CG!	HG	0.109	284512	CT	HC
CG	CG	0.1529	224262.4	CT	CT
CG	HG	0.109	284512	CT	HC
CG	OG	0.141	267776	CT	OS

Table S15. Harmonic bond potential parameters.[7] Parameter in bold parameterized in this work.

Angle			θ_0	k
OT	CG!	CG	109.5	418.4
CG	CG	OG	109.5	418.4
CG	OG	CG	109.5	502.08
CG	CG	HG	110.7	313.8
OG	CG	HG	109.5	292.88
HG	CG	HG	107.8	276.144
HG	CG!	HG	107.8	276.144
HG	CG!	CG	110.7	313.8
CG!	CG	HG	110.7	313.8
CG!	CG	OG	109.5	418.4
CRR	CRR	CRR	112.5	502.08
CRR	SRR	CRR	91	502.08
HRR	CRR	CRR	124	292.88
SRR	CRR	CE	119	502.08
CRR	CRR	SRR	112	502.08
CRR	SRR	CRR	91	502.08
SRR	CRR	HRR	124	292.88
**CRR**	**CRR**	**OT**	**119**	**850**

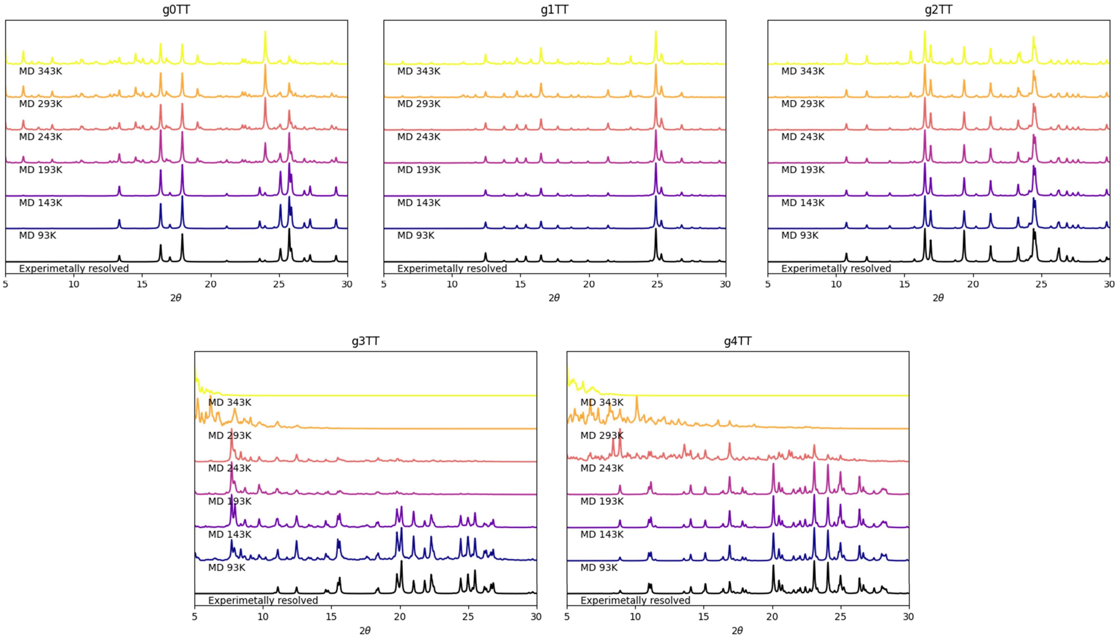

Figure S41. Force field validity test.

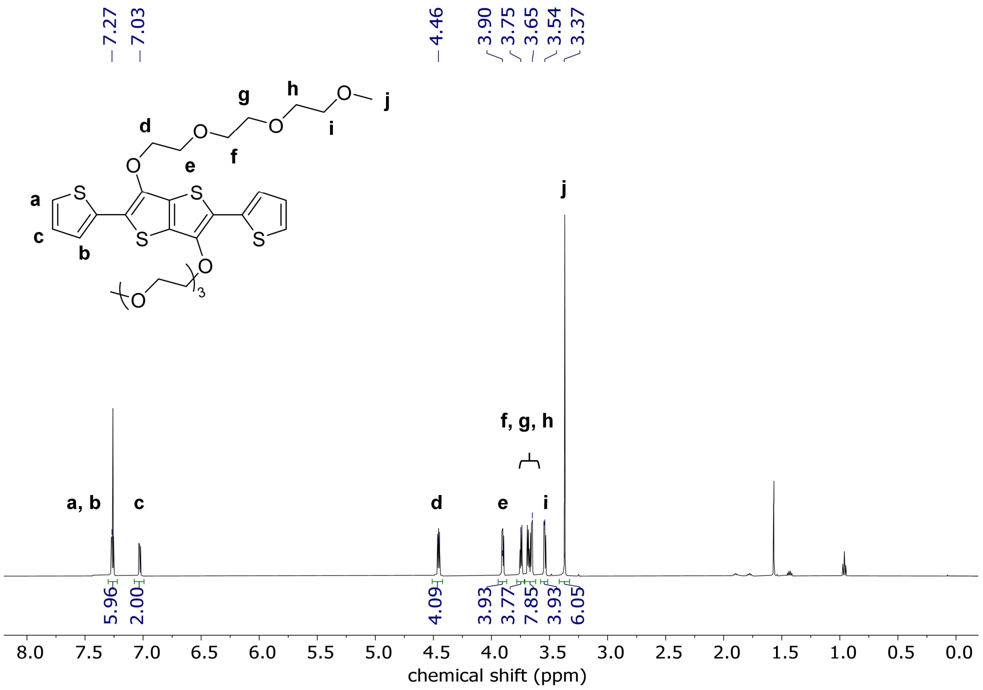

Figure S42. ^{1}H NMR spectrum ($CDCl_3$, 600 MHz, 298 K) of 3,6-bis(triethylene glycol monomethyl ether)-2,5-di(thiophen-2-yl)thieno[3,2-*b*]thiophene (T-g_3TT-T).

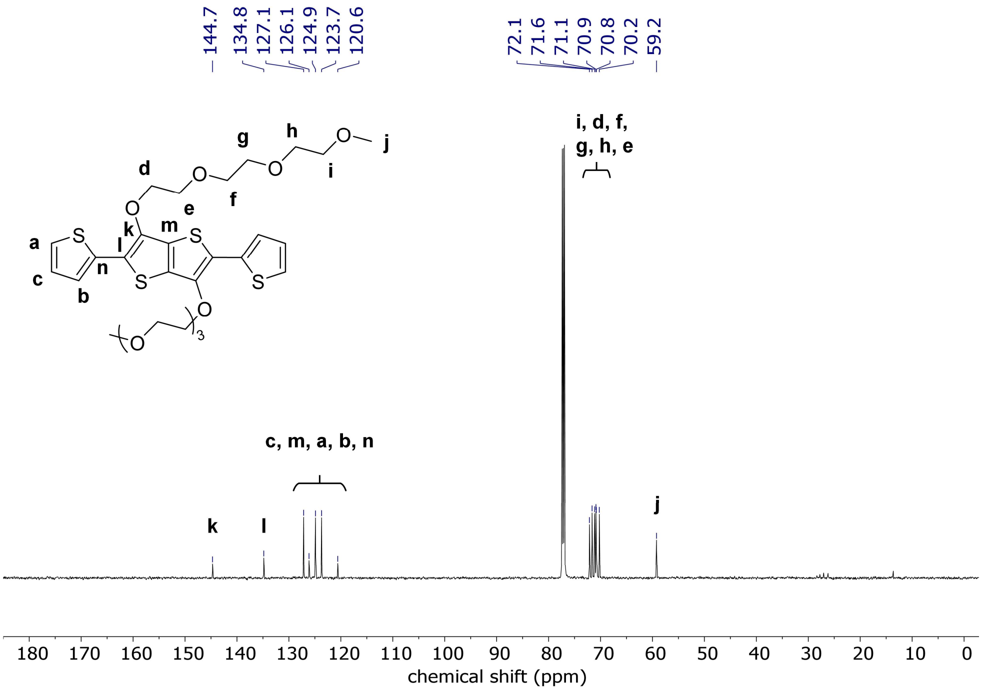

Figure S43. ^{13}C NMR spectrum ($CDCl_3$, 151 MHz, 298 K) of 3,6-bis(triethylene glycol monomethyl ether)-2,5-di(thiophen-2-yl)thieno[3,2-*b*]thiophene (T-g_3TT-T).

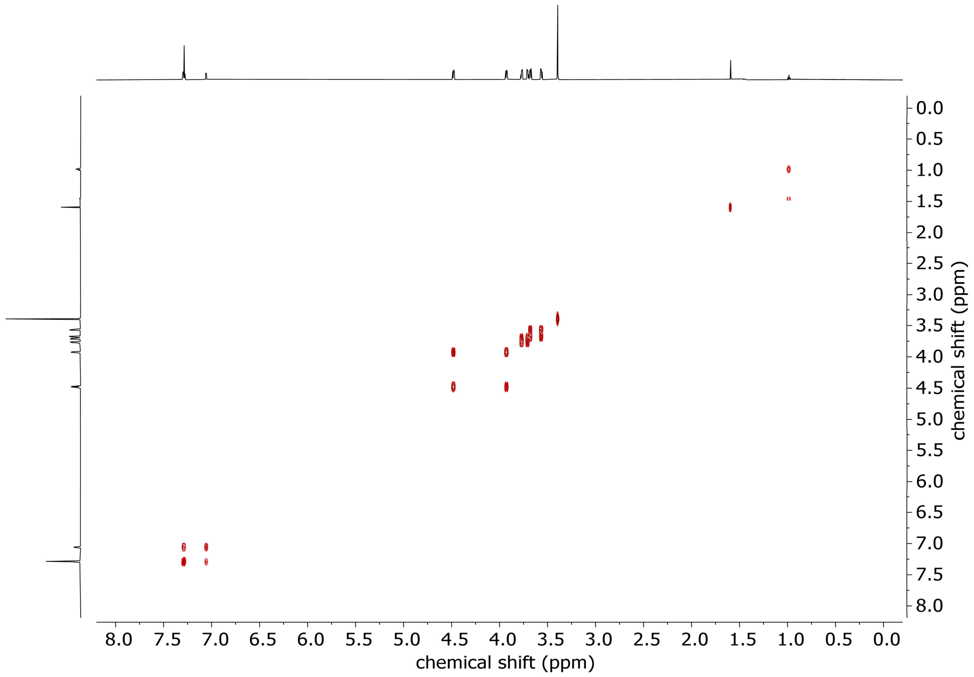

Figure S44. ^{1}H COSY NMR spectrum ($CDCl_3$, 600 MHz, 298 K) of 3,6-bis(triethylene glycol monomethyl ether)-2,5-di(thiophen-2-yl)thieno[3,2-*b*]thiophene (T-g$_3$TT-T).

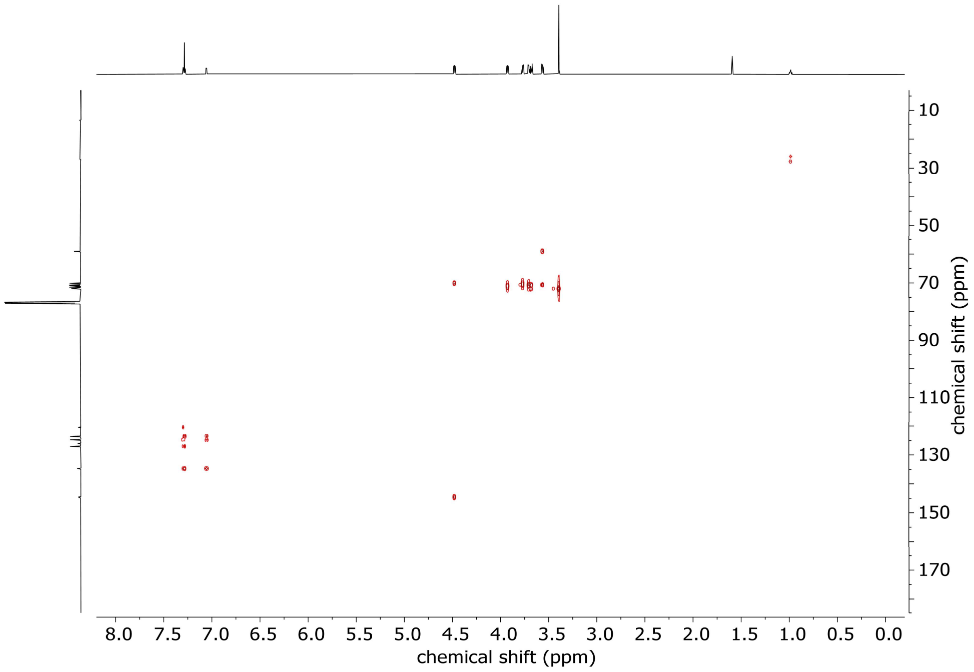

Figure S45. HMBC NMR spectrum ($CDCl_3$, 600/151 MHz, 298 K) of 3,6-bis(triethylene glycol monomethyl ether)-2,5-di(thiophen-2-yl)thieno[3,2-*b*]thiophene (T-g$_3$TT-T).

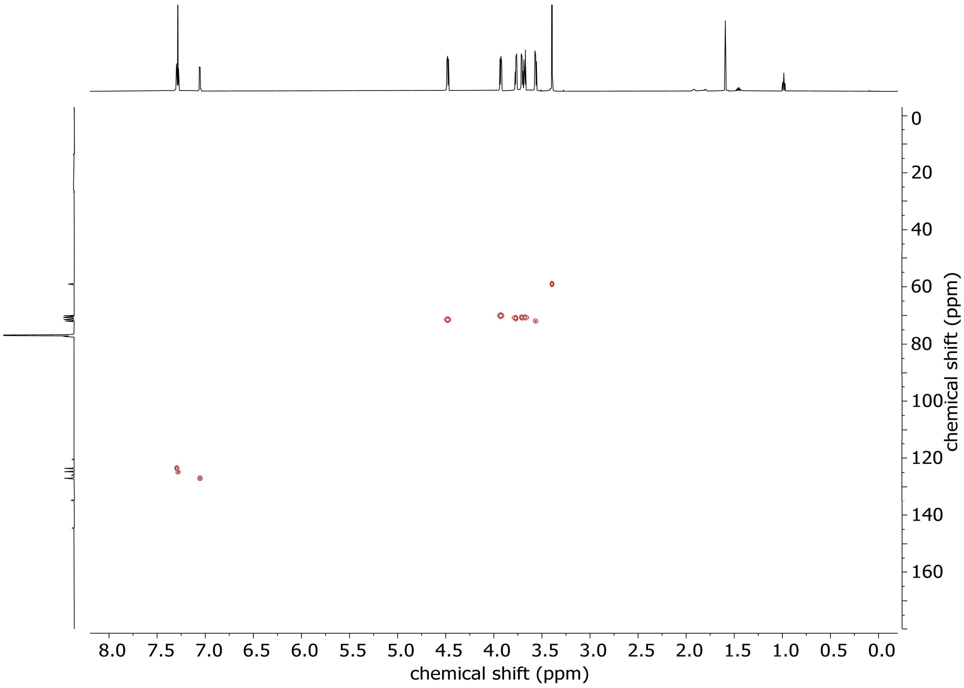

Figure S46. HSQC NMR spectrum ($CDCl_3$, 600/151 MHz, 298 K) of 3,6-bis(triethylene glycol monomethyl ether)-2,5-di(thiophen-2-yl)thieno[3,2-*b*]thiophene (T-g_3TT-T).

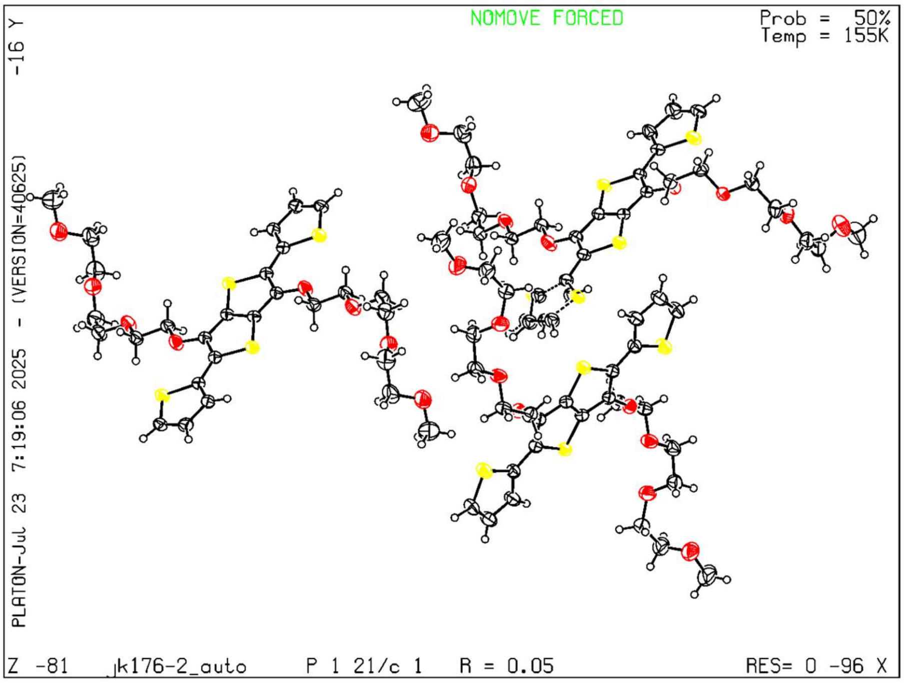

Figure S47. Single crystal X-ray ellipsoid plot (50 % probability) of 3,6-bis(triethylene glycol monomethyl ether)-2,5-di(thiophen-2-yl)thieno[3,2-*b*]thiophene (T-g_3TT-T). CCDC deposition number 2504799.

Table S16. Crystal data and structure refinement for 3,6-bis(triethylene glycol monomethyl ether)-2,5-di(thiophen-2-yl)thieno[3,2-*b*]thiophene (T-g$_3$TT-T).

Identification code	JK176-2_auto
Empirical formula	$C_{56}H_{72}O_{16}S_8$
Formula weight	1257.61
Temperature/K	155(2)
Crystal system	monoclinic
Space group	$P2_1/c$
a/Å	16.16324(13)
b/Å	12.87526(16)
c/Å	29.7506(3)
α/°	90
β/°	94.5717(9)
γ/°	90
Volume/Å^3	6171.58(11)
Z	4
ρ_{calc}g/cm^3	1.354
μ/mm^{-1}	3.222
F(000)	2656
Radiation	Cu Kα (λ = 1.54184)
2Θ range for data collection/°	5.486 to 151.164
Index ranges	$-14 \leq h \leq 19$, $-15 \leq k \leq 16$, $-37 \leq l \leq 36$
Reflections collected	54106
Independent reflections	12219 [R_{int} = 0.0343, R_{sigma} = 0.0260]
Data/restraints/parameters	12219/9/725
Goodness-of-fit on F^2	1.08
Final R indexes [I>=2σ (I)]	R_1 = 0.0518, wR_2 = 0.1296
Final R indexes [all data]	R_1 = 0.0634, wR_2 = 0.1363
Largest diff. peak/hole / e Å^{-3}	0.43/-0.81

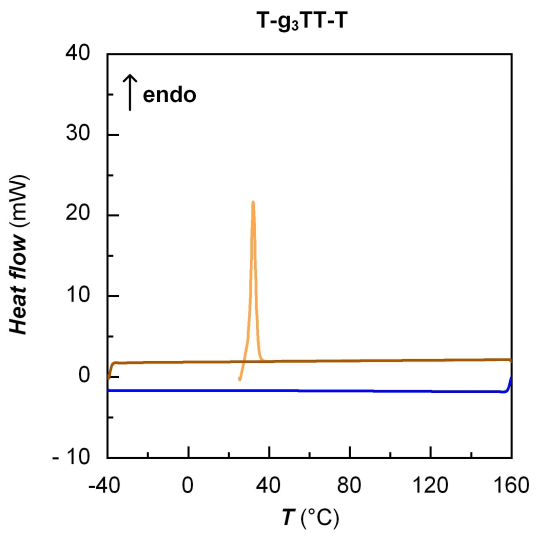

Figure S48. First and second differential scanning calorimetry heating thermograms of 3,6-bis(triethylene glycol monomethyl ether)-2,5-di(thiophen-2-yl)thieno[3,2-b]thiophene (T-g_3TT-T) given in pale orange and brown (-40 °C to 160 °C under nitrogen atmosphere using a heating rate of 10 °C min^{-1}) and first differential scanning calorimetry cooling thermograms given in blue (160 °C to -40 °C under nitrogen atmosphere using a cooling rate of 10 °C min^{-1}).

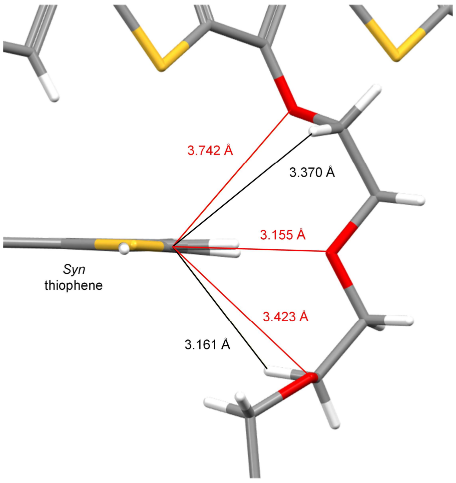

Figure S49. Shortest distances between the aromatic rings of *syn*-conformation thiophene and adjacent oligoethylene glycol chain atoms in crystals of 3,6-bis(triethylene glycol monomethyl ether)-2,5-di(thiophen-2-yl)thieno[3,2-*b*]thiophene (T-g_3TT-T). CCDC deposition number 2504799.

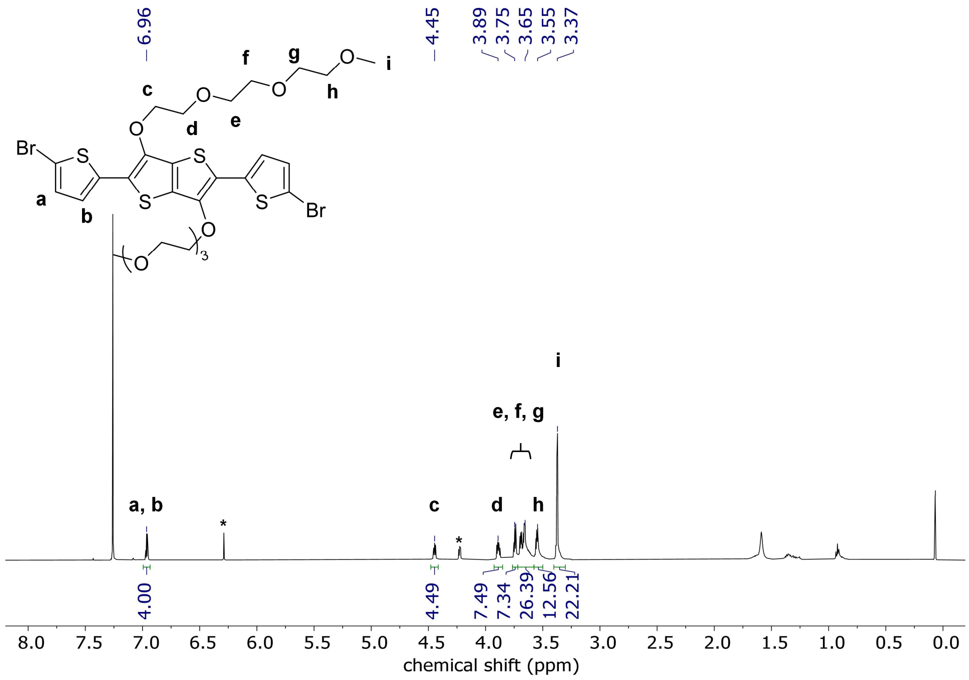

Figure S50. ^{1}H NMR spectrum (CDCl$_3$, 600 MHz, 298 K) of 2,5-bis(5-bromothiophen-2-yl)-3,6-bis triethylene glycol monomethyl ether)thieno[3,2-*b*]thiophene (BrT-g$_3$TT-TBr). Contamination with starting material g$_3$TT indicated by *.

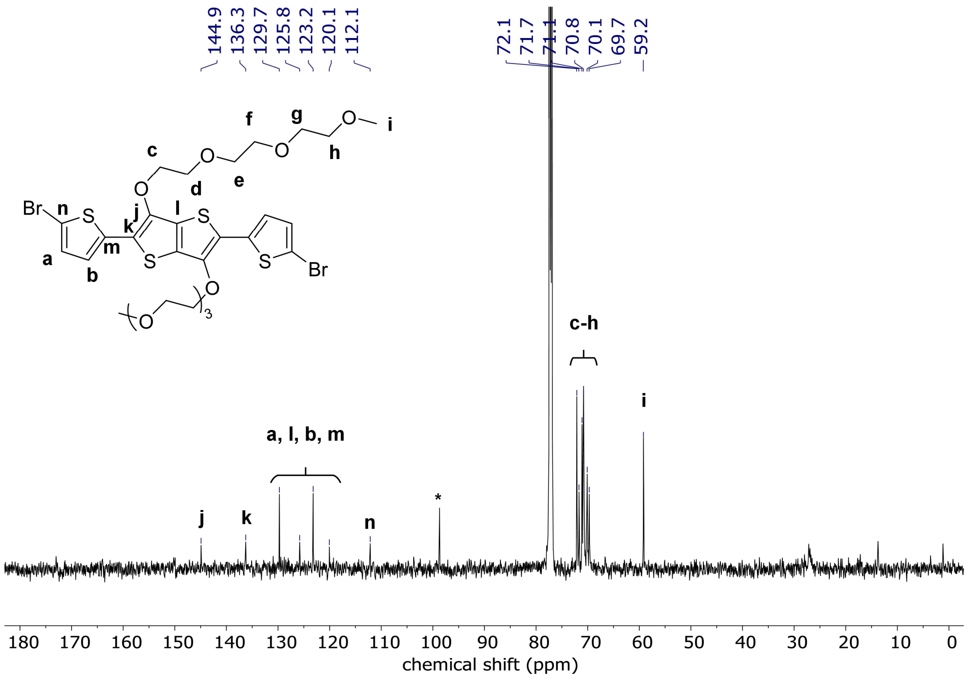

Figure S51. ^{13}C NMR spectrum (CDCl$_3$, 151 MHz, 298 K) of 2,5-bis(5-bromothiophen-2-yl)-3,6-bis-(triethylene glycol monomethyl ether))thieno[3,2-*b*]thiophene (BrT-g$_3$TT-TBr). Contamination with starting material g$_3$TT indicated by *.

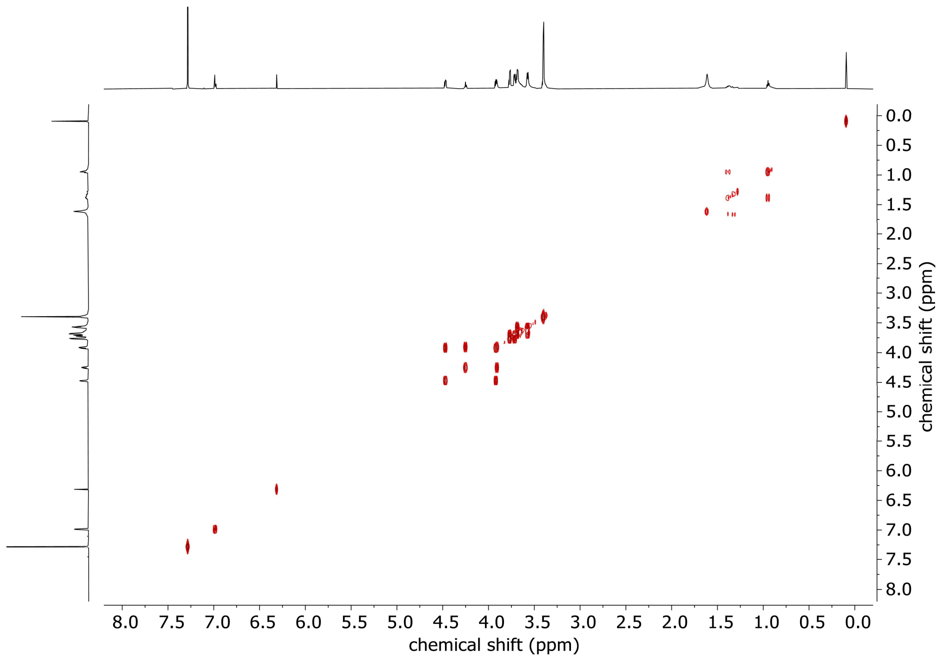

Figure S52. ^{1}H COSY NMR spectrum ($CDCl_3$, 600 MHz, 298 K) of 2,5-bis(5-bromothiophen-2-yl)-3,6-bis-(triethylene glycol monomethyl ether))thieno[3,2-*b*]thiophene (BrT-g_3TT-TBr).

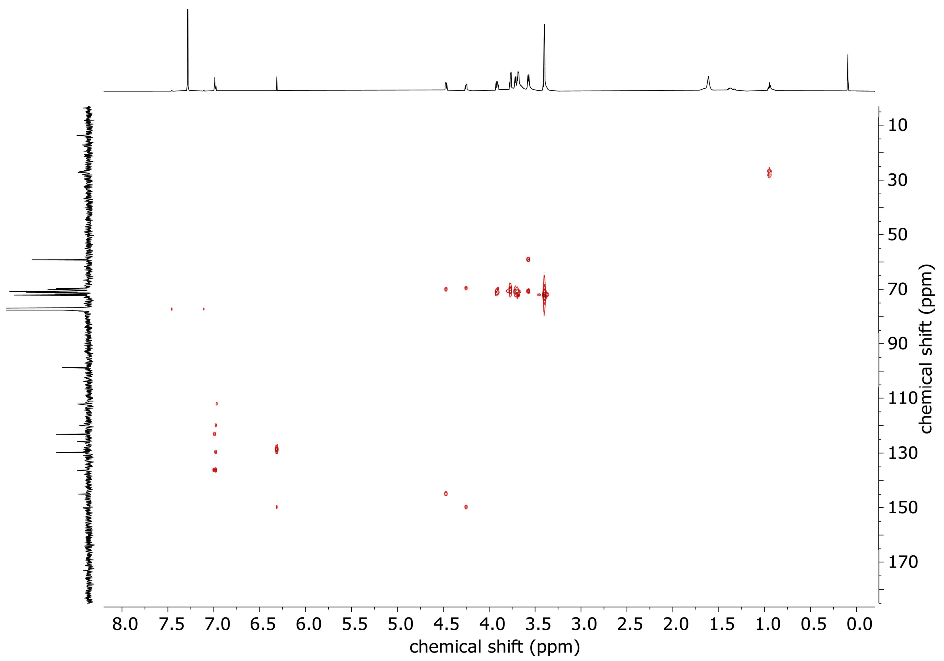

Figure S53. HMBC NMR spectrum ($CDCl_3$, 600/151 MHz, 298 K) of 2,5-bis(5-bromothiophen-2-yl)-3,6-bis triethylene glycol monomethyl ether)thieno[3,2-*b*]thiophene (BrT-g_3TT-TBr).

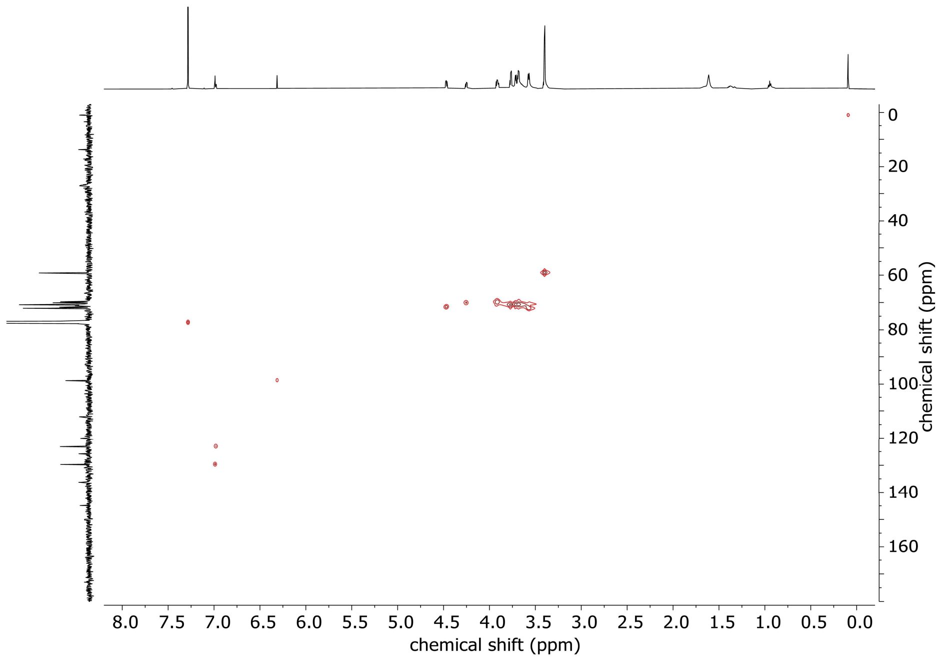

Figure S54. HSQC NMR spectrum ($CDCl_3$, 600/151 MHz, 298 K) of 2,5-bis(5-bromothiophen-2-yl)-3,6-bis triethylene glycol monomethyl ether)thieno[3,2-*b*]thiophene (BrT-g$_3$TT-TBr).

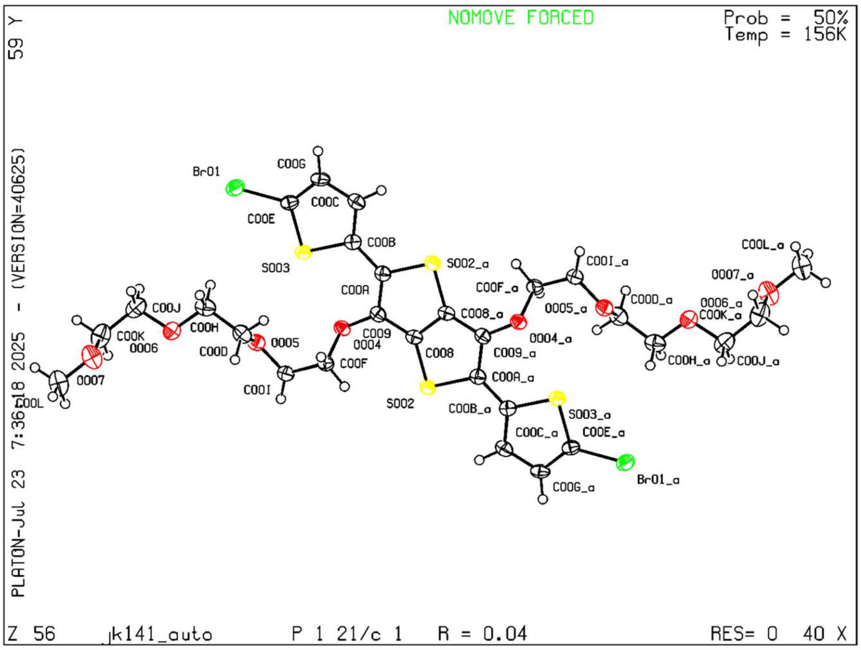

Figure S55. Single crystal X-ray ellipsoid plot (50 % probability) of 2,5-bis(5-bromothiophen-2-yl)-3,6-bis triethylene glycol monomethyl ether)thieno[3,2-*b*]thiophene (BrT-g$_3$TT-TBr). CCDC deposition number 2504798.

Table S17. Crystal data and structure refinement for 2,5-bis(5-bromothiophen-2-yl)-3,6-bis triethylene glycol monomethyl ether)thieno[3,2-*b*]thiophene (BrT-g_3TT-TBr).

Identification code	JK141_auto
Empirical formula	$C_{14}H_{17}BrO_4S_2$
Formula weight	393.3
Temperature/K	156.2(4)
Crystal system	monoclinic
Space group	$P2_1/c$
a/Å	4.38760(10)
b/Å	20.9619(4)
c/Å	17.1660(3)
α/°	90
β/°	93.641(2)
γ/°	90
Volume/Å^3	1575.61(5)
Z	4
ρ_{calc}g/cm^3	1.658
μ/mm^{-1}	6.15
F(000)	800
Radiation	Cu Kα (λ = 1.54184)
2Θ range for data collection/°	6.664 to 149.876
Index ranges	$-5 \leq h \leq 3$, $-25 \leq k \leq 25$, $-21 \leq l \leq 21$
Reflections collected	14222
Independent reflections	3137 [R_{int} = 0.0484, R_{sigma} = 0.0374]
Data/restraints/parameters	3137/0/191
Goodness-of-fit on F^2	1.042
Final R indexes [I>=2σ (I)]	R_1 = 0.0349, wR_2 = 0.0856
Final R indexes [all data]	R_1 = 0.0458, wR_2 = 0.0928
Largest diff. peak/hole / e Å^{-3}	0.61/-0.50

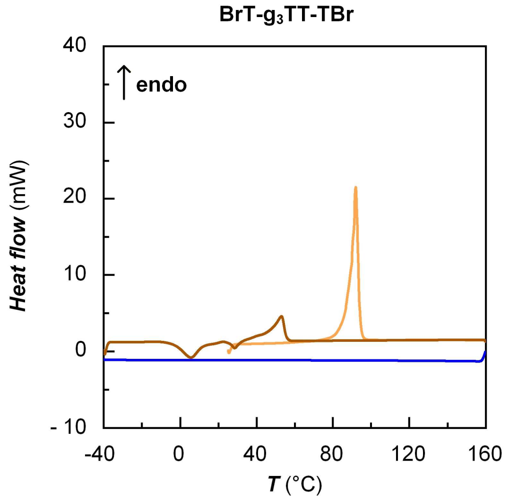

Figure S56. First and second differential scanning calorimetry heating thermograms of 2,5-bis(5-bromothiophen-2-yl)-3,6-bis triethylene glycol monomethyl ether)thieno[3,2-b]thiophene (BrT-g_3TT-TBr) given in pale orange and brown (-40 °C to 160 °C under nitrogen atmosphere using a heating rate of 10 °C min^{-1}) and first differential scanning calorimetry cooling thermograms given in blue (160 °C to -40 °C under nitrogen atmosphere using a cooling rate of 10 °C min^{-1}).

Bibliography

[1] O. V. Dolomanov, L. J. Bourhis, R. J. Gildea, J. A. K. Howard, H. Puschmann, *J. Appl. Crystallogr.* **2009**, *42*, 339-341.

[2] C. F. Macrae, I. Sovago, S. J. Cottrell, et al., *J. Appl. Crystallogr.* **2020**, *53*, 226-235.

[3] W. L. Jorgensen, D. S. Maxwell, J. Tirado-Rives, *J. Am. Chem. Soc.* **1996**, *118*, 11225-11236.

[4] G. W. T. M. J. Frisch, H. B. Schlegel, G. E. Scuseria, M. A. Robb, J. R. Cheeseman, G. Scalmani, V. Barone, B. Mennucci, G. A. Petersson, H. Nakatsuji, M. Caricato, X. Li, H. P. Hratchian, A. F. Izmaylov, J. Bloino, G. Zheng, J. L. Sonnenberg, M. Hada, M. Ehara, K. Toyota, R. Fukuda, J. Hasegawa, M. Ishida, T. Nakajima, Y. Honda, O. Kitao, H. Nakai, T. Vreven, J. A. Montgomery Jr., J. E. Peralta, F. Ogliaro, M. Bearpark, J. J. Heyd, E. Brothers, K. N. Kudin, V. N. Staroverov, R. Kobayashi, J. Normand, K. Raghavachari, A. Rendell, J. C. Burant, S. S. Iyengar, J. Tomasi, M. Cossi, N. Rega, J. M. Millam, M. Klene, J. E. Knox, J. B. Cross, V. Bakken, C. Adamo, J. Jaramillo, R. Gomperts, R. E. Stratmann, O. Yazyev, A. J. Austin, R. Cammi, C. Pomelli, J. W. Ochterski, R. L. Martin, K. Morokuma, V. G. Zakrzewski, G. A. Voth, P. Salvador, J. J. Dannenberg, S. Dapprich, A. D. Daniels, Ö. Farkas, J. B. Foresman, J. V. Ortiz, J. Cioslowski, and D. J. Fox, Wallingford CT, **2016**.

[5] N. Siemons, D. Pearce, C. Cendra, et al., *Adv. Mater.* **2022**, *34*, 2204258.

[6] R. S. Bhatta, Y. Y. Yimer, D. S. Perry, M. Tsige, *J. Phys. Chem. B.* **2013**, *117*, 10035-10045.

[7] M. Moreno, M. Casalegno, G. Raos, S. V. Meille, R. Po, *J. Phys. Chem. B.* **2010**, *114*, 1591-1602.

[8] C. M. Breneman, K. B. Wiberg, *J. Comput. Chem.* **1990**, *11*, 361-373.

[9] S. Moro, N. Siemons, O. Drury, et al., *ACS Nano* **2022**, *16*, 21303-21314.

[10] M. J. Abraham, T. Murtola, R. Schulz, et al., *SoftwareX* **2015**, *1-2*, 19-25.

[11] S. Páll, M. J. Abraham, C. Kutzner, B. Hess, E. Lindahl, in *Solving Software Challenges for Exascale* (Eds.: S. Markidis, E. Laure), Springer International Publishing, Cham, **2015**, pp. 3-27.

[12] B. Hess, C. Kutzner, D. van der Spoel, E. Lindahl, *J. Chem. Theory Comput.* **2008**, *4*, 435-447.

[13] G. Bussi, D. Donadio, M. Parrinello, *J. Chem. Phys.* **2007**, *126*.

[14] H. J. C. Berendsen, D. van der Spoel, R. van Drunen, *Comput. Phys. Commun.* **1995**, *91*, 43-56.

[15] J. Kimpel, Y. Kim, J. Asatryan, et al., *Chem. Sci.* **2024**, *15*, 7679-7688.

[16] J. Kimpel, Y. Kim, H. Schomaker, et al., *Sci. Adv.* **2025**, *11*, eadv8168.

[17] D. Zhu, J. Pons i Tarrés, J. Kimpel, et al., *Manuscript in preparation*.
